# Supplementary figures and images for: Genomic and phenotypic evolution of Escherichia coli in a novel citrate-only resource environment
Source: eLife. 2020 May 29;9:e55414. doi: 10.7554/eLife.55414 (PMC7299349; doi:10.7554/eLife.55414)

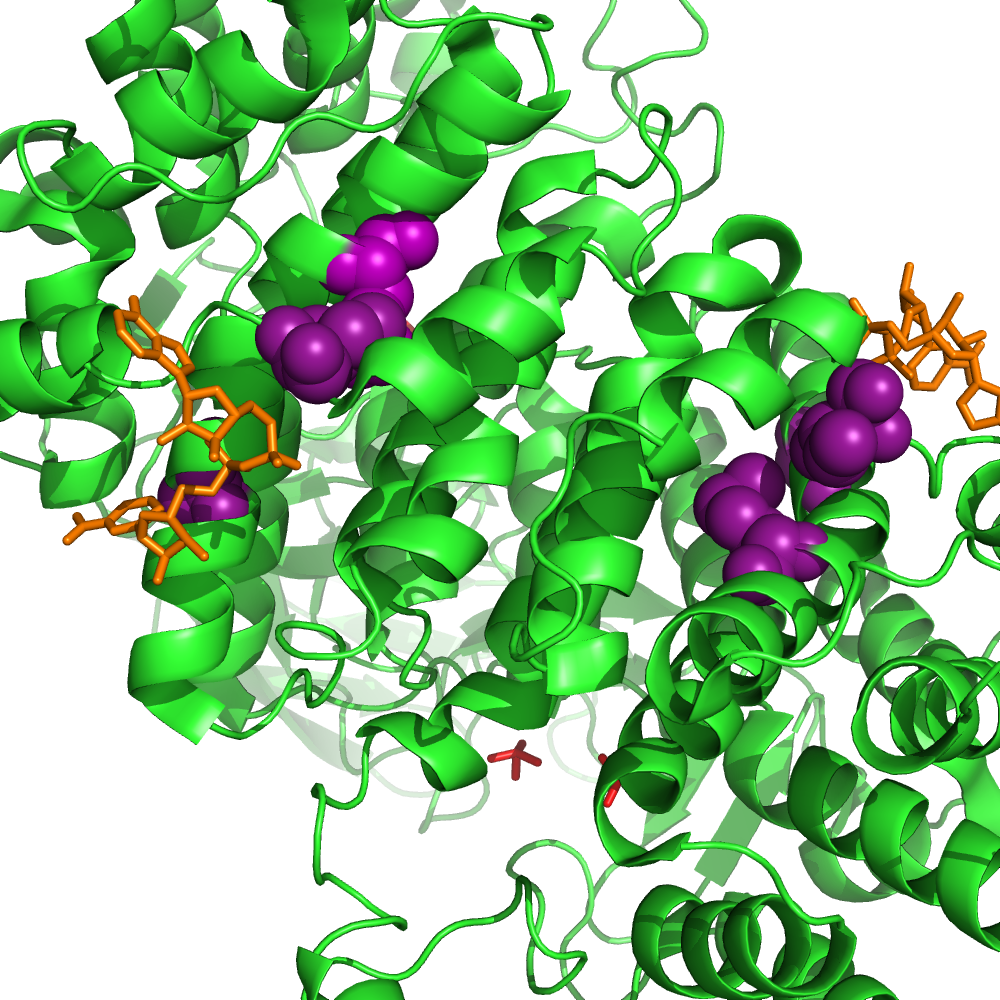

Supplement: Figure 11—figure supplement 1—source data 1. [file elife-55414-fig11-figsupp1-data1.zip › 1nxg-mapping.png]
