# Supplementary material for: Genomic and phenotypic evolution of Escherichia coli in a novel citrate-only resource environment
Source: eLife. 2020 May 29;9:e55414. doi: 10.7554/eLife.55414 (PMC7299349; doi:10.7554/eLife.55414)
Supplement: Supplementary file 1. [file elife-55414-supp1.docx]

| **Supplementary File S1. Key Resources Table** | | | | |
| --- | --- | --- | --- | --- |
| **Reagent type (species) or resource** | **Designation** | **Source or reference** | **Identifiers** | **Additional information** |
| Strain, strain background (*Escherichia coli*) | REL606 | Lenski et al. 1991 |  | LTEE ancestor |
| Strain, strain background (*Escherichia coli*) | CZB151 | Blount et al. 2012 |  | Cit^+^ Ara^−^ Ancestor in DM0 and DM25 evolution experiments |
| Strain, strain background (*Escherichia coli*) | CZB152 | Blount et al. 2012 |  | Cit^+^ Ara^−^ Ancestor in DM0 and DM25 evolution experiments |
| Strain, strain background (*Escherichia coli*) | CZB154 | Blount et al. 2012 |  | Cit^+^ Ara^−^ Ancestor in DM0 and DM25 evolution experiments |
| Strain, strain background (*Escherichia coli*) | ZDB67 | This paper |  | Cit^+^ Ara^+^ Ancestor in DM0 and DM25 evolution experiments |
| Strain, strain background (*Escherichia coli*) | ZDB68 | This paper |  | Cit^+^ Ara^+^ Ancestor in DM0 and DM25 evolution experiments |
| Strain, strain background (*Escherichia coli*) | ZDB69 | This paper |  | Cit^+^ Ara^+^ Ancestor in DM0 and DM25 evolution experiments |
| Strain, strain background (*Escherichia coli*) | ZDBp871 | This paper |  | Cit+ clone isolated from population evolved for 2,500 generations in DM0, descended from CZB151. |
| Strain, strain background (*Escherichia coli*) | ZDBp875 | This paper |  | Cit+ clone isolated from population evolved for 2,500 generations in DM0, descended from CZB151. |
| Strain, strain background (*Escherichia coli*) | ZDBp877 | This paper |  | Cit+ clone isolated from population evolved for 2,500 generations in DM0, descended from CZB152. |
| Strain, strain background (*Escherichia coli*) | ZDBp880 | This paper |  | Cit+ clone isolated from population evolved for 2,500 generations in DM0, descended from CZB152. |
| Strain, strain background (*Escherichia coli*) | ZDBp883 | This paper |  | Cit+ clone isolated from population evolved for 2,500 generations in DM0, descended from CZB154. |
| Strain, strain background (*Escherichia coli*) | ZDBp886 | This paper |  | Cit+ clone isolated from population evolved for 2,500 generations in DM0, descended from CZB154. |
| Strain, strain background (*Escherichia coli*) | ZDBp889 | This paper |  | Cit+ clone isolated from population evolved for 2,500 generations in DM0, descended from ZDB67. |
| Strain, strain background (*Escherichia coli*) | ZDBp892 | This paper |  | Cit+ clone isolated from population evolved for 2,500 generations in DM0, descended from ZDB67. |
| Strain, strain background (*Escherichia coli*) | ZDBp895 | This paper |  | Cit+ clone isolated from population evolved for 2,500 generations in DM0, descended from ZDB68. |
| Strain, strain background (*Escherichia coli*) | ZDBp898 | This paper |  | Cit+ clone isolated from population evolved for 2,500 generations in DM0, descended from ZDB68. |
| Strain, strain background (*Escherichia coli*) | ZDBp901 | This paper |  | Cit+ clone isolated from population evolved for 2,500 generations in DM0, descended from ZDB69. |
| Strain, strain background (*Escherichia coli*) | ZDBp904 | This paper |  | Cit+ clone isolated from population evolved for 2,500 generations in DM0, descended from ZDB69. |
| Strain, strain background (*Escherichia coli*) | ZDBp910 | This paper |  | Cit+ clone isolated from population evolved for 2,500 generations in DM25, descended from CZB151. |
| Strain, strain background (*Escherichia coli*) | ZDBp911 | This paper |  | Cit+ clone isolated from population evolved for 2,500 generations in DM25, descended from CZB151. |
| Strain, strain background (*Escherichia coli*) | ZDBp912 | This paper |  | Cit+ clone isolated from population evolved for 2,500 generations in DM25, descended from CZB152. |
| Strain, strain background (*Escherichia coli*) | ZDBp913 | This paper |  | Cit+ clone isolated from population evolved for 2,500 generations in DM25, descended from CZB152. |
| Strain, strain background (*Escherichia coli*) | ZDBp914 | This paper |  | Cit+ clone isolated from population evolved for 2,500 generations in DM25, descended from CZB154. |
| Strain, strain background (*Escherichia coli*) | ZDBp915 | This paper |  | Cit+ clone isolated from population evolved for 2,500 generations in DM25, descended from CZB154. |
| Strain, strain background (*Escherichia coli*) | ZDBp916 | This paper |  | Cit+ clone isolated from population evolved for 2,500 generations in DM25, descended from ZDB67. |
| Strain, strain background (*Escherichia coli*) | ZDBp917 | This paper |  | Cit+ clone isolated from population evolved for 2,500 generations in DM25, descended from ZDB67. |
| Strain, strain background (*Escherichia coli*) | ZDBp918 | This paper |  | Cit+ clone isolated from population evolved for 2,500 generations in DM25, descended from ZDB68. |
| Strain, strain background (*Escherichia coli*) | ZDBp919 | This paper |  | Cit+ clone isolated from population evolved for 2,500 generations in DM25, descended from ZDB68. |
| Strain, strain background (*Escherichia coli*) | ZDBp920 | This paper |  | Cit+ clone isolated from population evolved for 2,500 generations in DM25, descended from ZDB69. |
| Strain, strain background (*Escherichia coli*) | ZDBp921 | This paper |  | Cit+ clone isolated from population evolved for 2,500 generations in DM25, descended from ZDB69. |
| Biological Sample (*Escherichia coli*) | DM0−1 | This paper | ZDBp799 | Cit+ mixed population evolved for 2,500 generations in DM0, descended from CZB151. |
| Biological Sample (*Escherichia coli*) | DM0−2 | This paper | ZDBp800 | Cit+ mixed population evolved for 2,500 generations in DM0, descended from CZB151. |
| Biological Sample (*Escherichia coli*) | DM0−3 | This paper | ZDBp801 | Cit+ mixed population evolved for 2,500 generations in DM0, descended from CZB152. |
| Biological Sample (*Escherichia coli*) | DM0−4 | This paper | ZDBp802 | Cit+ mixed population evolved for 2,500 generations in DM0, descended from CZB152. |
| Biological Sample (*Escherichia coli*) | DM0−5 | This paper | ZDBp803 | Cit+ mixed population evolved for 2,500 generations in DM0, descended from CZB154. |
| Biological Sample (*Escherichia coli*) | DM0−6 | This paper | ZDBp804 | Cit+ mixed population evolved for 2,500 generations in DM0, descended from CZB154. |
| Biological Sample (*Escherichia coli*) | DM0+1 | This paper | ZDBp805 | Cit+ mixed population evolved for 2,500 generations in DM0, descended from ZDB67. |
| Biological Sample (*Escherichia coli*) | DM0+2 | This paper | ZDBp806 | Cit+ mixed population evolved for 2,500 generations in DM0, descended from ZDB67. |
| Biological Sample (*Escherichia coli*) | DM0+3 | This paper | ZDBp807 | Cit+ mixed population evolved for 2,500 generations in DM0, descended from ZDB68. |
| Biological Sample (*Escherichia coli*) | DM0+4 | This paper | ZDBp808 | Cit+ mixed population evolved for 2,500 generations in DM0, descended from ZDB68. |
| Biological Sample (*Escherichia coli*) | DM0+5 | This paper | ZDBp809 | Cit+ mixed population evolved for 2,500 generations in DM0, descended from ZDB69. |
| Biological Sample (*Escherichia coli*) | DM0+6 | This paper | ZDBp810 | Cit+ mixed population evolved for 2,500 generations in DM0, descended from ZDB69. |
| Biological Sample (*Escherichia coli*) | DM25−1 | This paper | ZDBp745 | Cit+ mixed population evolved for 2,500 generations in DM25, descended from CZB151. |
| Biological Sample (*Escherichia coli*) | DM25−2 | This paper | ZDBp746 | Cit+ mixed population evolved for 2,500 generations in DM25, descended from CZB151. |
| Biological Sample (*Escherichia coli*) | DM25−3 | This paper | ZDBp747 | Cit+ mixed population evolved for 2,500 generations in DM25, descended from CZB152. |
| Biological Sample (*Escherichia coli*) | DM25−4 | This paper | ZDBp748 | Cit+ mixed population evolved for 2,500 generations in DM25, descended from CZB152. |
| Biological Sample (*Escherichia coli*) | DM25−5 | This paper | ZDBp749 | Cit+ mixed population evolved for 2,500 generations in DM25, descended from CZB154. |
| Biological Sample (*Escherichia coli*) | DM25−6 | This paper | ZDBp750 | Cit+ mixed population evolved for 2,500 generations in DM25, descended from CZB154. |
| Biological Sample (*Escherichia coli*) | DM25+1 | This paper | ZDBp751 | Cit+ mixed population evolved for 2,500 generations in DM25, descended from ZDB67. |
| Biological Sample (*Escherichia coli*) | DM25+2 | This paper | ZDBp752 | Cit+ mixed population evolved for 2,500 generations in DM25, descended from ZDB67. |
| Biological Sample (*Escherichia coli*) | DM25+3 | This paper | ZDBp753 | Cit+ mixed population evolved for 2,500 generations in DM25, descended from ZDB68. |
| Biological Sample (*Escherichia coli*) | DM25+4 | This paper | ZDBp754 | Cit+ mixed population evolved for 2,500 generations in DM25, descended from ZDB68. |
| Biological Sample (*Escherichia coli*) | DM25+5 | This paper | ZDBp755 | Cit+ mixed population evolved for 2,500 generations in DM25, descended from ZDB69. |
| Biological Sample (*Escherichia coli*) | DM25+6 | This paper | ZDBp756 | Cit+ mixed population evolved for 2,500 generations in DM25, descended from ZDB69. |
| Strain, strain background (*Escherichia coli*) | CZB151(RM4.6.2) | This paper |  | CZB151 transformed with low-copy plasmid RM4.6.2). |
| Strain, strain background (*Escherichia coli*) | CZB152(RM4.6.2) | This paper |  | CZB152 transformed with low-copy plasmid RM4.6.2). |
| Strain, strain background (*Escherichia coli*) | ZDB67(RM4.6.2) | This paper |  | ZDB67 transformed with low-copy plasmid RM4.6.2). |
| Strain, strain background (*Escherichia coli*) | ZDB68(RM4.6.2) | This paper |  | ZDB68 transformed with low-copy plasmid RM4.6.2). |
| Strain, strain background (*Escherichia coli*) | CZB151(PSB3K3) | This paper |  | CZB151 transformed with low-copy plasmid PSB3K3). |
| Strain, strain background (*Escherichia coli*) | CZB152(PSB3K3) | This paper |  | CZB152 transformed with low-copy plasmid PSB3K3). |
| Strain, strain background (*Escherichia coli*) | ZDB67(PSB3K3) | This paper |  | ZDB67 transformed with low-copy plasmid PSB3K3). |
| Strain, strain background (*Escherichia coli*) | ZDB68(PSB3K3) | This paper |  | ZDB68 transformed with low-copy plasmid PSB3K3). |
| Strain, strain (*Escherichia coli*) | REL11364 | This paper |  | Clone isolated from LTEE population Ara-3 after 50,000 generations of evolution in DM25. |
| Recombinant DNA reagent | pSB3K3 | http://parts.igem.org/Part:pSB3K3 |  |  |
| Recombinant DNA reagent | RM4.6.2 (plasmid) | This paper |  | Low-copy, recombinant plasmid derived from pSB3K3 and containing a copy of *maeA* under the control of a strong constitutive promoter (P089-R052) and ribosome-binding site |
| Gene (*Escherichia coli*) | maeA |  | sfcA, EG10948, (EcoCyc),  b1479,  ECK1473,  P26616 (UniProt) | malate dehydrogenase, NAD-requiring |
| Commercial assay or kit | LIVE/DEAD BacLight Viability Kit | ThermoFisher | ThermoFisher #L7007 |  |
| Software, algorithm | SuperSegger | <https://github.com/wiggins-lab/SuperSegger/wiki> | SCR_018532 |  |
| Commercial assay or kit | Qiagen Genomic-tip 100/G DNA extraction kit | Qiagen | Qiagen #10243 |  |
| Commercial assay or kit | PureLink Genomic DNA mini kit | Invitrogen | Invitrogen #K182001 |  |
| Commercial assay or kit | KAPA Low Throughput Library Preparation kit | Roche | Roche  #KK8232 |  |
| Software, algorithm | Timmomatic version 0.38 | <http://www.usadellab.org/cms/?page=trimmomatic> | SCR_011848 |  |
| Peptide, recombinant protein | NEB  dsDNA Fragmentase | New England Biolabs | NEB #M0348S |  |
| Software, algorithm | breseq version 0.33.2 | <https://barricklab.org/twiki/bin/view/Lab/ToolsBacterialGenomeResequencing> | SCR_010810 |  |
| Commercial assay or kit | Qiagen RNeasy MiniKit | Qiagen | Qiagen #74104 |  |
| Commercial assay or kit | Qiagen RNase-free DNase set | Qiagen | Qiagen  #79254 |  |
| Software, algorithm | kallisto version 0.44 | <http://pachterlab.github.io/kallisto/> | SCR_016582 |  |
| Software, algorithm | sleuth | <https://pachterlab.github.io/sleuth/> | SCR_002555 |  |
| Commercial assay or kit | NEB Gibson Assembly Cloning kit | New England Biolabs | NEB #E5510S |  |
| Other | Millipore membrane filter | Millipore | Millipore #VSWP01300 |  |
