# Supplementary material for: Genomic and phenotypic evolution of Escherichia coli in a novel citrate-only resource environment
Source: eLife. 2020 May 29;9:e55414. doi: 10.7554/eLife.55414 (PMC7299349; doi:10.7554/eLife.55414)
Supplement: Supplementary file 5. [file elife-55414-supp5.zip › S4File_genomes-by-environment/DM25-html/ZDBp910_minus_CZB151.html]

Mutation Comparison


| Predicted mutations | | | | |
| --- | --- | --- | --- | --- |
| position | mutation | annotation | gene | description |
| 464,051 | IS*150* (+) +3 bp | coding (274‑276/528 nt) | *priC* ← | primosomal replication protein N'' |
| 568,370 | Δ19,700 bp | IS*150*‑mediated | *[ECB\_00524]*–*ybdK* | **19 genes***[ECB\_00524]*, *yhhI*, *ECB\_00526*, *ECB\_00527*, *ECB\_00528*, *ECB\_00529*, *ECB\_00530*, *cusS*, *cusR*, *cusC*, *ylcC*, *cusB*, *cusA*, *pheP*, *ybdG*, *nfnB*, *ybdF*, *ybdJ*, *ybdK* *[ECB\_00524]*, *yhhI*, *ECB\_00526*, *ECB\_00527*, *ECB\_00528*, *ECB\_00529*, *ECB\_00530*, *cusS*, *cusR*, *cusC*, *ylcC*, *cusB*, *cusA*, *pheP*, *ybdG*, *nfnB*, *ybdF*, *ybdJ*, *ybdK* |
| 665,709 | Δ1 bp | intergenic (‑490/‑47) | *rihA* ← / → *insJ‑2* | ribonucleoside hydrolase 1/IS150 hypothetical protein |
| 736,640 | IS*1* (–) +9 bp | intergenic (‑360/‑341) | *gltA* ← / → *sdhC* | citrate synthase/succinate dehydrogenase cytochrome b556 large membrane subunit |
| 1,968,587 | IS*150* (–) +3 bp | intergenic (+14/+46) | *tyrP* → / ← *yecA* | tyrosine transporter/conserved metal‑binding protein |
| 2,112,669 | IS*150* (–) +3 bp | coding (16‑18/918 nt) | *yehZ* ← | predicted transporter subunit: periplasmic‑binding component of ABC superfamily |
| 2,264,348 | IS*186* (–) +8 bp | coding (129‑136/963 nt) | *menC* ← | O‑succinylbenzoate synthase |
| 2,465,970 | IS*186* (–) +9 bp | coding (584‑592/618 nt) | *hyfA* → | hydrogenase 4, 4Fe‑4S subunit |
| 2,466,822 | IS*150* (+) +4 bp | coding (819‑822/2019 nt) | *hyfB* → | NADH dehydrogenase subunit N |
| 3,109,394 | IS*150* (–) +3 bp | coding (245‑247/663 nt) | *yqjA* → | conserved inner membrane protein |
| position | mutation | annotation | gene | description |
| 3,172,540 | IS*150* (–) +3 bp | intergenic (‑39/+68) | *nlpI* ← / ← *pnp* | hypothetical protein/polynucleotide phosphorylase/polyadenylase |
| 3,501,576 | IS*150* (+) +3 bp | intergenic (‑35/‑354) | *yhiO* ← / → *uspA* | universal stress protein UspB/universal stress global response regulator |
| 3,574,917 | Δ7,306 bp | IS*150*‑mediated | *[tag]*–*hokA* | *[tag]*, *yiaC*, *bisC*, *yiaD*, *tkrA*, *yiaF*, *yiaG*, *cspA*, *hokA* |
| 3,770,320 | G→C | intergenic (‑93/+147) | *yidB* ← / ← *gyrB* | hypothetical protein/DNA gyrase subunit B |
| 4,123,868 | IS*150* (–) +3 bp | coding (1562‑1564/1602 nt) | *aceB* → | malate synthase |
