# Supplementary material for: Genomic and phenotypic evolution of Escherichia coli in a novel citrate-only resource environment
Source: eLife. 2020 May 29;9:e55414. doi: 10.7554/eLife.55414 (PMC7299349; doi:10.7554/eLife.55414)
Supplement: Supplementary file 5. [file elife-55414-supp5.zip › S4File_genomes-by-environment/DM25-html/ZDBp919_minus_ZDB68.html]

Mutation Comparison


| Predicted mutations | | | | |
| --- | --- | --- | --- | --- |
| position | mutation | annotation | gene | description |
| 665,709 | Δ1 bp | intergenic (‑490/‑47) | *rihA* ← / → *insJ‑2* | ribonucleoside hydrolase 1/IS150 hypothetical protein |
| 972,660 | G→T | intergenic (‑80/+325) | *focA* ← / ← *ycaO* | formate transporter/hypothetical protein |
| 1,457,389 | Δ11,725 bp | between IS*150* | *hrpA*–*insJ‑2* | *hrpA*, *ydcF*, *aldA*, *gapC*, *insA‑12*, *insB‑12*, *cybB*, *ydcA*, *hokB*, *mokB*, *insK‑2*, *insJ‑2* |
| 2,133,584 | T→G | K4T (AAG→ACG) | *mglB* ← | methyl‑galactoside transporter subunit |
| 2,264,346 | IS*186* (+) +8 bp | coding (131‑138/963 nt) | *menC* ← | O‑succinylbenzoate synthase |
| 2,659,020 | IS*3* (–) +3 bp :: +TCA | intergenic (‑212/+21) | *csrA* ← / ← *alaS* | carbon storage regulator/alanyl‑tRNA synthetase |
| 3,825,950 | +GG | intergenic (+4/‑50) | *kup* → / → *insJ‑5* | potassium transporter/IS150 hypothetical protein |
| 3,827,398 | +C | intergenic (+29/+983) | *insK‑5* → / ← *yieP* | IS150 putative transposase/predicted transcriptional regulator |
| 4,122,539 | G→A | W78\* (TGG→TAG) | *aceB* → | malate synthase |
| 4,343,098 | Δ1,446 bp | IS*150*‑mediated | *insK‑2*–*insJ‑2* | *insK‑2*, *insJ‑2* |
| position | mutation | annotation | gene | description |
| 4,456,970 | +AAG :: IS*150* (+) +2 bp | intergenic (‑32/+17) | *yjiX* ← / ← *yjiY* | hypothetical protein/predicted inner membrane protein |
