# Supplementary material for: Genomic and phenotypic evolution of Escherichia coli in a novel citrate-only resource environment
Source: eLife. 2020 May 29;9:e55414. doi: 10.7554/eLife.55414 (PMC7299349; doi:10.7554/eLife.55414)
Supplement: Supplementary file 5. [file elife-55414-supp5.zip › S4File_genomes-by-environment/DM25-html/ZDBp911_minus_CZB151.html]

Mutation Comparison


| Predicted mutations | | | | |
| --- | --- | --- | --- | --- |
| position | mutation | annotation | gene | description |
| 573,826 | IS*3* (–) +5 bp :: +T | coding (1419‑1423/1449 nt) | *cusS* ← | sensory histidine kinase in two‑component regulatory system with CusR, senses copper ions |
| 736,587 | IS*1* (–) +9 bp | intergenic (‑307/‑394) | *gltA* ← / → *sdhC* | citrate synthase/succinate dehydrogenase cytochrome b556 large membrane subunit |
| 1,429,178 | A→G | intergenic (‑121/+807) | *ynaE* ← / ← *ynaF* | predicted DNA‑binding transcriptional regulator/stress‑induced protein, ATP‑binding protein |
| 1,457,389 | Δ11,725 bp | between IS*150* | *hrpA*–*insJ‑2* | *hrpA*, *ydcF*, *aldA*, *gapC*, *insA‑12*, *insB‑12*, *cybB*, *ydcA*, *hokB*, *mokB*, *insK‑2*, *insJ‑2* |
| 2,132,618 | IS*1* (+) +9 bp | coding (969‑977/999 nt) | *mglB* ← | methyl‑galactoside transporter subunit |
| 2,264,348 | IS*186* (–) +8 bp | coding (129‑136/963 nt) | *menC* ← | O‑succinylbenzoate synthase |
| 4,123,868 | IS*150* (–) +3 bp | coding (1562‑1564/1602 nt) | *aceB* → | malate synthase |
| 4,456,970 | IS*150* (–) +3 bp | intergenic (‑32/+16) | *yjiX* ← / ← *yjiY* | hypothetical protein/predicted inner membrane protein |
