# Supplementary material for: Genomic and phenotypic evolution of Escherichia coli in a novel citrate-only resource environment
Source: eLife. 2020 May 29;9:e55414. doi: 10.7554/eLife.55414 (PMC7299349; doi:10.7554/eLife.55414)
Supplement: Supplementary file 5. [file elife-55414-supp5.zip › S4File_genomes-by-environment/DM25-html/ZDBp918_minus_ZDB68.html]

Mutation Comparison


| Predicted mutations | | | | |
| --- | --- | --- | --- | --- |
| position | mutation | annotation | gene | description |
| 432,359 | IS*150* (+) +4 bp | intergenic (‑126/‑27) | *insL‑2* ← / → *lon* | putative transposase insL for insertion sequence IS186/DNA‑binding ATP‑dependent protease La |
| 464,051 | IS*150* (+) +3 bp | coding (274‑276/528 nt) | *priC* ← | primosomal replication protein N'' |
| 736,294 | A→G | intergenic (‑14/‑695) | *gltA* ← / → *sdhC* | citrate synthase/succinate dehydrogenase cytochrome b556 large membrane subunit |
| 1,173,387 | IS*150* (+) +3 bp | intergenic (+223/‑70) | *ycfH* → / → *ptsG* | predicted metallodependent hydrolase/fused glucose‑specific PTS enzymes: IIB component/IIC component |
| 1,457,389 | Δ11,725 bp | between IS*150* | *hrpA*–*insJ‑2* | *hrpA*, *ydcF*, *aldA*, *gapC*, *insA‑12*, *insB‑12*, *cybB*, *ydcA*, *hokB*, *mokB*, *insK‑2*, *insJ‑2* |
| 4,122,222 | IS*1* (+) +9 bp | intergenic (+184/‑77) | *metA* → / → *aceB* | homoserine O‑succinyltransferase/malate synthase |
| 4,201,689 | IS*150* (–) +3 bp | coding (892‑894/1314 nt) | *gltP* → | glutamate/aspartate:proton symporter |
