# Supplementary material for: Genomic and phenotypic evolution of Escherichia coli in a novel citrate-only resource environment
Source: eLife. 2020 May 29;9:e55414. doi: 10.7554/eLife.55414 (PMC7299349; doi:10.7554/eLife.55414)
Supplement: Supplementary file 5. [file elife-55414-supp5.zip › S4File_genomes-by-environment/DM25-html/ZDBp914_minus_CZB154.html]

Mutation Comparison


| Predicted mutations | | | | |
| --- | --- | --- | --- | --- |
| position | mutation | annotation | gene | description |
| 16,990 | +A | intergenic (‑32/‑50) | *mokC* ← / → *insJ‑2* | regulatory protein for HokC, overlaps CDS of hokC/IS150 hypothetical protein |
| 363,636 | G→A | K28K (AAG→AAA) | *yaiV* → | predicted DNA‑binding transcriptional regulator |
| 544,481 | IS*3* (–) +5 bp :: +T | coding (250‑254/552 nt) | *ybcL* → | predicted kinase inhibitor |
| 549,926 | Δ39,972 bp | between IS*1* | *ECB\_00510*–*insA‑7* | **35 genes***ECB\_00510*, *nohB*, *ECB\_00512*, *ECB\_00513*, *ECB\_00514*, *ECB\_00515*, *ECB\_00516*, *ECB\_00517*, *appY*, *ompT*, *envY*, *ybcH*, *nfrA*, *ECB\_00524*, *yhhI*, *ECB\_00526*, *ECB\_00527*, *ECB\_00528*, *ECB\_00529*, *ECB\_00530*, *cusS*, *cusR*, *cusC*, *ylcC*, *cusB*, *cusA*, *pheP*, *ybdG*, *nfnB*, *ybdF*, *ybdJ*, *ybdK*, *insJ‑1*, *insB‑7*, *insA‑7* *ECB\_00510*, *nohB*, *ECB\_00512*, *ECB\_00513*, *ECB\_00514*, *ECB\_00515*, *ECB\_00516*, *ECB\_00517*, *appY*, *ompT*, *envY*, *ybcH*, *nfrA*, *ECB\_00524*, *yhhI*, *ECB\_00526*, *ECB\_00527*, *ECB\_00528*, *ECB\_00529*, *ECB\_00530*, *cusS*, *cusR*, *cusC*, *ylcC*, *cusB*, *cusA*, *pheP*, *ybdG*, *nfnB*, *ybdF*, *ybdJ*, *ybdK*, *insJ‑1*, *insB‑7*, *insA‑7* |
| 736,642 | IS*1* (–) +7 bp | intergenic (‑362/‑341) | *gltA* ← / → *sdhC* | citrate synthase/succinate dehydrogenase cytochrome b556 large membrane subunit |
| 1,729,737 | T→A | intergenic (‑48/+702) | *insJ‑2* ← / ← *ydhZ* | IS150 hypothetical protein/hypothetical protein |
| 2,043,155 | G→A | R700C (CGC→TGC) | *wzc* ← | protein‑tyrosine kinase |
| 2,132,724 | IS*150* (+) +3 bp | coding (869‑871/999 nt) | *mglB* ← | methyl‑galactoside transporter subunit |
| 2,694,135 | IS*150* (+) +3 bp | coding (135‑137/2079 nt) | *fhlA* → | DNA‑binding transcriptional activator |
| 3,391,153 | T→C | intergenic (‑247/‑132) | *yhgE* ← / → *pckA* | predicted inner membrane protein/phosphoenolpyruvate carboxykinase |
| position | mutation | annotation | gene | description |
| 3,429,514 | A→C | F358C (TTC→TGC) | *glgC* ← | glucose‑1‑phosphate adenylyltransferase |
| 3,524,609 | G→T | G211G (GGC→GGA) | *yhiW* ← | DNA‑binding transcriptional activator |
| 3,582,219 | +A | intergenic (‑29/‑51) | *hokA* ← / → *insJ‑4* | toxic polypeptide, small/IS150 hypothetical protein |
| 4,122,388 | Δ4 bp | coding (82‑85/1602 nt) | *aceB* → | malate synthase |
| 4,256,901 | (CGCGG)3→2 | intergenic (‑768/‑1042) | *dcuR* ← / → *yjdI* | DNA‑binding response regulator in two‑component regulatory system with DcuS/hypothetical protein |
