# Supplementary material for: Genomic and phenotypic evolution of Escherichia coli in a novel citrate-only resource environment
Source: eLife. 2020 May 29;9:e55414. doi: 10.7554/eLife.55414 (PMC7299349; doi:10.7554/eLife.55414)
Supplement: Supplementary file 5. [file elife-55414-supp5.zip › S4File_genomes-by-environment/DM25-html/ZDBp913_minus_CZB152.html]

Mutation Comparison


| Predicted mutations | | | | |
| --- | --- | --- | --- | --- |
| position | mutation | annotation | gene | description |
| 300,764 | IS*150* (–) +3 bp | coding (577‑579/1671 nt) | *betA* ← | choline dehydrogenase |
| 574,297 | +TGA :: IS*3* (+) +3 bp | coding (950‑952/1449 nt) | *cusS* ← | sensory histidine kinase in two‑component regulatory system with CusR, senses copper ions |
| 588,070 | Δ1 bp | intergenic (‑222/‑47) | *ybdK* ← / → *insJ‑1* | gamma‑glutamyl:cysteine ligase/IS150 hypothetical protein |
| 1,457,389 | Δ11,725 bp | between IS*150* | *hrpA*–*insJ‑2* | *hrpA*, *ydcF*, *aldA*, *gapC*, *insA‑12*, *insB‑12*, *cybB*, *ydcA*, *hokB*, *mokB*, *insK‑2*, *insJ‑2* |
| 1,887,034 | IS*150* (+) +3 bp | intergenic (‑3/‑154) | *yobG* ← / → *ECB\_01797* | hypothetical protein/hypothetical protein |
| 1,988,168 | IS*150* (–) +3 bp | coding (5620‑5622/7152 nt) | *yeeJ* → | adhesin |
| 2,062,617 | Δ374 bp | IS*150*‑mediated | *yegL* ← / ← *insK‑2* | hypothetical protein/IS150 putative transposase |
| 2,099,889 | IS*150* (–) +3 bp | coding (991‑993/2280 nt) | *yehM* → | hypothetical protein |
| 2,659,020 | IS*3* (–) +3 bp :: +TCA | intergenic (‑212/+21) | *csrA* ← / ← *alaS* | carbon storage regulator/alanyl‑tRNA synthetase |
| 2,896,927 | IS*150* (+) +3 bp | coding (126‑128/1479 nt) | *ygfH* → | propionyl‑CoA:succinate‑CoA transferase |
| position | mutation | annotation | gene | description |
| 2,976,836 | IS*150* (–) +3 bp | intergenic (‑322/+31) | *yghK* ← / ← *glcB* | glycolate transporter/malate synthase |
| 3,109,394 | IS*150* (–) +3 bp | coding (245‑247/663 nt) | *yqjA* → | conserved inner membrane protein |
| 4,091,159 | C→T | R974C (CGT→TGT) | *rpoB* → | DNA‑directed RNA polymerase subunit beta |
| 4,122,888 | IS*150* (–) +3 bp | coding (582‑584/1602 nt) | *aceB* → | malate synthase |
