# Supplementary material for: Genomic and phenotypic evolution of Escherichia coli in a novel citrate-only resource environment
Source: eLife. 2020 May 29;9:e55414. doi: 10.7554/eLife.55414 (PMC7299349; doi:10.7554/eLife.55414)
Supplement: Supplementary file 5. [file elife-55414-supp5.zip › S4File_genomes-by-environment/DM25-html/ZDBp917_minus_ZDB67.html]

Mutation Comparison


| Predicted mutations | | | | |
| --- | --- | --- | --- | --- |
| position | mutation | annotation | gene | description |
| 549,926 | Δ39,972 bp | between IS*1* | *ECB\_00510*–*insA‑7* | **35 genes***ECB\_00510*, *nohB*, *ECB\_00512*, *ECB\_00513*, *ECB\_00514*, *ECB\_00515*, *ECB\_00516*, *ECB\_00517*, *appY*, *ompT*, *envY*, *ybcH*, *nfrA*, *ECB\_00524*, *yhhI*, *ECB\_00526*, *ECB\_00527*, *ECB\_00528*, *ECB\_00529*, *ECB\_00530*, *cusS*, *cusR*, *cusC*, *ylcC*, *cusB*, *cusA*, *pheP*, *ybdG*, *nfnB*, *ybdF*, *ybdJ*, *ybdK*, *insJ‑1*, *insB‑7*, *insA‑7* *ECB\_00510*, *nohB*, *ECB\_00512*, *ECB\_00513*, *ECB\_00514*, *ECB\_00515*, *ECB\_00516*, *ECB\_00517*, *appY*, *ompT*, *envY*, *ybcH*, *nfrA*, *ECB\_00524*, *yhhI*, *ECB\_00526*, *ECB\_00527*, *ECB\_00528*, *ECB\_00529*, *ECB\_00530*, *cusS*, *cusR*, *cusC*, *ylcC*, *cusB*, *cusA*, *pheP*, *ybdG*, *nfnB*, *ybdF*, *ybdJ*, *ybdK*, *insJ‑1*, *insB‑7*, *insA‑7* |
| 665,705 | Δ1 bp | intergenic (‑486/‑51) | *rihA* ← / → *insJ‑2* | ribonucleoside hydrolase 1/IS150 hypothetical protein |
| 736,739 | G→T | intergenic (‑459/‑250) | *gltA* ← / → *sdhC* | citrate synthase/succinate dehydrogenase cytochrome b556 large membrane subunit |
| 1,455,172 | Δ774 bp | IS*150*‑mediated | *[ynbD]* | *[ynbD]* |
| 1,915,305 | (A)8→7 | coding (391/870 nt) | *yebK* → | predicted DNA‑binding transcriptional regulator |
| 2,600,587 | +T | intergenic (‑51/+655) | *insJ‑2* ← / ← *rluD* | IS150 hypothetical protein/23S rRNA pseudouridine synthase |
| 3,398,568 | IS*150* (+) +3 bp | intergenic (+395/‑41) | *yhgF* → / → *feoA* | predicted transcriptional accessory protein/ferrous iron transport protein A |
| 4,091,094 | A→G | Q952R (CAG→CGG) | *rpoB* → | DNA‑directed RNA polymerase subunit beta |
| 4,122,957 | IS*150* (–) +4 bp | coding (651‑654/1602 nt) | *aceB* → | malate synthase |
| 4,134,124 | C→T | R27C (CGC→TGC) | *yjbB* → | predicted transporter |
| position | mutation | annotation | gene | description |
| 4,502,903 | +A | intergenic (‑16/‑50) | *smp* ← / → *insJ‑2* | hypothetical protein/IS150 hypothetical protein |
