# Supplementary material for: Genomic and phenotypic evolution of Escherichia coli in a novel citrate-only resource environment
Source: eLife. 2020 May 29;9:e55414. doi: 10.7554/eLife.55414 (PMC7299349; doi:10.7554/eLife.55414)
Supplement: Supplementary file 5. [file elife-55414-supp5.zip › S4File_genomes-by-environment/DM25-html/ZDBp915_minus_CZB154.html]

Mutation Comparison


| Predicted mutations | | | | |
| --- | --- | --- | --- | --- |
| position | mutation | annotation | gene | description |
| 319,673 | IS*150* (+) +3 bp | coding (174‑176/246 nt) | *yahM* → | hypothetical protein |
| 492,684 | C→T | G28D (GGC→GAC) | *ybbO* ← | short chain dehydrogenase |
| 581,931 | Δ6,139 bp | IS*150*‑mediated | *[cusA]*–*ybdK* | *[cusA]*, *pheP*, *ybdG*, *nfnB*, *ybdF*, *ybdJ*, *ybdK* |
| 600,001 | IS*150* (–) +3 bp | coding (3562‑3564/3882 nt) | *entF* → | enterobactin synthase multienzyme complex component, ATP‑dependent |
| 642,823 | Δ117 bp | IS*150*‑mediated | *lipA* ← / → *insJ‑2* | lipoyl synthase/IS150 hypothetical protein |
| 665,708 | Δ2 bp | intergenic (‑489/‑47) | *rihA* ← / → *insJ‑2* | ribonucleoside hydrolase 1/IS150 hypothetical protein |
| 734,998 | IS*1* (–) +9 bp | coding (1275‑1283/1284 nt) | *gltA* ← | citrate synthase |
| 1,107,553 | IS*150* (+) +3 bp | coding (2041‑2043/2424 nt) | *ycdS* ← | predicted outer membrane protein |
| 1,173,387 | IS*150* (+) +3 bp | intergenic (+223/‑70) | *ycfH* → / → *ptsG* | predicted metallodependent hydrolase/fused glucose‑specific PTS enzymes: IIB component/IIC component |
| 1,447,820 | A→C | K285Q (AAG→CAG) | *insF‑2* → | IS3 element protein InsF |
| position | mutation | annotation | gene | description |
| 1,457,389 | Δ11,725 bp | between IS*150* | *hrpA*–*insJ‑2* | *hrpA*, *ydcF*, *aldA*, *gapC*, *insA‑12*, *insB‑12*, *cybB*, *ydcA*, *hokB*, *mokB*, *insK‑2*, *insJ‑2* |
| 1,534,643 | G→A | intergenic (‑73/+61) | *adhP* ← / ← *sfcA* | alcohol dehydrogenase/malate dehydrogenase, (decarboxylating, NAD‑requiring) (malic enzyme) |
| 2,264,348 | IS*186* (–) +8 bp | coding (129‑136/963 nt) | *menC* ← | O‑succinylbenzoate synthase |
| 2,600,587 | +T | intergenic (‑51/+655) | *insJ‑2* ← / ← *rluD* | IS150 hypothetical protein/23S rRNA pseudouridine synthase |
| 2,820,985 | IS*150* (–) +3 bp | coding (620‑622/1419 nt) | *araE* ← | arabinose transporter |
| 3,109,394 | IS*150* (–) +4 bp | coding (245‑248/663 nt) | *yqjA* → | conserved inner membrane protein |
| 3,459,518 | (T)5→4 | intergenic (‑152/‑272) | *livK* ← / → *yhhK* | leucine transporter subunit/hypothetical protein |
| 3,504,875 | T→A | H27L (CAC→CTC) | *yhiQ* ← | predicted SAM‑dependent methyltransferase |
| 3,857,836 | IS*150* (+) +3 bp | intergenic (+21/‑33) | *wzzE* → / → *wecB* | Entobacterial Common Antigen (ECA) polysaccharide chain length modulation protein/UDP‑N‑acetyl glucosamine‑2‑epimerase |
| 3,993,788 | IS*150* (+) +3 bp | coding (151‑153/1749 nt) | *frvR* ← | predicted regulator |
| position | mutation | annotation | gene | description |
| 4,478,024 | IS*150* (–) +3 bp | coding (534‑536/2292 nt) | *mdoB* ← | phosphoglycerol transferase I |
