# Supplementary material for: Genomic and phenotypic evolution of Escherichia coli in a novel citrate-only resource environment
Source: eLife. 2020 May 29;9:e55414. doi: 10.7554/eLife.55414 (PMC7299349; doi:10.7554/eLife.55414)
Supplement: Supplementary file 5. [file elife-55414-supp5.zip › S4File_genomes-by-environment/DM25-html/ZDBp912_minus_CZB152.html]

Mutation Comparison


| Predicted mutations | | | | |
| --- | --- | --- | --- | --- |
| position | mutation | annotation | gene | description |
| 435,258 | IS*150* (+) Δ1 bp :: +TTC | intergenic (+33/‑159) | *hupB* → / → *ppiD* | HU, DNA‑binding transcriptional regulator, beta subunit/peptidyl‑prolyl cis‑trans isomerase (rotamase D) |
| 492,501 | IS*150* (–) +3 bp | coding (264‑266/810 nt) | *ybbO* ← | short chain dehydrogenase |
| 544,593 | +TGA :: IS*3* (+) +3 bp | coding (362‑364/552 nt) | *ybcL* → | predicted kinase inhibitor |
| 665,708 | Δ2 bp | intergenic (‑489/‑47) | *rihA* ← / → *insJ‑2* | ribonucleoside hydrolase 1/IS150 hypothetical protein |
| 1,137,051 | IS*150* (+) +3 bp | coding (28‑30/246 nt) | *dinI* ← | DNA damage‑inducible protein I |
| 1,271,135 | Δ15 bp | IS*150*‑mediated | *ldrB* ← / ← *insK‑2* | toxic polypeptide, small/IS150 putative transposase |
| 2,264,346 | IS*186* (+) +8 bp | coding (131‑138/963 nt) | *menC* ← | O‑succinylbenzoate synthase |
| 2,264,348 | IS*186* (–) +8 bp | coding (129‑136/963 nt) | *menC* ← | O‑succinylbenzoate synthase |
| 2,553,002 | IS*150* (–) +3 bp | coding (560‑562/714 nt) | *yfhG* ← | hypothetical protein |
| 2,812,156 | IS*150* (–) +3 bp | coding (1128‑1130/1194 nt) | *ygeD* ← | predicted inner membrane protein |
| position | mutation | annotation | gene | description |
| 2,896,913 | IS*150* (+) +3 bp | coding (112‑114/1479 nt) | *ygfH* → | propionyl‑CoA:succinate‑CoA transferase |
| 2,978,663 | IS*150* (+) +3 bp | coding (376‑378/2172 nt) | *glcB* ← | malate synthase |
| 3,362,135 | C→T | C132C (TGC→TGT) | *yhfR* → | predicted DNA‑binding transcriptional regulator |
| 3,466,937 | IS*150* (+) +4 bp | coding (139‑142/270 nt) | *yhhL* → | conserved inner membrane protein |
| 3,501,576 | IS*150* (–) +3 bp | intergenic (‑35/‑354) | *yhiO* ← / → *uspA* | universal stress protein UspB/universal stress global response regulator |
| 4,022,848 | Δ7 bp | coding (657‑663/846 nt) | *glpF* ← | glycerol facilitator |
| 4,095,055 | C→T | A904V (GCG→GTG) | *rpoC* → | DNA‑directed RNA polymerase subunit beta' |
| 4,342,160 | +T | intergenic (+222/+963) | *fklB* → / ← *insK‑2* | FKBP‑type peptidyl‑prolyl cis‑trans isomerase (rotamase)/IS150 putative transposase |
