# Supplementary material for: Genomic and phenotypic evolution of Escherichia coli in a novel citrate-only resource environment
Source: eLife. 2020 May 29;9:e55414. doi: 10.7554/eLife.55414 (PMC7299349; doi:10.7554/eLife.55414)
Supplement: Supplementary file 5. [file elife-55414-supp5.zip › S4File_genomes-by-environment/DM25-html/ZDBp921_minus_ZDB69.html]

Mutation Comparison


| Predicted mutations | | | | |
| --- | --- | --- | --- | --- |
| position | mutation | annotation | gene | description |
| 432,291 | 3 bp→ATT | intergenic (‑58/‑96) | *insL‑2* ← / → *lon* | putative transposase insL for insertion sequence IS186/DNA‑binding ATP‑dependent protease La |
| 432,296 | Δ1 bp | intergenic (‑63/‑93) | *insL‑2* ← / → *lon* | putative transposase insL for insertion sequence IS186/DNA‑binding ATP‑dependent protease La |
| 432,301 | Δ1 bp | intergenic (‑68/‑88) | *insL‑2* ← / → *lon* | putative transposase insL for insertion sequence IS186/DNA‑binding ATP‑dependent protease La |
| 464,051 | IS*150* (+) +3 bp | coding (274‑276/528 nt) | *priC* ← | primosomal replication protein N'' |
| 546,183 | Δ42,947 bp | IS*1*‑mediated | *[ybcN]*–*insJ‑1* | **42 genes***[ybcN]*, *ninE*, *ybcO*, *rus*, *ylcG*, *insA‑10*, *insB‑10*, *insB‑6*, *insA‑6*, *ECB\_00510*, *nohB*, *ECB\_00512*, *ECB\_00513*, *ECB\_00514*, *ECB\_00515*, *ECB\_00516*, *ECB\_00517*, *appY*, *ompT*, *envY*, *ybcH*, *nfrA*, *ECB\_00524*, *yhhI*, *ECB\_00526*, *ECB\_00527*, *ECB\_00528*, *ECB\_00529*, *ECB\_00530*, *cusS*, *cusR*, *cusC*, *ylcC*, *cusB*, *cusA*, *pheP*, *ybdG*, *nfnB*, *ybdF*, *ybdJ*, *ybdK*, *insJ‑1* *[ybcN]*, *ninE*, *ybcO*, *rus*, *ylcG*, *insA‑10*, *insB‑10*, *insB‑6*, *insA‑6*, *ECB\_00510*, *nohB*, *ECB\_00512*, *ECB\_00513*, *ECB\_00514*, *ECB\_00515*, *ECB\_00516*, *ECB\_00517*, *appY*, *ompT*, *envY*, *ybcH*, *nfrA*, *ECB\_00524*, *yhhI*, *ECB\_00526*, *ECB\_00527*, *ECB\_00528*, *ECB\_00529*, *ECB\_00530*, *cusS*, *cusR*, *cusC*, *ylcC*, *cusB*, *cusA*, *pheP*, *ybdG*, *nfnB*, *ybdF*, *ybdJ*, *ybdK*, *insJ‑1* |
| 736,633 | (GTTGA)1→2 | intergenic (‑353/‑356) | *gltA* ← / → *sdhC* | citrate synthase/succinate dehydrogenase cytochrome b556 large membrane subunit |
| 1,137,052 | IS*150* (+) +3 bp | coding (27‑29/246 nt) | *dinI* ← | DNA damage‑inducible protein I |
| 1,457,392 | Δ1 bp | intergenic (‑51/‑474) | *insJ‑2* ← / → *hrpA* | IS150 hypothetical protein/ATP‑dependent helicase |
| 2,112,669 | IS*150* (+) +3 bp | coding (16‑18/918 nt) | *yehZ* ← | predicted transporter subunit: periplasmic‑binding component of ABC superfamily |
| 2,250,174 | C→T | G187S (GGC→AGC) | *yfaX* ← | predicted DNA‑binding transcriptional regulator |
| position | mutation | annotation | gene | description |
| 3,109,394 | IS*150* (–) +3 bp | coding (245‑247/663 nt) | *yqjA* → | conserved inner membrane protein |
| 4,101,561 | IS*150* (–) +3 bp | coding (1532‑1534/1896 nt) | *thiC* ← | thiamine biosynthesis protein ThiC |
| 4,124,014 | Δ2 bp | coding (77‑78/1305 nt) | *aceA* → | isocitrate lyase |
| 4,256,901 | (CGCGG)3→2 | intergenic (‑768/‑1042) | *dcuR* ← / → *yjdI* | DNA‑binding response regulator in two‑component regulatory system with DcuS/hypothetical protein |
| 4,403,966 | IS*150* (+) +3 bp | coding (613‑615/1320 nt) | *idnT* ← | L‑idonate and D‑gluconate transporter |
