# Supplementary material for: Genomic and phenotypic evolution of Escherichia coli in a novel citrate-only resource environment
Source: eLife. 2020 May 29;9:e55414. doi: 10.7554/eLife.55414 (PMC7299349; doi:10.7554/eLife.55414)
Supplement: Supplementary file 5. [file elife-55414-supp5.zip › S4File_genomes-by-environment/DM25-html/ZDBp916_minus_ZDB67.html]

Mutation Comparison


| Predicted mutations | | | | |
| --- | --- | --- | --- | --- |
| position | mutation | annotation | gene | description |
| 549,926 | Δ39,972 bp | between IS*1* | *ECB\_00510*–*insA‑7* | **35 genes***ECB\_00510*, *nohB*, *ECB\_00512*, *ECB\_00513*, *ECB\_00514*, *ECB\_00515*, *ECB\_00516*, *ECB\_00517*, *appY*, *ompT*, *envY*, *ybcH*, *nfrA*, *ECB\_00524*, *yhhI*, *ECB\_00526*, *ECB\_00527*, *ECB\_00528*, *ECB\_00529*, *ECB\_00530*, *cusS*, *cusR*, *cusC*, *ylcC*, *cusB*, *cusA*, *pheP*, *ybdG*, *nfnB*, *ybdF*, *ybdJ*, *ybdK*, *insJ‑1*, *insB‑7*, *insA‑7* *ECB\_00510*, *nohB*, *ECB\_00512*, *ECB\_00513*, *ECB\_00514*, *ECB\_00515*, *ECB\_00516*, *ECB\_00517*, *appY*, *ompT*, *envY*, *ybcH*, *nfrA*, *ECB\_00524*, *yhhI*, *ECB\_00526*, *ECB\_00527*, *ECB\_00528*, *ECB\_00529*, *ECB\_00530*, *cusS*, *cusR*, *cusC*, *ylcC*, *cusB*, *cusA*, *pheP*, *ybdG*, *nfnB*, *ybdF*, *ybdJ*, *ybdK*, *insJ‑1*, *insB‑7*, *insA‑7* |
| 736,735 | T→C | intergenic (‑455/‑254) | *gltA* ← / → *sdhC* | citrate synthase/succinate dehydrogenase cytochrome b556 large membrane subunit |
| 1,270,529 | Δ621 bp | IS*150*‑mediated | *ldrB* | ldrB |
| 1,457,389 | Δ11,725 bp | between IS*150* | *hrpA*–*insJ‑2* | *hrpA*, *ydcF*, *aldA*, *gapC*, *insA‑12*, *insB‑12*, *cybB*, *ydcA*, *hokB*, *mokB*, *insK‑2*, *insJ‑2* |
| 1,729,739 | +G | intergenic (‑50/+700) | *insJ‑2* ← / ← *ydhZ* | IS150 hypothetical protein/hypothetical protein |
| 2,133,568 | IS*150* (+) +3 bp | coding (25‑27/999 nt) | *mglB* ← | methyl‑galactoside transporter subunit |
| 2,694,867 | +T :: IS*150* (+) +3 bp | coding (867‑869/2079 nt) | *fhlA* → | DNA‑binding transcriptional activator |
| 2,844,380 | Δ41 bp | IS*150*‑mediated | *yqeA* → / ← *insK‑2* | predicted amino acid kinase/IS150 putative transposase |
| 3,391,209 | A→G | intergenic (‑303/‑76) | *yhgE* ← / → *pckA* | predicted inner membrane protein/phosphoenolpyruvate carboxykinase |
| 3,501,576 | IS*150* (+) +3 bp | intergenic (‑35/‑354) | *yhiO* ← / → *uspA* | universal stress protein UspB/universal stress global response regulator |
| position | mutation | annotation | gene | description |
| 4,123,908 | IS*1* (+) +9 bp |  | *[aceB]* | *[aceB]* |
| 4,191,234 | IS*150* (+) +3 bp | coding (295‑297/315 nt) | *yjcH* ← | conserved inner membrane protein involved in acetate transport |
