# Supplementary material for: Genomic and phenotypic evolution of Escherichia coli in a novel citrate-only resource environment
Source: eLife. 2020 May 29;9:e55414. doi: 10.7554/eLife.55414 (PMC7299349; doi:10.7554/eLife.55414)
Supplement: Supplementary file 5. [file elife-55414-supp5.zip › S4File_genomes-by-environment/DM25-html/ZDBp920_minus_ZDB69.html]

Mutation Comparison


| Predicted mutations | | | | |
| --- | --- | --- | --- | --- |
| position | mutation | annotation | gene | description |
| 464,051 | IS*150* (+) +3 bp | coding (274‑276/528 nt) | *priC* ← | primosomal replication protein N'' |
| 549,926 | Δ39,972 bp | between IS*1* | *ECB\_00510*–*insA‑7* | **35 genes***ECB\_00510*, *nohB*, *ECB\_00512*, *ECB\_00513*, *ECB\_00514*, *ECB\_00515*, *ECB\_00516*, *ECB\_00517*, *appY*, *ompT*, *envY*, *ybcH*, *nfrA*, *ECB\_00524*, *yhhI*, *ECB\_00526*, *ECB\_00527*, *ECB\_00528*, *ECB\_00529*, *ECB\_00530*, *cusS*, *cusR*, *cusC*, *ylcC*, *cusB*, *cusA*, *pheP*, *ybdG*, *nfnB*, *ybdF*, *ybdJ*, *ybdK*, *insJ‑1*, *insB‑7*, *insA‑7* *ECB\_00510*, *nohB*, *ECB\_00512*, *ECB\_00513*, *ECB\_00514*, *ECB\_00515*, *ECB\_00516*, *ECB\_00517*, *appY*, *ompT*, *envY*, *ybcH*, *nfrA*, *ECB\_00524*, *yhhI*, *ECB\_00526*, *ECB\_00527*, *ECB\_00528*, *ECB\_00529*, *ECB\_00530*, *cusS*, *cusR*, *cusC*, *ylcC*, *cusB*, *cusA*, *pheP*, *ybdG*, *nfnB*, *ybdF*, *ybdJ*, *ybdK*, *insJ‑1*, *insB‑7*, *insA‑7* |
| 642,939 | Δ1 bp | intergenic (‑565/‑48) | *lipA* ← / → *insJ‑2* | lipoyl synthase/IS150 hypothetical protein |
| 665,708 | Δ2 bp | intergenic (‑489/‑47) | *rihA* ← / → *insJ‑2* | ribonucleoside hydrolase 1/IS150 hypothetical protein |
| 735,392 | C→A | D297Y (GAT→TAT) | *gltA* ← | citrate synthase |
| 889,395 | G→T | intergenic (+68/‑85) | *ECB\_00825* → / → *ECB\_00826* | putative replication protein for prophage/conserved hypothetical protein |
| 1,137,050 | IS*150* (–) +3 bp | coding (29‑31/246 nt) | *dinI* ← | DNA damage‑inducible protein I |
| 1,457,389 | Δ11,725 bp | between IS*150* | *hrpA*–*insJ‑2* | *hrpA*, *ydcF*, *aldA*, *gapC*, *insA‑12*, *insB‑12*, *cybB*, *ydcA*, *hokB*, *mokB*, *insK‑2*, *insJ‑2* |
| 1,729,741 | Δ1 bp | intergenic (‑52/+698) | *insJ‑2* ← / ← *ydhZ* | IS150 hypothetical protein/hypothetical protein |
| 2,062,617 | Δ374 bp | IS*150*‑mediated | *yegL* ← / ← *insK‑2* | hypothetical protein/IS150 putative transposase |
| position | mutation | annotation | gene | description |
| 2,544,924 | C→T | E627K (GAG→AAG) | *yphG* ← | hypothetical protein |
| 2,942,822 | Δ19,201 bp | IS*150*‑mediated | *[ECB\_02797]*–*ECB\_02815* | **18 genes***[ECB\_02797]*, *ECB\_02798*, *flu*, *yeeR*, *ECB\_02802*, *yafZ*, *ECB\_02804*, *yeeS*, *yeeT*, *yeeU*, *yeeV*, *yeeW*, *ECB\_02810*, *ECB\_02811*, *ECB\_02812*, *ECB\_02813*, *ECB\_02814*, *ECB\_02815* *[ECB\_02797]*, *ECB\_02798*, *flu*, *yeeR*, *ECB\_02802*, *yafZ*, *ECB\_02804*, *yeeS*, *yeeT*, *yeeU*, *yeeV*, *yeeW*, *ECB\_02810*, *ECB\_02811*, *ECB\_02812*, *ECB\_02813*, *ECB\_02814*, *ECB\_02815* |
| 3,109,394 | IS*150* (–) +3 bp | coding (245‑247/663 nt) | *yqjA* → | conserved inner membrane protein |
| 3,501,576 | IS*150* (+) +3 bp | intergenic (‑35/‑354) | *yhiO* ← / → *uspA* | universal stress protein UspB/universal stress global response regulator |
| 3,770,422 | C→T | intergenic (‑195/+45) | *yidB* ← / ← *gyrB* | hypothetical protein/DNA gyrase subunit B |
| 4,122,799 | Δ1 bp | coding (493/1602 nt) | *aceB* → | malate synthase |
| 4,256,901 | (CGCGG)3→2 | intergenic (‑768/‑1042) | *dcuR* ← / → *yjdI* | DNA‑binding response regulator in two‑component regulatory system with DcuS/hypothetical protein |
