# Supplementary material for: Genomic and phenotypic evolution of Escherichia coli in a novel citrate-only resource environment
Source: eLife. 2020 May 29;9:e55414. doi: 10.7554/eLife.55414 (PMC7299349; doi:10.7554/eLife.55414)
Supplement: Supplementary file 5. [file elife-55414-supp5.zip › S4File_genomes-by-environment/DM0-genome-summary.html]

Mutation Comparison


| Predicted mutations | | | | | | | | | | | | | | | | |
| --- | --- | --- | --- | --- | --- | --- | --- | --- | --- | --- | --- | --- | --- | --- | --- | --- |
| position | mutation | ZDBp871\_minus\_CZB151 | ZDBp875\_minus\_CZB151 | ZDBp877\_minus\_CZB152 | ZDBp880\_minus\_CZB152 | ZDBp883\_minus\_CZB154 | ZDBp886\_minus\_CZB154 | ZDBp889\_minus\_ZDB67 | ZDBp892\_minus\_ZDB67 | ZDBp895\_minus\_ZDB68 | ZDBp898\_minus\_ZDB68 | ZDBp901\_minus\_ZDB69 | ZDBp904\_minus\_ZDB69 | annotation | gene | description |
| 140,966 | IS*150* (–) +4 bp |  |  |  |  |  |  |  |  |  | 100% |  |  | coding (198‑201/348 nt) | *yacC* ← | hypothetical protein |
| 199,024 | IS*150* (+) +3 bp | 100% |  |  |  |  |  |  |  |  |  |  |  | intergenic (+19/‑165) | *ispC* → / → *uppS* | 1‑deoxy‑D‑xylulose 5‑phosphate reductoisomerase/undecaprenyl pyrophosphate synthase |
| 237,985 | IS*150* (+) +3 bp |  |  |  |  |  |  |  |  |  |  |  | 100% | coding (339‑341/1359 nt) | *mltD* ← | predicted membrane‑bound lytic murein transglycosylase D |
| 243,649 | (G)8→7 |  |  |  |  |  | 100% |  |  |  |  |  |  | coding (193/372 nt) | *ECB\_00212* → | hypothetical protein |
| 247,779 | IS*150* (–) +3 bp |  |  |  |  | 100% |  |  |  |  |  |  |  | coding (1540‑1542/2445 nt) | *fadE* ← | acyl‑CoA dehydrogenase |
| 248,532 | IS*150* (–) +4 bp |  |  |  |  |  |  |  |  | 100% |  |  |  | coding (786‑789/2445 nt) | *fadE* ← | acyl‑CoA dehydrogenase |
| 249,356 | C→A |  |  |  |  |  |  |  |  |  |  | 100% |  | intergenic (‑36/‑204) | *fadE* ← / → *lpcA* | acyl‑CoA dehydrogenase/phosphoheptose isomerase |
| 323,378 | G→A |  |  | 100% |  |  |  |  |  |  |  |  |  | D201N (GAT→AAT) | *prpB* → | 2‑methylisocitrate lyase |
| 435,255 | IS*150* (+) +3 bp |  |  |  |  |  |  | 100% |  |  |  |  |  | intergenic (+30/‑160) | *hupB* → / → *ppiD* | HU, DNA‑binding transcriptional regulator, beta subunit/peptidyl‑prolyl cis‑trans isomerase (rotamase D) |
| 464,051 | IS*150* (+) +3 bp |  |  |  |  |  |  |  | 100% |  |  | 100% |  | coding (274‑276/528 nt) | *priC* ← | primosomal replication protein N'' |
| position | mutation | ZDBp871\_minus\_CZB151 | ZDBp875\_minus\_CZB151 | ZDBp877\_minus\_CZB152 | ZDBp880\_minus\_CZB152 | ZDBp883\_minus\_CZB154 | ZDBp886\_minus\_CZB154 | ZDBp889\_minus\_ZDB67 | ZDBp892\_minus\_ZDB67 | ZDBp895\_minus\_ZDB68 | ZDBp898\_minus\_ZDB68 | ZDBp901\_minus\_ZDB69 | ZDBp904\_minus\_ZDB69 | annotation | gene | description |
| 468,912 | IS*150* (+) +3 bp |  |  |  |  |  |  |  |  |  | 100% |  |  | coding (279‑281/1875 nt) | *htpG* → | heat shock protein 90 |
| 547,546 | Δ103 bp | 100% |  |  |  |  |  |  |  |  |  |  |  | IS*1*‑mediated | *ylcG* → / → *insA‑10* | predicted protein/IS1 protein InsA |
| 549,926 | Δ39,972 bp | 100% |  |  | 100% |  |  |  |  |  |  |  | ? | between IS*1* | *ECB\_00510*–*insA‑7* | **35 genes***ECB\_00510*, *nohB*, *ECB\_00512*, *ECB\_00513*, *ECB\_00514*, *ECB\_00515*, *ECB\_00516*, *ECB\_00517*, *appY*, *ompT*, *envY*, *ybcH*, *nfrA*, *ECB\_00524*, *yhhI*, *ECB\_00526*, *ECB\_00527*, *ECB\_00528*, *ECB\_00529*, *ECB\_00530*, *cusS*, *cusR*, *cusC*, *ylcC*, *cusB*, *cusA*, *pheP*, *ybdG*, *nfnB*, *ybdF*, *ybdJ*, *ybdK*, *insJ‑1*, *insB‑7*, *insA‑7* *ECB\_00510*, *nohB*, *ECB\_00512*, *ECB\_00513*, *ECB\_00514*, *ECB\_00515*, *ECB\_00516*, *ECB\_00517*, *appY*, *ompT*, *envY*, *ybcH*, *nfrA*, *ECB\_00524*, *yhhI*, *ECB\_00526*, *ECB\_00527*, *ECB\_00528*, *ECB\_00529*, *ECB\_00530*, *cusS*, *cusR*, *cusC*, *ylcC*, *cusB*, *cusA*, *pheP*, *ybdG*, *nfnB*, *ybdF*, *ybdJ*, *ybdK*, *insJ‑1*, *insB‑7*, *insA‑7* |
| 557,465 | +TGA :: IS*3* (+) +3 bp | Δ |  | 100% | Δ |  |  |  |  |  |  |  | ? | coding (542‑544/705 nt) | *ECB\_00515* → | conserved hypothetical protein |
| 572,890 | Δ16,240 bp | Δ |  |  | Δ |  | 100% |  |  |  |  | ? | ? | IS*1*‑mediated | *[ECB\_00530]*–*insJ‑1* | *[ECB\_00530]*, *cusS*, *cusR*, *cusC*, *ylcC*, *cusB*, *cusA*, *pheP*, *ybdG*, *nfnB*, *ybdF*, *ybdJ*, *ybdK*, *insJ‑1* |
| 574,068 | IS*150* (–) +3 bp | Δ | 100% |  | Δ |  | Δ | ? | 100% |  |  | ? | ? | coding (1179‑1181/1449 nt) | *cusS* ← | sensory histidine kinase in two‑component regulatory system with CusR, senses copper ions |
| 579,159 | IS*150* (–) +3 bp | Δ |  | ? | Δ | 100% | Δ |  |  |  |  | ? | ? | coding (1203‑1205/1224 nt) | *cusB* → | copper/silver efflux system, membrane fusion protein |
| 590,047 | Δ2,134 bp |  | Δ | ? | ? | Δ |  | 100% |  | ? | ? | ? | ? | IS*150*‑mediated | *hokE*–*[entD]* | *hokE*, *insL‑3*, *[entD]* |
| 590,047 | Δ2,297 bp |  | 100% | ? | ? | Δ |  |  |  | ? | ? | ? | ? | IS*150*‑mediated | *hokE*–*[entD]* | *hokE*, *insL‑3*, *[entD]* |
| 590,047 | Δ8,054 bp |  |  |  |  | 100% |  |  |  |  |  | ? | ? | IS*150*‑mediated | *hokE*–*[entF]* | *hokE*, *insL‑3*, *entD*, *fepA*, *fes*, *ybdZ*, *[entF]* |
| position | mutation | ZDBp871\_minus\_CZB151 | ZDBp875\_minus\_CZB151 | ZDBp877\_minus\_CZB152 | ZDBp880\_minus\_CZB152 | ZDBp883\_minus\_CZB154 | ZDBp886\_minus\_CZB154 | ZDBp889\_minus\_ZDB67 | ZDBp892\_minus\_ZDB67 | ZDBp895\_minus\_ZDB68 | ZDBp898\_minus\_ZDB68 | ZDBp901\_minus\_ZDB69 | ZDBp904\_minus\_ZDB69 | annotation | gene | description |
| 599,136 | IS*RSO11* (+) +3 bp | 100% |  |  |  |  |  |  |  |  |  | ? | ? | coding (2697‑2699/3882 nt) | *entF* → | enterobactin synthase multienzyme complex component, ATP‑dependent |
| 642,809 | Δ131 bp |  |  |  |  |  |  |  |  |  | 100% |  |  | IS*150*‑mediated | *lipA* ← / → *insJ‑2* | lipoyl synthase/IS150 hypothetical protein |
| 642,935 | Δ1 bp |  |  |  | 100% |  |  |  |  |  | Δ |  |  | intergenic (‑561/‑52) | *lipA* ← / → *insJ‑2* | lipoyl synthase/IS150 hypothetical protein |
| 660,274 | Δ5,435 bp |  |  |  |  |  |  |  | 100% |  |  |  |  | IS*150*‑mediated | *ybeR*–*rihA* | *ybeR*, *ybeV*, *hscC*, *rihA* |
| 681,779 | IS*150* (+) +3 bp |  |  |  |  |  |  |  |  |  |  | 100% |  | coding (513‑515/1665 nt) | *asnB* ← | asparagine synthetase B |
| 719,392 | 2 bp→CA |  |  |  |  | 100% |  |  |  |  |  |  |  | intergenic (+986/‑613) | *ECB\_00664* → / → *ybfD* | hypothetical protein/hypothetical protein |
| 719,413 | G→T |  |  |  |  | 100% |  |  |  |  |  |  |  | intergenic (+1007/‑593) | *ECB\_00664* → / → *ybfD* | hypothetical protein/hypothetical protein |
| 734,968 | IS*1* (–) +9 bp |  |  |  |  |  |  |  |  |  |  |  | 100% | intergenic (‑361/+21) | *ybgD* ← / ← *gltA* | predicted fimbrial‑like adhesin protein/citrate synthase |
| 735,104 | T→G |  |  |  |  | 100% |  |  |  |  |  |  |  | I393L (ATC→CTC) | *gltA* ← | citrate synthase |
| 735,572 | A→C |  |  |  |  |  |  |  |  |  |  | 100% |  | S237A (TCC→GCC) | *gltA* ← | citrate synthase |
| position | mutation | ZDBp871\_minus\_CZB151 | ZDBp875\_minus\_CZB151 | ZDBp877\_minus\_CZB152 | ZDBp880\_minus\_CZB152 | ZDBp883\_minus\_CZB154 | ZDBp886\_minus\_CZB154 | ZDBp889\_minus\_ZDB67 | ZDBp892\_minus\_ZDB67 | ZDBp895\_minus\_ZDB68 | ZDBp898\_minus\_ZDB68 | ZDBp901\_minus\_ZDB69 | ZDBp904\_minus\_ZDB69 | annotation | gene | description |
| 735,580 | G→T |  | 100% |  |  |  |  |  |  |  |  |  |  | A234D (GCC→GAC) | *gltA* ← | citrate synthase |
| 735,765 | C→A |  |  | 100% | 100% |  |  |  |  |  |  |  |  | M172I (ATG→ATT) | *gltA* ← | citrate synthase |
| 735,797 | C→T |  |  |  |  |  |  |  |  | 100% | 100% |  |  | A162T (GCG→ACG) | *gltA* ← | citrate synthase |
| 735,941 | T→A |  |  |  |  |  |  | 100% | 100% |  |  |  |  | I114F (ATC→TTC) | *gltA* ← | citrate synthase |
| 736,121 | C→T |  |  |  |  |  |  |  |  |  |  | 100% |  | E54K (GAA→AAA) | *gltA* ← | citrate synthase |
| 736,126 | G→T |  |  |  |  |  | 100% |  |  |  |  |  |  | S52Y (TCC→TAC) | *gltA* ← | citrate synthase |
| 736,323 | T→G | 100% |  |  |  |  |  |  |  |  |  |  |  | intergenic (‑43/‑666) | *gltA* ← / → *sdhC* | citrate synthase/succinate dehydrogenase cytochrome b556 large membrane subunit |
| 736,619 | T→C |  | 100% |  |  |  |  |  |  |  |  |  |  | intergenic (‑339/‑370) | *gltA* ← / → *sdhC* | citrate synthase/succinate dehydrogenase cytochrome b556 large membrane subunit |
| 831,886 | C→T |  |  |  |  |  |  |  |  |  |  |  | 100% | A73T (GCT→ACT) | *ybiI* ← | hypothetical protein |
| 891,825 | +TGA :: IS*3* (+) +3 bp |  |  |  |  |  |  |  |  | 100% |  |  |  | coding (913‑915/924 nt) | *ECB\_00830* → | hypothetical protein |
| position | mutation | ZDBp871\_minus\_CZB151 | ZDBp875\_minus\_CZB151 | ZDBp877\_minus\_CZB152 | ZDBp880\_minus\_CZB152 | ZDBp883\_minus\_CZB154 | ZDBp886\_minus\_CZB154 | ZDBp889\_minus\_ZDB67 | ZDBp892\_minus\_ZDB67 | ZDBp895\_minus\_ZDB68 | ZDBp898\_minus\_ZDB68 | ZDBp901\_minus\_ZDB69 | ZDBp904\_minus\_ZDB69 | annotation | gene | description |
| 923,149 | IS*150* (–) +3 bp |  | 100% |  |  |  |  |  |  |  |  |  |  | coding (509‑511/831 nt) | *ybjR* → | predicted amidase and lipoprotein |
| 923,167 | IS*150* (+) +3 bp |  |  |  |  |  |  |  |  |  |  | 100% |  | coding (527‑529/831 nt) | *ybjR* → | predicted amidase and lipoprotein |
| 939,393 | Δ3,661 bp |  |  |  |  |  |  | 100% |  |  |  |  |  |  | *[macB]*–*[clpA]* | *[macB]*, *cspD*, *clpS*, *[clpA]* |
| 940,328 | C→T |  |  | 100% |  |  |  | Δ |  |  |  |  |  | intergenic (‑11/‑312) | *cspD* ← / → *clpS* | cold shock protein homolog/ATP‑dependent Clp protease adaptor protein ClpS |
| 969,347 | IS*150* (–) +3 bp |  |  |  |  |  |  |  |  |  |  |  | 100% | intergenic (‑153/+37) | *pflA* ← / ← *pflB* | pyruvate formate lyase activating enzyme 1/pyruvate formate lyase I |
| 1,004,636 | G→A |  | 100% |  |  |  |  |  |  |  |  |  |  | R154C (CGT→TGT) | *ompF* ← | outer membrane porin 1a (Ia;b;F) |
| 1,004,674 | C→A |  |  | 100% |  |  |  |  |  |  |  |  |  | G141V (GGC→GTC) | *ompF* ← | outer membrane porin 1a (Ia;b;F) |
| 1,004,905 | C→T |  |  |  |  |  |  |  |  | 100% |  |  |  | R64H (CGT→CAT) | *ompF* ← | outer membrane porin 1a (Ia;b;F) |
| 1,039,280 | IS*150* (–) +3 bp |  |  |  |  |  |  | 100% |  |  |  |  |  | coding (31‑33/630 nt) | *yccR* → | hypothetical protein |
| 1,064,767 | IS*150* (–) +3 bp |  |  |  |  |  |  | 100% |  |  |  |  |  | coding (1289‑1291/2097 nt) | *ymcA* ← | hypothetical protein |
| position | mutation | ZDBp871\_minus\_CZB151 | ZDBp875\_minus\_CZB151 | ZDBp877\_minus\_CZB152 | ZDBp880\_minus\_CZB152 | ZDBp883\_minus\_CZB154 | ZDBp886\_minus\_CZB154 | ZDBp889\_minus\_ZDB67 | ZDBp892\_minus\_ZDB67 | ZDBp895\_minus\_ZDB68 | ZDBp898\_minus\_ZDB68 | ZDBp901\_minus\_ZDB69 | ZDBp904\_minus\_ZDB69 | annotation | gene | description |
| 1,097,283 | IS*150* (–) +3 bp |  |  |  |  |  |  |  |  |  | 100% |  |  | coding (674‑676/1509 nt) | *putP* → | proline:sodium symporter |
| 1,134,456 | IS*150* (–) +3 bp | 100% |  |  |  |  |  |  |  |  |  |  |  | coding (179‑181/567 nt) | *yceJ* ← | predicted cytochrome b561 |
| 1,137,052 | IS*150* (+) +3 bp |  |  | 100% |  |  |  |  |  |  |  |  |  | coding (27‑29/246 nt) | *dinI* ← | DNA damage‑inducible protein I |
| 1,154,915 | IS*150* (+) +3 bp | 100% |  |  |  |  |  | 100% |  |  |  |  |  | coding (948‑950/1644 nt) | *flgK* → | flagellar hook‑associated protein K |
| 1,181,558 | C→T |  |  | 100% |  |  |  |  |  | 100% | 100% |  |  | intergenic (+283/‑125) | *ycfP* → / → *ndh* | hypothetical protein/respiratory NADH dehydrogenase 2/cupric reductase |
| 1,205,342 | T→G |  |  |  |  |  | 100% |  |  |  |  |  |  | T276P (ACC→CCC) | *phoQ* ← | sensory histidine kinase in two‑compoent regulatory system with PhoP |
| 1,205,348 | C→A |  |  |  |  |  |  |  | 100% |  |  |  |  | D274Y (GAC→TAC) | *phoQ* ← | sensory histidine kinase in two‑compoent regulatory system with PhoP |
| 1,216,074 | (T)7→8 |  |  |  |  |  |  |  |  |  |  | 100% |  | coding (584/1212 nt) | *ycgF* ← | predicted FAD‑binding phosphodiesterase |
| 1,236,016 | IS*150* (–) +3 bp |  |  |  |  |  |  | 100% |  |  |  |  |  | intergenic (‑128/‑91) | *nhaB* ← / → *fadR* | sodium/proton antiporter/fatty acid metabolism regulator |
| 1,236,016 | IS*150* (+) +3 bp |  |  |  | 100% |  |  |  |  |  |  |  |  | intergenic (‑128/‑91) | *nhaB* ← / → *fadR* | sodium/proton antiporter/fatty acid metabolism regulator |
| position | mutation | ZDBp871\_minus\_CZB151 | ZDBp875\_minus\_CZB151 | ZDBp877\_minus\_CZB152 | ZDBp880\_minus\_CZB152 | ZDBp883\_minus\_CZB154 | ZDBp886\_minus\_CZB154 | ZDBp889\_minus\_ZDB67 | ZDBp892\_minus\_ZDB67 | ZDBp895\_minus\_ZDB68 | ZDBp898\_minus\_ZDB68 | ZDBp901\_minus\_ZDB69 | ZDBp904\_minus\_ZDB69 | annotation | gene | description |
| 1,236,018 | IS*150* (–) +2 bp :: +TC |  | 100% |  |  |  |  |  |  |  |  |  |  | intergenic (‑130/‑90) | *nhaB* ← / → *fadR* | sodium/proton antiporter/fatty acid metabolism regulator |
| 1,257,394 | A→G |  |  |  |  |  |  |  |  |  | 100% |  |  | intergenic (‑271/+498) | *ycgV* ← / ← *ychF* | predicted adhesin/translation‑associated GTPase |
| 1,271,135 | Δ15 bp |  |  |  |  |  |  |  | 100% |  |  |  |  | IS*150*‑mediated | *ldrB* ← / ← *insK‑2* | toxic polypeptide, small/IS150 putative transposase |
| 1,294,964 | IS*3* (–) +4 bp :: +TC |  | 100% |  |  |  |  |  |  |  |  |  |  | intergenic (‑315/‑286) | *hns* ← / → *tdk* | global DNA‑binding transcriptional dual regulator H‑NS/thymidine kinase |
| 1,306,724 | Δ1 bp |  |  |  |  |  |  | 100% |  |  |  |  |  | coding (1449/1461 nt) | *cls* ← | cardiolipin synthetase |
| 1,306,894 | IS*150* (–) +3 bp |  |  |  |  |  |  |  |  |  | 100% |  |  | coding (1277‑1279/1461 nt) | *cls* ← | cardiolipin synthetase |
| 1,307,420 | IS*150* (–) +3 bp |  |  | 100% |  |  |  |  |  |  |  |  |  | coding (751‑753/1461 nt) | *cls* ← | cardiolipin synthetase |
| 1,334,535 | A→C |  | 100% |  |  |  |  |  |  |  |  |  |  | M126L (ATG→CTG) | *cysB* → | DNA‑binding transcriptional dual regulator, O‑acetyl‑L‑serine‑binding |
| 1,362,312 | Δ1 bp | 100% |  |  |  |  |  |  |  |  |  |  |  | coding (101/558 nt) | *ycjC* → | DNA‑binding transcriptional repressor |
| 1,423,903 | Δ23,962 bp |  |  |  |  |  |  |  |  |  |  |  | 100% | between IS*3* | *ECB\_01341*–*[ydbC]* | **23 genes***ECB\_01341*, *ECB\_01342*, *ECB\_01343*, *ECB\_01344*, *pinR*, *ynaE*, *ynaF*, *ompN*, *insB‑10*, *insA‑10*, *ydbK*, *ydbJ*, *hslJ*, *ldhA*, *ydbH*, *ynbE*, *ydbL*, *feaR*, *feaB*, *tynA*, *insE‑2*, *insF‑2*, *[ydbC]* *ECB\_01341*, *ECB\_01342*, *ECB\_01343*, *ECB\_01344*, *pinR*, *ynaE*, *ynaF*, *ompN*, *insB‑10*, *insA‑10*, *ydbK*, *ydbJ*, *hslJ*, *ldhA*, *ydbH*, *ynbE*, *ydbL*, *feaR*, *feaB*, *tynA*, *insE‑2*, *insF‑2*, *[ydbC]* |
| position | mutation | ZDBp871\_minus\_CZB151 | ZDBp875\_minus\_CZB151 | ZDBp877\_minus\_CZB152 | ZDBp880\_minus\_CZB152 | ZDBp883\_minus\_CZB154 | ZDBp886\_minus\_CZB154 | ZDBp889\_minus\_ZDB67 | ZDBp892\_minus\_ZDB67 | ZDBp895\_minus\_ZDB68 | ZDBp898\_minus\_ZDB68 | ZDBp901\_minus\_ZDB69 | ZDBp904\_minus\_ZDB69 | annotation | gene | description |
| 1,457,389 | Δ11,725 bp | 100% | Δ | 100% |  | 100% |  |  | 100% | 100% | Δ | 100% | 100% | between IS*150* | *hrpA*–*insJ‑2* | *hrpA*, *ydcF*, *aldA*, *gapC*, *insA‑12*, *insB‑12*, *cybB*, *ydcA*, *hokB*, *mokB*, *insK‑2*, *insJ‑2* |
| 1,457,389 | Δ13,493 bp |  | Δ |  |  |  |  |  |  |  | 100% |  |  | IS*150*‑mediated | *hrpA*–*[trg]* | *hrpA*, *ydcF*, *aldA*, *gapC*, *insA‑12*, *insB‑12*, *cybB*, *ydcA*, *hokB*, *mokB*, *insK‑2*, *insJ‑2*, *[trg]* |
| 1,457,389 | Δ14,145 bp |  | 100% |  |  |  |  |  |  |  |  |  |  | IS*150*‑mediated | *hrpA*–*[ydcI]* | *hrpA*, *ydcF*, *aldA*, *gapC*, *insA‑12*, *insB‑12*, *cybB*, *ydcA*, *hokB*, *mokB*, *insK‑2*, *insJ‑2*, *trg*, *[ydcI]* |
| 1,466,345 | Δ7,319 bp |  |  |  |  |  |  | 100% |  |  |  |  |  | IS*1*‑mediated | *cybB*–*ydcJ* | *cybB*, *ydcA*, *hokB*, *mokB*, *insK‑2*, *insJ‑2*, *trg*, *ydcI*, *ydcJ* |
| 1,528,089 | IS*150* (+) +4 bp |  |  |  |  |  |  |  |  | 100% |  |  |  | intergenic (‑33/‑253) | *yddG* ← / → *fdnG* | predicted methyl viologen efflux pump/formate dehydrogenase‑N, alpha subunit, nitrate‑inducible |
| 1,536,594 | +GA :: IS*3* (+) +4 bp |  |  |  | 100% |  |  |  |  |  |  |  |  | coding (99‑102/138 nt) | *rpsV* ← | 30S ribosomal subunit protein S22 |
| 1,536,758 | +TGA :: IS*3* (+) +3 bp |  |  |  |  |  |  |  |  | 100% |  |  |  | intergenic (‑63/+37) | *rpsV* ← / ← *bdm* | 30S ribosomal subunit protein S22/biofilm‑dependent modulation protein |
| 1,567,292 | IS*1* (+) +9 bp |  |  |  |  | 100% |  |  |  |  |  |  |  | intergenic (‑61/+265) | *ydeP* ← / ← *ydeQ* | predicted oxidoreductase/predicted fimbrial‑like adhesin protein |
| 1,595,625 | IS*150* (+) +3 bp |  |  |  |  |  |  |  |  | 100% |  |  |  | coding (71‑73/900 nt) | *eamA* ← | cysteine and O‑acetyl‑L‑serine efflux system |
| 1,619,073 | IS*150* (–) +3 bp |  |  |  |  |  | 100% | 100% |  |  |  |  |  | intergenic (‑71/+139) | *hokD* ← / ← *ECB\_01533* | small toxic polypeptide/conserved hypothetical protein |
| position | mutation | ZDBp871\_minus\_CZB151 | ZDBp875\_minus\_CZB151 | ZDBp877\_minus\_CZB152 | ZDBp880\_minus\_CZB152 | ZDBp883\_minus\_CZB154 | ZDBp886\_minus\_CZB154 | ZDBp889\_minus\_ZDB67 | ZDBp892\_minus\_ZDB67 | ZDBp895\_minus\_ZDB68 | ZDBp898\_minus\_ZDB68 | ZDBp901\_minus\_ZDB69 | ZDBp904\_minus\_ZDB69 | annotation | gene | description |
| 1,640,803 | IS*150* (–) +3 bp |  | 100% |  |  |  |  |  |  |  |  |  |  | coding (761‑763/855 nt) | *ynfH* → | oxidoreductase, membrane subunit |
| 1,651,190 | IS*150* (–) +3 bp | 100% |  |  |  |  |  |  |  |  |  |  |  | coding (844‑846/1035 nt) | *ydgG* → | predicted inner membrane protein |
| 1,651,192 | IS*150* (–) +3 bp :: +TACA |  |  |  |  |  | 100% |  |  |  |  |  |  | coding (846‑848/1035 nt) | *ydgG* → | predicted inner membrane protein |
| 1,651,206 | IS*150* (+) +3 bp |  |  |  |  |  |  |  |  | 100% |  |  |  | coding (860‑862/1035 nt) | *ydgG* → | predicted inner membrane protein |
| 1,729,737 | Δ2,150 bp |  |  |  |  |  |  |  |  |  | 100% |  |  | IS*150*‑mediated | *ydhZ*–*[pykF]* | *ydhZ*, *[pykF]* |
| 1,729,741 | Δ1 bp |  |  |  |  |  |  | 100% |  |  | Δ |  |  | intergenic (‑52/+698) | *insJ‑2* ← / ← *ydhZ* | IS150 hypothetical protein/hypothetical protein |
| 1,776,123 | +T | 100% |  |  |  |  |  |  |  |  |  |  |  | intergenic (‑50/+57) | *insJ‑2* ← / ← *pheM* | IS150 hypothetical protein/phenylalanyl‑tRNA synthetase operon leader peptide |
| 1,789,698 | (T)6→5 |  |  |  |  |  |  |  |  |  | 100% |  |  | coding (399/795 nt) | *ydjO* ← | hypothetical protein |
| 1,802,927 | IS*1* (+) +9 bp |  |  |  |  |  |  |  |  | 100% |  |  |  | coding (941‑949/969 nt) | *astE* ← | succinylglutamate desuccinylase |
| 1,821,169 | IS*150* (+) +3 bp |  |  |  |  |  |  |  |  |  |  |  | 100% | coding (616‑618/1041 nt) | *ynjI* ← | predicted inner membrane protein |
| position | mutation | ZDBp871\_minus\_CZB151 | ZDBp875\_minus\_CZB151 | ZDBp877\_minus\_CZB152 | ZDBp880\_minus\_CZB152 | ZDBp883\_minus\_CZB154 | ZDBp886\_minus\_CZB154 | ZDBp889\_minus\_ZDB67 | ZDBp892\_minus\_ZDB67 | ZDBp895\_minus\_ZDB68 | ZDBp898\_minus\_ZDB68 | ZDBp901\_minus\_ZDB69 | ZDBp904\_minus\_ZDB69 | annotation | gene | description |
| 1,867,822 | IS*150* (–) +3 bp |  |  |  |  |  |  |  |  | 100% |  |  |  | coding (188‑190/1686 nt) | *fadD* ← | acyl‑CoA synthase |
| 1,867,855 | +CTT |  |  |  |  |  |  |  |  |  | 100% |  |  | coding (157/1686 nt) | *fadD* ← | acyl‑CoA synthase |
| 1,887,034 | IS*150* (+) +3 bp |  | 100% |  |  |  |  |  |  |  |  |  |  | intergenic (‑3/‑154) | *yobG* ← / → *ECB\_01797* | hypothetical protein/hypothetical protein |
| 1,887,041 | IS*1* (–) +9 bp |  |  |  |  |  |  | 100% |  |  |  |  |  | intergenic (‑10/‑141) | *yobG* ← / → *ECB\_01797* | hypothetical protein/hypothetical protein |
| 1,887,041 | IS*1* (+) +9 bp |  |  | 100% |  |  |  |  |  |  |  |  |  | intergenic (‑10/‑141) | *yobG* ← / → *ECB\_01797* | hypothetical protein/hypothetical protein |
| 1,887,084 | IS*1* (+) +9 bp |  |  |  |  |  |  |  |  |  | 100% |  |  | intergenic (‑53/‑98) | *yobG* ← / → *ECB\_01797* | hypothetical protein/hypothetical protein |
| 1,896,278 | IS*150* (–) +4 bp |  |  |  |  |  |  |  |  |  |  |  | 100% | coding (505‑508/2634 nt) | *yebT* → | hypothetical protein |
| 1,897,614 | IS*150* (–) +3 bp | 100% |  |  |  |  |  |  |  |  |  |  |  | coding (1841‑1843/2634 nt) | *yebT* → | hypothetical protein |
| 1,902,189 | IS*150* (–) +3 bp |  | 100% |  |  |  |  |  |  |  |  |  |  | coding (664‑666/873 nt) | *yebZ* ← | predicted inner membrane protein |
| 1,913,929 | IS*150* (–) +3 bp |  |  |  |  |  |  |  |  |  | 100% |  |  | coding (647‑649/1476 nt) | *zwf* ← | glucose‑6‑phosphate 1‑dehydrogenase |
| position | mutation | ZDBp871\_minus\_CZB151 | ZDBp875\_minus\_CZB151 | ZDBp877\_minus\_CZB152 | ZDBp880\_minus\_CZB152 | ZDBp883\_minus\_CZB154 | ZDBp886\_minus\_CZB154 | ZDBp889\_minus\_ZDB67 | ZDBp892\_minus\_ZDB67 | ZDBp895\_minus\_ZDB68 | ZDBp898\_minus\_ZDB68 | ZDBp901\_minus\_ZDB69 | ZDBp904\_minus\_ZDB69 | annotation | gene | description |
| 1,988,179 | IS*150* (+) +3 bp |  |  | 100% |  |  |  |  |  |  |  |  |  | coding (5631‑5633/7152 nt) | *yeeJ* → | adhesin |
| 2,079,174 | IS*150* (+) +3 bp |  |  |  |  |  | 100% |  |  |  |  |  |  | coding (613‑615/819 nt) | *yegX* ← | predicted hydrolase |
| 2,099,889 | IS*150* (–) +3 bp |  |  |  |  | 100% |  |  |  |  |  |  |  | coding (991‑993/2280 nt) | *yehM* → | hypothetical protein |
| 2,133,554 | IS*150* (+) +3 bp |  |  |  |  |  |  |  |  | 100% | ? |  |  | coding (39‑41/999 nt) | *mglB* ← | methyl‑galactoside transporter subunit |
| 2,133,567 | IS*150* (+) +4 bp |  |  |  | 100% |  |  |  |  |  | ? |  |  | coding (25‑28/999 nt) | *mglB* ← | methyl‑galactoside transporter subunit |
| 2,133,582 | IS*150* (+) +3 bp |  |  |  |  |  |  |  |  |  | ? |  | 100% | coding (11‑13/999 nt) | *mglB* ← | methyl‑galactoside transporter subunit |
| 2,209,801 | A→T | 100% |  |  |  |  |  |  |  |  |  |  |  | S351C (AGT→TGT) | *atoS* → | sensory histidine kinase in two‑component regulatory system with AtoC |
| 2,209,853 | C→A |  |  |  |  | 100% |  |  |  |  |  |  |  | S368Y (TCT→TAT) | *atoS* → | sensory histidine kinase in two‑component regulatory system with AtoC |
| 2,230,905 | IS*150* (+) +3 bp |  |  |  |  |  |  |  |  |  |  |  | 100% | coding (1971‑1973/3753 nt) | *yfaL* ← | adhesin |
| 2,302,013 | G→A |  | 100% |  |  |  |  |  |  |  |  |  |  | A556T (GCG→ACG) | *pta* → | phosphate acetyltransferase |
| position | mutation | ZDBp871\_minus\_CZB151 | ZDBp875\_minus\_CZB151 | ZDBp877\_minus\_CZB152 | ZDBp880\_minus\_CZB152 | ZDBp883\_minus\_CZB154 | ZDBp886\_minus\_CZB154 | ZDBp889\_minus\_ZDB67 | ZDBp892\_minus\_ZDB67 | ZDBp895\_minus\_ZDB68 | ZDBp898\_minus\_ZDB68 | ZDBp901\_minus\_ZDB69 | ZDBp904\_minus\_ZDB69 | annotation | gene | description |
| 2,325,035 | IS*150* (+) +3 bp |  |  |  | 100% |  |  |  |  |  |  |  |  | coding (685‑687/1179 nt) | *yfcJ* ← | predicted transporter |
| 2,331,208 | IS*RSO11* (+) +4 bp |  |  |  |  |  |  |  | 100% |  |  |  |  | coding (775‑778/825 nt) | *mepA* ← | penicillin‑insensitive murein endopeptidase |
| 2,346,173 | G→A |  |  |  |  |  | 100% |  |  |  |  |  |  | intergenic (‑31/+150) | *yfcY* ← / ← *yfcZ* | acetyl‑CoA acetyltransferase/hypothetical protein |
| 2,346,183 | G→A |  |  |  |  |  |  |  |  |  |  |  | 100% | intergenic (‑41/+140) | *yfcY* ← / ← *yfcZ* | acetyl‑CoA acetyltransferase/hypothetical protein |
| 2,347,259 | IS*150* (–) +3 bp |  | 100% |  |  |  |  |  |  |  |  |  |  | coding (287‑289/1347 nt) | *fadL* → | long‑chain fatty acid outer membrane transporter |
| 2,348,048 | IS*150* (–) +3 bp |  |  | 100% |  |  |  |  |  |  |  |  |  | coding (1076‑1078/1347 nt) | *fadL* → | long‑chain fatty acid outer membrane transporter |
| 2,348,062 | IS*3* (–) +3 bp :: +TCA | 100% |  |  |  |  |  |  |  |  |  |  |  | coding (1090‑1092/1347 nt) | *fadL* → | long‑chain fatty acid outer membrane transporter |
| 2,348,064 | IS*150* (+) +3 bp |  |  |  |  | 100% |  |  |  |  |  |  |  | coding (1092‑1094/1347 nt) | *fadL* → | long‑chain fatty acid outer membrane transporter |
| 2,348,272 | IS*150* (–) +3 bp |  |  |  |  |  | 100% | 100% |  |  |  |  |  | coding (1300‑1302/1347 nt) | *fadL* → | long‑chain fatty acid outer membrane transporter |
| 2,434,162 | G→T |  |  |  |  |  |  | 100% |  |  |  |  |  | S121R (AGC→AGA) | *eutJ* ← | predicted chaperonin, ethanolamine utilization protein |
| position | mutation | ZDBp871\_minus\_CZB151 | ZDBp875\_minus\_CZB151 | ZDBp877\_minus\_CZB152 | ZDBp880\_minus\_CZB152 | ZDBp883\_minus\_CZB154 | ZDBp886\_minus\_CZB154 | ZDBp889\_minus\_ZDB67 | ZDBp892\_minus\_ZDB67 | ZDBp895\_minus\_ZDB68 | ZDBp898\_minus\_ZDB68 | ZDBp901\_minus\_ZDB69 | ZDBp904\_minus\_ZDB69 | annotation | gene | description |
| 2,455,036 | A→G |  |  |  |  |  | 100% |  |  |  |  |  |  | intergenic (+142/‑397) | *acrD* → / → *yffB* | aminoglycoside/multidrug efflux system/hypothetical protein |
| 2,465,970 | IS*186* (–) +8 bp |  |  | 100% |  |  |  |  |  |  |  |  |  | coding (584‑591/618 nt) | *hyfA* → | hydrogenase 4, 4Fe‑4S subunit |
| 2,468,533 | A→C |  |  |  |  |  |  |  |  |  | 100% |  |  | intergenic (+511/‑549) | *hyfB* → / → *hyfD* | NADH dehydrogenase subunit N/hydrogenase 4 membrane subunit |
| 2,525,967 | (CTAATTACTTCGCCAACGGCG)1→2 |  |  |  |  |  |  |  |  |  |  |  | 100% | coding (222/489 nt) | *iscR* ← | DNA‑binding transcriptional repressor |
| 2,526,007 | G→A |  |  |  |  | 100% |  |  |  |  |  |  |  | P61L (CCA→CTA) | *iscR* ← | DNA‑binding transcriptional repressor |
| 2,527,313 | C→A |  |  | 100% |  |  |  |  |  |  |  |  |  | R23L (CGT→CTT) | *yfhQ* ← | predicted methyltransferase |
| 2,529,490 | +ACA |  |  | 100% |  |  |  |  |  |  |  |  |  | intergenic (+189/‑2) | *yfhR* → / → *csiE* | predicted peptidase/stationary phase inducible protein |
| 2,535,008 | IS*150* (+) +1 bp :: +AA | 100% |  |  |  |  |  |  |  |  |  |  |  | coding (44/321 nt) | *hcaC* → | 3‑phenylpropionate dioxygenase, predicted ferredoxin subunit |
| 2,549,863 | C→T |  |  |  |  |  |  |  |  | 100% |  |  |  | intergenic (‑299/‑29) | *glyA* ← / → *hmpA* | serine hydroxymethyltransferase/fused nitric oxide dioxygenase/dihydropteridine reductase 2 |
| 2,600,587 | +T | 100% |  |  |  |  |  |  |  |  |  |  |  | intergenic (‑51/+655) | *insJ‑2* ← / ← *rluD* | IS150 hypothetical protein/23S rRNA pseudouridine synthase |
| position | mutation | ZDBp871\_minus\_CZB151 | ZDBp875\_minus\_CZB151 | ZDBp877\_minus\_CZB152 | ZDBp880\_minus\_CZB152 | ZDBp883\_minus\_CZB154 | ZDBp886\_minus\_CZB154 | ZDBp889\_minus\_ZDB67 | ZDBp892\_minus\_ZDB67 | ZDBp895\_minus\_ZDB68 | ZDBp898\_minus\_ZDB68 | ZDBp901\_minus\_ZDB69 | ZDBp904\_minus\_ZDB69 | annotation | gene | description |
| 2,623,300 | IS*3* (–) +3 bp :: +TCA |  |  |  |  |  |  | 100% |  |  |  |  |  | coding (1081‑1083/1230 nt) | *ECB\_02509* → | Fels‑2 prophage protein |
| 2,630,053 | A→C |  |  |  |  |  |  | 100% | 100% |  |  |  |  | I197L (ATT→CTT) | *ygaF* → | predicted enzyme |
| 2,650,572 | G→T | 100% |  |  |  |  |  |  |  |  |  |  |  | M47I (ATG→ATT) | *emrR* → | DNA‑binding transcriptional repressor of microcin B17 synthesis and multidrug efflux |
| 2,663,500 | C→T |  |  |  |  |  |  |  | 100% |  |  |  |  | intergenic (‑69/+11) | *recA* ← / ← *ygaD* | recombinase A/competence damage‑inducible protein A |
| 2,720,682 | Δ1 bp |  |  |  |  | 100% |  |  | 100% |  |  |  |  | intergenic (‑49/+200) | *insJ‑3* ← / ← *cysH* | IS150 hypothetical protein/phosphoadenosine phosphosulfate reductase |
| 2,769,779 | G→A |  |  |  |  |  |  |  |  |  | 100% |  |  | R415\* (CGA→TGA) | *ECB\_02649* ← | protein similar to L‑ribulokinase AraB |
| 2,845,864 | Δ2,335 bp |  |  |  |  |  |  |  |  |  | 100% |  |  | IS*150*‑mediated | *yqeC*–*[ygfK]* | *yqeC*, *ygfJ*, *[ygfK]* |
| 2,897,655 | IS*1* (+) +8 bp | 100% |  |  |  |  |  |  |  |  |  |  |  | coding (854‑861/1479 nt) | *ygfH* → | propionyl‑CoA:succinate‑CoA transferase |
| 2,914,014 | IS*150* (+) +3 bp |  |  | 100% |  |  |  |  |  |  |  |  |  | coding (102‑104/759 nt) | *yggG* → | predicted peptidase |
| 2,938,642 | IS*150* (–) +3 bp |  |  |  |  |  |  | 100% |  |  |  |  |  | coding (1019‑1021/1257 nt) | *nupG* → | nucleoside transporter |
| position | mutation | ZDBp871\_minus\_CZB151 | ZDBp875\_minus\_CZB151 | ZDBp877\_minus\_CZB152 | ZDBp880\_minus\_CZB152 | ZDBp883\_minus\_CZB154 | ZDBp886\_minus\_CZB154 | ZDBp889\_minus\_ZDB67 | ZDBp892\_minus\_ZDB67 | ZDBp895\_minus\_ZDB68 | ZDBp898\_minus\_ZDB68 | ZDBp901\_minus\_ZDB69 | ZDBp904\_minus\_ZDB69 | annotation | gene | description |
| 2,957,887 | IS*150* (+) +3 bp |  |  |  |  |  | 100% |  |  |  |  |  |  | intergenic (‑78/‑494) | *ECB\_02812* ← / → *ECB\_02813* | conserved hypothetical protein/hypothetical protein |
| 2,963,466 | Δ13,009 bp |  | 100% |  |  |  |  |  |  |  |  |  |  | IS*150*‑mediated | *ECB\_02837*–*[yghK]* | *ECB\_02837*, *ECB\_02838*, *yghF*, *yghG*, *pppA*, *yghJ*, *[yghK]* |
| 3,109,394 | IS*150* (–) +3 bp |  |  |  |  |  | 100% |  | 100% |  |  |  |  | coding (245‑247/663 nt) | *yqjA* → | conserved inner membrane protein |
| 3,109,394 | IS*150* (–) +4 bp |  |  |  |  | 100% |  |  |  |  |  | 100% |  | coding (245‑248/663 nt) | *yqjA* → | conserved inner membrane protein |
| 3,110,349 | IS*3* (–) +3 bp :: +TCA |  |  |  |  |  |  |  |  | 100% |  |  |  | coding (4‑6/369 nt) | *yqjC* → | hypothetical protein |
| 3,172,495 | Δ1 bp |  |  |  |  | 100% |  |  |  |  |  |  |  | coding (7/885 nt) | *nlpI* ← | hypothetical protein |
| 3,172,540 | IS*150* (–) +3 bp |  |  | 100% |  |  |  |  | 100% |  |  |  |  | intergenic (‑39/+68) | *nlpI* ← / ← *pnp* | hypothetical protein/polynucleotide phosphorylase/polyadenylase |
| 3,196,333 | Δ59 bp |  |  |  | 100% |  |  |  |  | ? |  |  |  | IS*150*‑mediated | *yhbE* ← / → *insJ‑2* | conserved inner membrane protein/IS150 hypothetical protein |
| 3,242,824 | A→C | 100% |  |  |  |  |  |  |  |  |  |  |  | I148L (ATT→CTT) | *argR* → | arginine repressor |
| 3,290,790 | Δ1 bp |  |  |  |  | 100% |  |  |  |  |  |  |  | coding (459/1125 nt) | *smf* ← | hypothetical protein |
| position | mutation | ZDBp871\_minus\_CZB151 | ZDBp875\_minus\_CZB151 | ZDBp877\_minus\_CZB152 | ZDBp880\_minus\_CZB152 | ZDBp883\_minus\_CZB154 | ZDBp886\_minus\_CZB154 | ZDBp889\_minus\_ZDB67 | ZDBp892\_minus\_ZDB67 | ZDBp895\_minus\_ZDB68 | ZDBp898\_minus\_ZDB68 | ZDBp901\_minus\_ZDB69 | ZDBp904\_minus\_ZDB69 | annotation | gene | description |
| 3,393,071 | IS*150* (–) +3 bp |  |  |  |  |  |  |  |  |  | 100% |  |  | coding (1263‑1265/1353 nt) | *envZ* ← | osmolarity sensor protein |
| 3,402,005 | IS*150* (–) +1 bp :: +ATC | 100% |  |  |  |  |  |  |  |  |  |  |  | coding (391/879 nt) | *yhgA* → | predicted transposase |
| 3,427,815 | T→C |  |  |  |  |  |  |  | 100% |  |  |  |  | N2D (AAT→GAT) | *glgP* ← | glycogen phosphorylase |
| 3,429,542 | C→A |  |  |  |  |  |  |  |  |  | 100% |  |  | G349C (GGT→TGT) | *glgC* ← | glucose‑1‑phosphate adenylyltransferase |
| 3,501,352 | +A :: IS*150* (+) +3 bp |  |  |  |  |  |  |  |  |  |  | 100% |  | coding (188‑190/336 nt) | *yhiO* ← | universal stress protein UspB |
| 3,501,576 | IS*150* (–) +3 bp | 100% |  |  |  |  |  |  |  |  |  |  |  | intergenic (‑35/‑354) | *yhiO* ← / → *uspA* | universal stress protein UspB/universal stress global response regulator |
| 3,501,576 | IS*150* (+) +3 bp |  | 100% | 100% | 100% |  | 100% |  | 100% | 100% |  |  | 100% | intergenic (‑35/‑354) | *yhiO* ← / → *uspA* | universal stress protein UspB/universal stress global response regulator |
| 3,501,576 | IS*150* (+) +3 bp :: +C |  |  |  |  | 100% |  |  |  |  |  |  |  | intergenic (‑35/‑354) | *yhiO* ← / → *uspA* | universal stress protein UspB/universal stress global response regulator |
| 3,501,577 | IS*150* (–) +2 bp |  |  |  |  |  |  | 100% |  |  |  |  |  | intergenic (‑36/‑354) | *yhiO* ← / → *uspA* | universal stress protein UspB/universal stress global response regulator |
| 3,536,960 | IS*150* (–) +3 bp |  |  |  |  |  |  |  |  | 100% |  |  |  | coding (2028‑2030/2076 nt) | *yhjG* ← | predicted outer membrane biogenesis protein |
| position | mutation | ZDBp871\_minus\_CZB151 | ZDBp875\_minus\_CZB151 | ZDBp877\_minus\_CZB152 | ZDBp880\_minus\_CZB152 | ZDBp883\_minus\_CZB154 | ZDBp886\_minus\_CZB154 | ZDBp889\_minus\_ZDB67 | ZDBp892\_minus\_ZDB67 | ZDBp895\_minus\_ZDB68 | ZDBp898\_minus\_ZDB68 | ZDBp901\_minus\_ZDB69 | ZDBp904\_minus\_ZDB69 | annotation | gene | description |
| 3,538,891 | IS*150* (+) +3 bp |  |  |  |  |  |  | 100% |  |  |  |  |  | coding (97‑99/2076 nt) | *yhjG* ← | predicted outer membrane biogenesis protein |
| 3,544,110 | A→G |  |  | 100% |  |  |  |  |  |  |  |  |  | intergenic (‑39/+144) | *dctA* ← / ← *yhjK* | C4‑dicarboxylate transport protein/predicted diguanylate cyclase |
| 3,561,766 | IS*150* (–) +3 bp | 100% |  |  |  |  |  |  |  |  |  |  |  | intergenic (‑89/‑385) | *ldrD* ← / → *yhjV* | toxic polypeptide, small/predicted transporter |
| 3,577,388 | Δ4,835 bp |  |  |  |  |  |  |  | 100% |  |  |  |  | IS*150*‑mediated | *[bisC]*–*hokA* | *[bisC]*, *yiaD*, *tkrA*, *yiaF*, *yiaG*, *cspA*, *hokA* |
| 3,583,666 | Δ100 bp |  |  |  |  |  |  | 100% |  |  |  |  |  | IS*150*‑mediated | *insK‑4* → / ← *glyS* | IS150 putative transposase/glycyl‑tRNA synthetase subunit beta |
| 3,583,667 | Δ1 bp |  |  |  |  |  |  | Δ |  |  | 100% |  |  | intergenic (+28/+251) | *insK‑4* → / ← *glyS* | IS150 putative transposase/glycyl‑tRNA synthetase subunit beta |
| 3,687,738 | Δ1 bp :: IS*186* (+) +9 bp |  |  |  |  | 100% |  |  |  |  |  |  |  | coding (306‑314/864 nt) | *yicC* → | hypothetical protein |
| 3,700,202 | IS*1* (–) +8 bp |  |  | 100% |  |  |  |  |  |  |  |  |  | intergenic (‑94/‑179) | *gltS* ← / → *yicE* | glutamate transporter/predicted transporter |
| 3,748,889 | IS*150* (–) +3 bp |  |  | 100% |  |  |  |  |  |  |  |  |  | coding (142‑144/450 nt) | *yidI* → | predicted inner membrane protein |
| 3,756,769 | IS*1* (–) +8 bp | 100% |  |  |  |  |  |  |  |  |  |  |  | intergenic (‑325/+598) | *glvBC* ← / ← *yidE* | arbutin specific enzyme IIBC component of PTS/hypothetical protein |
| position | mutation | ZDBp871\_minus\_CZB151 | ZDBp875\_minus\_CZB151 | ZDBp877\_minus\_CZB152 | ZDBp880\_minus\_CZB152 | ZDBp883\_minus\_CZB154 | ZDBp886\_minus\_CZB154 | ZDBp889\_minus\_ZDB67 | ZDBp892\_minus\_ZDB67 | ZDBp895\_minus\_ZDB68 | ZDBp898\_minus\_ZDB68 | ZDBp901\_minus\_ZDB69 | ZDBp904\_minus\_ZDB69 | annotation | gene | description |
| 3,764,589 | IS*150* (+) +3 bp |  |  |  |  |  | 100% |  |  |  |  |  |  | intergenic (‑97/+21) | *dgoT* ← / ← *dgoD* | D‑galactonate transporter/galactonate dehydratase |
| 3,825,949 | Δ1 bp |  |  |  |  | 100% |  |  |  |  |  |  |  | intergenic (+3/‑51) | *kup* → / → *insJ‑5* | potassium transporter/IS150 hypothetical protein |
| 3,888,290 | IS*150* (–) +3 bp |  |  |  |  |  |  |  |  | 100% |  |  |  | coding (586‑588/903 nt) | *ECB\_03690* ← | conserved hypothetical protein |
| 3,932,217 | IS*150* (+) +3 bp | 100% |  |  |  |  |  |  |  |  |  |  |  | coding (865‑867/1164 nt) | *fadA* ← | acetyl‑CoA acetyltransferase |
| 3,975,518 | IS*150* (–) +3 bp |  | 100% |  | 100% |  |  |  |  |  |  |  |  | coding (322‑324/1242 nt) | *yihS* ← | predicted glucosamine isomerase |
| 3,975,518 | IS*150* (+) +3 bp | 100% |  |  |  |  |  |  |  |  |  |  |  | coding (322‑324/1242 nt) | *yihS* ← | predicted glucosamine isomerase |
| 4,035,958 | Δ220 bp | 100% |  |  |  |  |  |  |  |  |  |  |  | IS*150*‑mediated | *metB* → / ← *insK‑2* | cystathionine gamma‑synthase/IS150 putative transposase |
| 4,090,512 | G→A |  |  |  |  |  |  |  |  |  |  | 100% |  | R758H (CGT→CAT) | *rpoB* → | DNA‑directed RNA polymerase subunit beta |
| 4,091,274 | A→C | 100% |  |  |  |  |  |  |  |  |  |  |  | E1012A (GAA→GCA) | *rpoB* → | DNA‑directed RNA polymerase subunit beta |
| 4,122,754 | IS*150* (–) +3 bp |  |  |  |  |  |  |  |  |  |  | 100% |  | coding (448‑450/1602 nt) | *aceB* → | malate synthase |
| position | mutation | ZDBp871\_minus\_CZB151 | ZDBp875\_minus\_CZB151 | ZDBp877\_minus\_CZB152 | ZDBp880\_minus\_CZB152 | ZDBp883\_minus\_CZB154 | ZDBp886\_minus\_CZB154 | ZDBp889\_minus\_ZDB67 | ZDBp892\_minus\_ZDB67 | ZDBp895\_minus\_ZDB68 | ZDBp898\_minus\_ZDB68 | ZDBp901\_minus\_ZDB69 | ZDBp904\_minus\_ZDB69 | annotation | gene | description |
| 4,134,638 | T→C |  |  |  |  |  |  |  |  |  |  | 100% |  | L198P (CTG→CCG) | *yjbB* → | predicted transporter |
| 4,135,292 | A→C |  |  |  |  |  | 100% |  |  |  |  |  |  | D416A (GAT→GCT) | *yjbB* → | predicted transporter |
| 4,188,718 | C→T |  | 100% |  |  |  |  |  |  |  |  |  |  | M225I (ATG→ATA) | *yjcF* ← | hypothetical protein |
| 4,191,720 | IS*150* (+) +4 bp |  |  |  |  |  |  |  |  |  | 100% |  |  | intergenic (‑190/+7) | *yjcH* ← / ← *acs* | conserved inner membrane protein involved in acetate transport/acetyl‑coenzyme A synthetase |
| 4,203,100 | IS*150* (–) +3 bp |  |  |  |  |  |  |  |  |  | 100% |  |  | coding (230‑232/690 nt) | *yjcO* ← | hypothetical protein |
| 4,234,238 | IS*150* (+) +3 bp |  |  |  |  |  | 100% |  |  |  |  |  |  | coding (1006‑1008/2229 nt) | *yjdA* → | conserved protein with nucleoside triphosphate hydrolase domain |
| 4,256,901 | (CGCGG)3→2 |  |  |  |  |  |  |  |  |  |  | 100% |  | intergenic (‑768/‑1042) | *dcuR* ← / → *yjdI* | DNA‑binding response regulator in two‑component regulatory system with DcuS/hypothetical protein |
| 4,260,611 | IS*150* (–) +4 bp | 100% |  |  |  |  |  |  |  |  |  |  |  | coding (208‑211/1518 nt) | *lysU* ← | lysine tRNA synthetase, inducible |
| 4,260,612 | IS*150* (–) +3 bp |  | 100% |  |  |  |  |  |  |  |  |  |  | coding (208‑210/1518 nt) | *lysU* ← | lysine tRNA synthetase, inducible |
| 4,307,583 | Δ61 bp |  |  |  |  |  |  |  |  |  | 100% |  |  | coding (7‑67/1260 nt) | *hflK* → | modulator for HflB protease specific for phage lambda cII repressor |
| position | mutation | ZDBp871\_minus\_CZB151 | ZDBp875\_minus\_CZB151 | ZDBp877\_minus\_CZB152 | ZDBp880\_minus\_CZB152 | ZDBp883\_minus\_CZB154 | ZDBp886\_minus\_CZB154 | ZDBp889\_minus\_ZDB67 | ZDBp892\_minus\_ZDB67 | ZDBp895\_minus\_ZDB68 | ZDBp898\_minus\_ZDB68 | ZDBp901\_minus\_ZDB69 | ZDBp904\_minus\_ZDB69 | annotation | gene | description |
| 4,343,095 | +T |  |  |  |  |  |  |  |  |  |  |  | 100% | intergenic (+1157/+28) | *fklB* → / ← *insK‑2* | FKBP‑type peptidyl‑prolyl cis‑trans isomerase (rotamase)/IS150 putative transposase |
| 4,343,098 | Δ1,446 bp | 100% | 100% |  |  |  |  | 100% |  |  |  |  |  | IS*150*‑mediated | *insK‑2*–*insJ‑2* | *insK‑2*, *insJ‑2* |
| 4,356,267 | C→T |  |  |  |  | 100% |  |  |  |  |  |  |  | N433N (AAC→AAT) | *ytfM* → | predicted outer membrane protein and surface antigen |
| 4,364,778 | IS*150* (+) +4 bp |  |  |  |  |  |  |  |  | 100% |  |  |  | coding (127‑130/1023 nt) | *ytfT* → | predicted sugar transporter subunit: membrane component of ABC superfamily |
| 4,446,620 | IS*150* (–) +3 bp |  |  |  |  |  |  |  |  | 100% |  |  |  | intergenic (‑60/+3) | *yjhS* ← / ← *yjhT* | hypothetical protein/hypothetical protein |
| 4,456,967 | IS*150* (–) +3 bp |  |  |  |  | 100% |  |  |  |  |  |  |  | intergenic (‑29/+19) | *yjiX* ← / ← *yjiY* | hypothetical protein/predicted inner membrane protein |
| 4,456,970 | IS*150* (–) +3 bp | 100% |  |  |  |  |  |  |  |  |  |  |  | intergenic (‑32/+16) | *yjiX* ← / ← *yjiY* | hypothetical protein/predicted inner membrane protein |
| 4,478,024 | IS*150* (–) +3 bp |  |  |  |  |  |  |  |  |  |  | 100% |  | coding (534‑536/2292 nt) | *mdoB* ← | phosphoglycerol transferase I |
| 4,478,040 | IS*150* (+) +3 bp |  |  |  |  |  |  |  | 100% |  | 100% |  |  | coding (518‑520/2292 nt) | *mdoB* ← | phosphoglycerol transferase I |
| 4,501,612 | C→T |  |  |  |  |  |  |  | 100% |  |  |  |  | E202K (GAG→AAG) | *lplA* ← | lipoate‑protein ligase A |
| position | mutation | ZDBp871\_minus\_CZB151 | ZDBp875\_minus\_CZB151 | ZDBp877\_minus\_CZB152 | ZDBp880\_minus\_CZB152 | ZDBp883\_minus\_CZB154 | ZDBp886\_minus\_CZB154 | ZDBp889\_minus\_ZDB67 | ZDBp892\_minus\_ZDB67 | ZDBp895\_minus\_ZDB68 | ZDBp898\_minus\_ZDB68 | ZDBp901\_minus\_ZDB69 | ZDBp904\_minus\_ZDB69 | annotation | gene | description |
| 4,502,903 | +A |  |  |  |  |  |  | 100% |  |  | 100% |  |  | intergenic (‑16/‑50) | *smp* ← / → *insJ‑2* | hypothetical protein/IS150 hypothetical protein |
