# Supplementary material for: Genomic and phenotypic evolution of Escherichia coli in a novel citrate-only resource environment
Source: eLife. 2020 May 29;9:e55414. doi: 10.7554/eLife.55414 (PMC7299349; doi:10.7554/eLife.55414)
Supplement: Supplementary file 5. [file elife-55414-supp5.zip › S4File_genomes-by-environment/DM25-genome-summary.html]

Mutation Comparison


| Predicted mutations | | | | | | | | | | | | | | | | |
| --- | --- | --- | --- | --- | --- | --- | --- | --- | --- | --- | --- | --- | --- | --- | --- | --- |
| position | mutation | ZDBp910\_minus\_CZB151 | ZDBp911\_minus\_CZB151 | ZDBp912\_minus\_CZB152 | ZDBp913\_minus\_CZB152 | ZDBp914\_minus\_CZB154 | ZDBp915\_minus\_CZB154 | ZDBp916\_minus\_ZDB67 | ZDBp917\_minus\_ZDB67 | ZDBp918\_minus\_ZDB68 | ZDBp919\_minus\_ZDB68 | ZDBp920\_minus\_ZDB69 | ZDBp921\_minus\_ZDB69 | annotation | gene | description |
| 16,990 | +A |  |  |  |  | 100% |  |  |  |  |  |  |  | intergenic (‑32/‑50) | *mokC* ← / → *insJ‑2* | regulatory protein for HokC, overlaps CDS of hokC/IS150 hypothetical protein |
| 300,764 | IS*150* (–) +3 bp |  |  |  | 100% |  |  |  |  |  |  |  |  | coding (577‑579/1671 nt) | *betA* ← | choline dehydrogenase |
| 319,673 | IS*150* (+) +3 bp |  |  |  |  |  | 100% |  |  |  |  |  |  | coding (174‑176/246 nt) | *yahM* → | hypothetical protein |
| 363,636 | G→A |  |  |  |  | 100% |  |  |  |  |  |  |  | K28K (AAG→AAA) | *yaiV* → | predicted DNA‑binding transcriptional regulator |
| 432,291 | 3 bp→ATT |  |  |  |  |  |  |  |  |  |  |  | 100% | intergenic (‑58/‑96) | *insL‑2* ← / → *lon* | putative transposase insL for insertion sequence IS186/DNA‑binding ATP‑dependent protease La |
| 432,296 | Δ1 bp |  |  |  |  |  |  |  |  |  |  |  | 100% | intergenic (‑63/‑93) | *insL‑2* ← / → *lon* | putative transposase insL for insertion sequence IS186/DNA‑binding ATP‑dependent protease La |
| 432,301 | Δ1 bp |  |  |  |  |  |  |  |  |  |  |  | 100% | intergenic (‑68/‑88) | *insL‑2* ← / → *lon* | putative transposase insL for insertion sequence IS186/DNA‑binding ATP‑dependent protease La |
| 432,359 | IS*150* (+) +4 bp |  |  |  |  |  |  |  |  | 100% |  |  |  | intergenic (‑126/‑27) | *insL‑2* ← / → *lon* | putative transposase insL for insertion sequence IS186/DNA‑binding ATP‑dependent protease La |
| 435,258 | IS*150* (+) Δ1 bp :: +TTC |  |  | 100% |  |  |  |  |  |  |  |  |  | intergenic (+33/‑159) | *hupB* → / → *ppiD* | HU, DNA‑binding transcriptional regulator, beta subunit/peptidyl‑prolyl cis‑trans isomerase (rotamase D) |
| 464,051 | IS*150* (+) +3 bp | 100% |  |  |  |  |  |  |  | 100% |  | 100% | 100% | coding (274‑276/528 nt) | *priC* ← | primosomal replication protein N'' |
| position | mutation | ZDBp910\_minus\_CZB151 | ZDBp911\_minus\_CZB151 | ZDBp912\_minus\_CZB152 | ZDBp913\_minus\_CZB152 | ZDBp914\_minus\_CZB154 | ZDBp915\_minus\_CZB154 | ZDBp916\_minus\_ZDB67 | ZDBp917\_minus\_ZDB67 | ZDBp918\_minus\_ZDB68 | ZDBp919\_minus\_ZDB68 | ZDBp920\_minus\_ZDB69 | ZDBp921\_minus\_ZDB69 | annotation | gene | description |
| 492,501 | IS*150* (–) +3 bp |  | ? | 100% | ? |  |  |  |  |  |  |  |  | coding (264‑266/810 nt) | *ybbO* ← | short chain dehydrogenase |
| 492,684 | C→T |  | ? |  |  |  | 100% |  |  |  |  |  |  | G28D (GGC→GAC) | *ybbO* ← | short chain dehydrogenase |
| 544,481 | IS*3* (–) +5 bp :: +T |  |  |  |  | 100% |  |  |  |  |  |  |  | coding (250‑254/552 nt) | *ybcL* → | predicted kinase inhibitor |
| 544,593 | +TGA :: IS*3* (+) +3 bp |  |  | 100% |  |  |  |  |  |  |  |  |  | coding (362‑364/552 nt) | *ybcL* → | predicted kinase inhibitor |
| 546,183 | Δ42,947 bp |  |  |  |  |  |  |  |  |  |  |  | 100% | IS*1*‑mediated | *[ybcN]*–*insJ‑1* | **42 genes***[ybcN]*, *ninE*, *ybcO*, *rus*, *ylcG*, *insA‑10*, *insB‑10*, *insB‑6*, *insA‑6*, *ECB\_00510*, *nohB*, *ECB\_00512*, *ECB\_00513*, *ECB\_00514*, *ECB\_00515*, *ECB\_00516*, *ECB\_00517*, *appY*, *ompT*, *envY*, *ybcH*, *nfrA*, *ECB\_00524*, *yhhI*, *ECB\_00526*, *ECB\_00527*, *ECB\_00528*, *ECB\_00529*, *ECB\_00530*, *cusS*, *cusR*, *cusC*, *ylcC*, *cusB*, *cusA*, *pheP*, *ybdG*, *nfnB*, *ybdF*, *ybdJ*, *ybdK*, *insJ‑1* *[ybcN]*, *ninE*, *ybcO*, *rus*, *ylcG*, *insA‑10*, *insB‑10*, *insB‑6*, *insA‑6*, *ECB\_00510*, *nohB*, *ECB\_00512*, *ECB\_00513*, *ECB\_00514*, *ECB\_00515*, *ECB\_00516*, *ECB\_00517*, *appY*, *ompT*, *envY*, *ybcH*, *nfrA*, *ECB\_00524*, *yhhI*, *ECB\_00526*, *ECB\_00527*, *ECB\_00528*, *ECB\_00529*, *ECB\_00530*, *cusS*, *cusR*, *cusC*, *ylcC*, *cusB*, *cusA*, *pheP*, *ybdG*, *nfnB*, *ybdF*, *ybdJ*, *ybdK*, *insJ‑1* |
| 549,926 | Δ39,972 bp |  |  |  |  | 100% |  | 100% | 100% | ? |  | 100% |  | between IS*1* | *ECB\_00510*–*insA‑7* | **35 genes***ECB\_00510*, *nohB*, *ECB\_00512*, *ECB\_00513*, *ECB\_00514*, *ECB\_00515*, *ECB\_00516*, *ECB\_00517*, *appY*, *ompT*, *envY*, *ybcH*, *nfrA*, *ECB\_00524*, *yhhI*, *ECB\_00526*, *ECB\_00527*, *ECB\_00528*, *ECB\_00529*, *ECB\_00530*, *cusS*, *cusR*, *cusC*, *ylcC*, *cusB*, *cusA*, *pheP*, *ybdG*, *nfnB*, *ybdF*, *ybdJ*, *ybdK*, *insJ‑1*, *insB‑7*, *insA‑7* *ECB\_00510*, *nohB*, *ECB\_00512*, *ECB\_00513*, *ECB\_00514*, *ECB\_00515*, *ECB\_00516*, *ECB\_00517*, *appY*, *ompT*, *envY*, *ybcH*, *nfrA*, *ECB\_00524*, *yhhI*, *ECB\_00526*, *ECB\_00527*, *ECB\_00528*, *ECB\_00529*, *ECB\_00530*, *cusS*, *cusR*, *cusC*, *ylcC*, *cusB*, *cusA*, *pheP*, *ybdG*, *nfnB*, *ybdF*, *ybdJ*, *ybdK*, *insJ‑1*, *insB‑7*, *insA‑7* |
| 568,370 | Δ19,700 bp | 100% |  |  |  | Δ |  | Δ | Δ | ? |  | Δ | Δ | IS*150*‑mediated | *[ECB\_00524]*–*ybdK* | **19 genes***[ECB\_00524]*, *yhhI*, *ECB\_00526*, *ECB\_00527*, *ECB\_00528*, *ECB\_00529*, *ECB\_00530*, *cusS*, *cusR*, *cusC*, *ylcC*, *cusB*, *cusA*, *pheP*, *ybdG*, *nfnB*, *ybdF*, *ybdJ*, *ybdK* *[ECB\_00524]*, *yhhI*, *ECB\_00526*, *ECB\_00527*, *ECB\_00528*, *ECB\_00529*, *ECB\_00530*, *cusS*, *cusR*, *cusC*, *ylcC*, *cusB*, *cusA*, *pheP*, *ybdG*, *nfnB*, *ybdF*, *ybdJ*, *ybdK* |
| 573,826 | IS*3* (–) +5 bp :: +T | Δ | 100% | ? |  | Δ |  | Δ | Δ | ? |  | Δ | Δ | coding (1419‑1423/1449 nt) | *cusS* ← | sensory histidine kinase in two‑component regulatory system with CusR, senses copper ions |
| 574,297 | +TGA :: IS*3* (+) +3 bp | Δ |  |  | 100% | Δ |  | Δ | Δ | ? |  | Δ | Δ | coding (950‑952/1449 nt) | *cusS* ← | sensory histidine kinase in two‑component regulatory system with CusR, senses copper ions |
| 581,931 | Δ6,139 bp | Δ |  |  |  | Δ | 100% | Δ | Δ | ? |  | Δ | Δ | IS*150*‑mediated | *[cusA]*–*ybdK* | *[cusA]*, *pheP*, *ybdG*, *nfnB*, *ybdF*, *ybdJ*, *ybdK* |
| position | mutation | ZDBp910\_minus\_CZB151 | ZDBp911\_minus\_CZB151 | ZDBp912\_minus\_CZB152 | ZDBp913\_minus\_CZB152 | ZDBp914\_minus\_CZB154 | ZDBp915\_minus\_CZB154 | ZDBp916\_minus\_ZDB67 | ZDBp917\_minus\_ZDB67 | ZDBp918\_minus\_ZDB68 | ZDBp919\_minus\_ZDB68 | ZDBp920\_minus\_ZDB69 | ZDBp921\_minus\_ZDB69 | annotation | gene | description |
| 588,070 | Δ1 bp | ? |  |  | 100% | Δ | ? | Δ | Δ | ? |  | Δ | Δ | intergenic (‑222/‑47) | *ybdK* ← / → *insJ‑1* | gamma‑glutamyl:cysteine ligase/IS150 hypothetical protein |
| 600,001 | IS*150* (–) +3 bp |  |  |  |  |  | 100% |  |  | ? |  |  |  | coding (3562‑3564/3882 nt) | *entF* → | enterobactin synthase multienzyme complex component, ATP‑dependent |
| 642,823 | Δ117 bp |  |  |  |  |  | 100% |  |  |  |  |  |  | IS*150*‑mediated | *lipA* ← / → *insJ‑2* | lipoyl synthase/IS150 hypothetical protein |
| 642,939 | Δ1 bp |  |  |  |  |  | Δ |  |  |  |  | 100% |  | intergenic (‑565/‑48) | *lipA* ← / → *insJ‑2* | lipoyl synthase/IS150 hypothetical protein |
| 665,705 | Δ1 bp |  |  |  |  |  |  |  | 100% |  |  |  |  | intergenic (‑486/‑51) | *rihA* ← / → *insJ‑2* | ribonucleoside hydrolase 1/IS150 hypothetical protein |
| 665,708 | Δ2 bp |  |  | 100% |  |  | 100% |  |  |  |  | 100% |  | intergenic (‑489/‑47) | *rihA* ← / → *insJ‑2* | ribonucleoside hydrolase 1/IS150 hypothetical protein |
| 665,709 | Δ1 bp | 100% |  | Δ |  |  | Δ |  |  |  | 100% | Δ |  | intergenic (‑490/‑47) | *rihA* ← / → *insJ‑2* | ribonucleoside hydrolase 1/IS150 hypothetical protein |
| 734,998 | IS*1* (–) +9 bp |  |  |  |  |  | 100% |  |  |  |  |  |  | coding (1275‑1283/1284 nt) | *gltA* ← | citrate synthase |
| 735,392 | C→A |  |  |  |  |  |  |  |  |  |  | 100% |  | D297Y (GAT→TAT) | *gltA* ← | citrate synthase |
| 736,294 | A→G |  |  |  |  |  |  |  |  | 100% |  |  |  | intergenic (‑14/‑695) | *gltA* ← / → *sdhC* | citrate synthase/succinate dehydrogenase cytochrome b556 large membrane subunit |
| position | mutation | ZDBp910\_minus\_CZB151 | ZDBp911\_minus\_CZB151 | ZDBp912\_minus\_CZB152 | ZDBp913\_minus\_CZB152 | ZDBp914\_minus\_CZB154 | ZDBp915\_minus\_CZB154 | ZDBp916\_minus\_ZDB67 | ZDBp917\_minus\_ZDB67 | ZDBp918\_minus\_ZDB68 | ZDBp919\_minus\_ZDB68 | ZDBp920\_minus\_ZDB69 | ZDBp921\_minus\_ZDB69 | annotation | gene | description |
| 736,587 | IS*1* (–) +9 bp |  | 100% |  |  |  |  |  |  |  |  |  |  | intergenic (‑307/‑394) | *gltA* ← / → *sdhC* | citrate synthase/succinate dehydrogenase cytochrome b556 large membrane subunit |
| 736,633 | (GTTGA)1→2 |  |  |  |  |  |  |  |  |  |  |  | 100% | intergenic (‑353/‑356) | *gltA* ← / → *sdhC* | citrate synthase/succinate dehydrogenase cytochrome b556 large membrane subunit |
| 736,640 | IS*1* (–) +9 bp | 100% |  |  |  |  |  |  |  |  |  |  |  | intergenic (‑360/‑341) | *gltA* ← / → *sdhC* | citrate synthase/succinate dehydrogenase cytochrome b556 large membrane subunit |
| 736,642 | IS*1* (–) +7 bp |  |  |  |  | 100% |  |  |  |  |  |  |  | intergenic (‑362/‑341) | *gltA* ← / → *sdhC* | citrate synthase/succinate dehydrogenase cytochrome b556 large membrane subunit |
| 736,735 | T→C |  |  |  |  |  |  | 100% |  |  |  |  |  | intergenic (‑455/‑254) | *gltA* ← / → *sdhC* | citrate synthase/succinate dehydrogenase cytochrome b556 large membrane subunit |
| 736,739 | G→T |  |  |  |  |  |  |  | 100% |  |  |  |  | intergenic (‑459/‑250) | *gltA* ← / → *sdhC* | citrate synthase/succinate dehydrogenase cytochrome b556 large membrane subunit |
| 889,395 | G→T |  |  |  |  |  |  |  |  |  |  | 100% |  | intergenic (+68/‑85) | *ECB\_00825* → / → *ECB\_00826* | putative replication protein for prophage/conserved hypothetical protein |
| 972,660 | G→T |  |  |  |  |  |  |  |  |  | 100% |  |  | intergenic (‑80/+325) | *focA* ← / ← *ycaO* | formate transporter/hypothetical protein |
| 1,107,553 | IS*150* (+) +3 bp |  |  |  |  |  | 100% |  |  |  |  |  |  | coding (2041‑2043/2424 nt) | *ycdS* ← | predicted outer membrane protein |
| 1,137,050 | IS*150* (–) +3 bp |  |  |  |  |  |  |  |  |  |  | 100% |  | coding (29‑31/246 nt) | *dinI* ← | DNA damage‑inducible protein I |
| position | mutation | ZDBp910\_minus\_CZB151 | ZDBp911\_minus\_CZB151 | ZDBp912\_minus\_CZB152 | ZDBp913\_minus\_CZB152 | ZDBp914\_minus\_CZB154 | ZDBp915\_minus\_CZB154 | ZDBp916\_minus\_ZDB67 | ZDBp917\_minus\_ZDB67 | ZDBp918\_minus\_ZDB68 | ZDBp919\_minus\_ZDB68 | ZDBp920\_minus\_ZDB69 | ZDBp921\_minus\_ZDB69 | annotation | gene | description |
| 1,137,051 | IS*150* (+) +3 bp |  |  | 100% |  |  |  |  |  |  |  |  |  | coding (28‑30/246 nt) | *dinI* ← | DNA damage‑inducible protein I |
| 1,137,052 | IS*150* (+) +3 bp |  |  |  |  |  |  |  |  |  |  |  | 100% | coding (27‑29/246 nt) | *dinI* ← | DNA damage‑inducible protein I |
| 1,173,387 | IS*150* (+) +3 bp |  |  |  |  |  | 100% |  |  | 100% |  |  |  | intergenic (+223/‑70) | *ycfH* → / → *ptsG* | predicted metallodependent hydrolase/fused glucose‑specific PTS enzymes: IIB component/IIC component |
| 1,270,529 | Δ621 bp |  |  |  |  |  |  | 100% |  |  |  |  |  | IS*150*‑mediated | *ldrB* | ldrB |
| 1,271,135 | Δ15 bp |  |  | 100% |  |  |  | Δ |  |  |  |  |  | IS*150*‑mediated | *ldrB* ← / ← *insK‑2* | toxic polypeptide, small/IS150 putative transposase |
| 1,429,178 | A→G |  | 100% |  |  |  |  |  |  |  |  |  |  | intergenic (‑121/+807) | *ynaE* ← / ← *ynaF* | predicted DNA‑binding transcriptional regulator/stress‑induced protein, ATP‑binding protein |
| 1,447,820 | A→C |  |  |  |  |  | 100% |  |  |  |  |  |  | K285Q (AAG→CAG) | *insF‑2* → | IS3 element protein InsF |
| 1,455,172 | Δ774 bp |  |  |  |  |  |  |  | 100% |  |  |  |  | IS*150*‑mediated | *[ynbD]* | *[ynbD]* |
| 1,457,389 | Δ11,725 bp |  | 100% |  | 100% |  | 100% | 100% |  | 100% | 100% | 100% |  | between IS*150* | *hrpA*–*insJ‑2* | *hrpA*, *ydcF*, *aldA*, *gapC*, *insA‑12*, *insB‑12*, *cybB*, *ydcA*, *hokB*, *mokB*, *insK‑2*, *insJ‑2* |
| 1,457,392 | Δ1 bp | ? | Δ |  | Δ |  | Δ | Δ |  | Δ | Δ | Δ | 100% | intergenic (‑51/‑474) | *insJ‑2* ← / → *hrpA* | IS150 hypothetical protein/ATP‑dependent helicase |
| position | mutation | ZDBp910\_minus\_CZB151 | ZDBp911\_minus\_CZB151 | ZDBp912\_minus\_CZB152 | ZDBp913\_minus\_CZB152 | ZDBp914\_minus\_CZB154 | ZDBp915\_minus\_CZB154 | ZDBp916\_minus\_ZDB67 | ZDBp917\_minus\_ZDB67 | ZDBp918\_minus\_ZDB68 | ZDBp919\_minus\_ZDB68 | ZDBp920\_minus\_ZDB69 | ZDBp921\_minus\_ZDB69 | annotation | gene | description |
| 1,534,643 | G→A |  |  |  |  |  | 100% |  |  |  |  |  |  | intergenic (‑73/+61) | *adhP* ← / ← *sfcA* | alcohol dehydrogenase/malate dehydrogenase, (decarboxylating, NAD‑requiring) (malic enzyme) |
| 1,729,737 | T→A |  |  |  |  | 100% |  |  |  |  |  |  |  | intergenic (‑48/+702) | *insJ‑2* ← / ← *ydhZ* | IS150 hypothetical protein/hypothetical protein |
| 1,729,739 | +G |  |  |  |  |  |  | 100% |  |  |  |  |  | intergenic (‑50/+700) | *insJ‑2* ← / ← *ydhZ* | IS150 hypothetical protein/hypothetical protein |
| 1,729,741 | Δ1 bp |  |  |  |  |  |  |  |  |  |  | 100% |  | intergenic (‑52/+698) | *insJ‑2* ← / ← *ydhZ* | IS150 hypothetical protein/hypothetical protein |
| 1,887,034 | IS*150* (+) +3 bp |  |  |  | 100% |  |  |  |  |  |  |  |  | intergenic (‑3/‑154) | *yobG* ← / → *ECB\_01797* | hypothetical protein/hypothetical protein |
| 1,915,305 | (A)8→7 |  |  |  |  |  |  |  | 100% |  |  |  |  | coding (391/870 nt) | *yebK* → | predicted DNA‑binding transcriptional regulator |
| 1,968,587 | IS*150* (–) +3 bp | 100% |  |  |  |  |  |  |  |  |  |  |  | intergenic (+14/+46) | *tyrP* → / ← *yecA* | tyrosine transporter/conserved metal‑binding protein |
| 1,988,168 | IS*150* (–) +3 bp |  |  |  | 100% |  |  |  |  |  |  |  |  | coding (5620‑5622/7152 nt) | *yeeJ* → | adhesin |
| 2,043,155 | G→A |  |  |  |  | 100% |  |  |  |  |  |  |  | R700C (CGC→TGC) | *wzc* ← | protein‑tyrosine kinase |
| 2,062,617 | Δ374 bp |  |  |  | 100% |  |  |  |  |  |  | 100% |  | IS*150*‑mediated | *yegL* ← / ← *insK‑2* | hypothetical protein/IS150 putative transposase |
| position | mutation | ZDBp910\_minus\_CZB151 | ZDBp911\_minus\_CZB151 | ZDBp912\_minus\_CZB152 | ZDBp913\_minus\_CZB152 | ZDBp914\_minus\_CZB154 | ZDBp915\_minus\_CZB154 | ZDBp916\_minus\_ZDB67 | ZDBp917\_minus\_ZDB67 | ZDBp918\_minus\_ZDB68 | ZDBp919\_minus\_ZDB68 | ZDBp920\_minus\_ZDB69 | ZDBp921\_minus\_ZDB69 | annotation | gene | description |
| 2,099,889 | IS*150* (–) +3 bp |  |  |  | 100% |  |  |  |  |  |  |  |  | coding (991‑993/2280 nt) | *yehM* → | hypothetical protein |
| 2,112,669 | IS*150* (–) +3 bp | 100% |  |  |  |  |  |  |  |  |  |  |  | coding (16‑18/918 nt) | *yehZ* ← | predicted transporter subunit: periplasmic‑binding component of ABC superfamily |
| 2,112,669 | IS*150* (+) +3 bp |  |  |  |  |  |  |  |  |  |  |  | 100% | coding (16‑18/918 nt) | *yehZ* ← | predicted transporter subunit: periplasmic‑binding component of ABC superfamily |
| 2,132,618 | IS*1* (+) +9 bp |  | 100% |  |  |  |  |  |  |  |  |  |  | coding (969‑977/999 nt) | *mglB* ← | methyl‑galactoside transporter subunit |
| 2,132,724 | IS*150* (+) +3 bp |  |  |  |  | 100% |  |  |  |  |  |  |  | coding (869‑871/999 nt) | *mglB* ← | methyl‑galactoside transporter subunit |
| 2,133,568 | IS*150* (+) +3 bp |  |  |  |  |  |  | 100% |  |  |  |  |  | coding (25‑27/999 nt) | *mglB* ← | methyl‑galactoside transporter subunit |
| 2,133,584 | T→G |  |  |  |  |  |  |  |  |  | 100% |  |  | K4T (AAG→ACG) | *mglB* ← | methyl‑galactoside transporter subunit |
| 2,250,174 | C→T |  |  |  |  |  |  |  |  |  |  |  | 100% | G187S (GGC→AGC) | *yfaX* ← | predicted DNA‑binding transcriptional regulator |
| 2,264,346 | IS*186* (+) +8 bp |  |  | 100% |  |  |  |  |  |  | 100% |  |  | coding (131‑138/963 nt) | *menC* ← | O‑succinylbenzoate synthase |
| 2,264,348 | IS*186* (–) +8 bp | 100% | 100% | 100% |  |  | 100% |  |  |  |  |  |  | coding (129‑136/963 nt) | *menC* ← | O‑succinylbenzoate synthase |
| position | mutation | ZDBp910\_minus\_CZB151 | ZDBp911\_minus\_CZB151 | ZDBp912\_minus\_CZB152 | ZDBp913\_minus\_CZB152 | ZDBp914\_minus\_CZB154 | ZDBp915\_minus\_CZB154 | ZDBp916\_minus\_ZDB67 | ZDBp917\_minus\_ZDB67 | ZDBp918\_minus\_ZDB68 | ZDBp919\_minus\_ZDB68 | ZDBp920\_minus\_ZDB69 | ZDBp921\_minus\_ZDB69 | annotation | gene | description |
| 2,465,970 | IS*186* (–) +9 bp | 100% |  |  |  |  |  |  |  |  |  |  |  | coding (584‑592/618 nt) | *hyfA* → | hydrogenase 4, 4Fe‑4S subunit |
| 2,466,822 | IS*150* (+) +4 bp | 100% |  |  |  |  |  |  |  |  |  |  |  | coding (819‑822/2019 nt) | *hyfB* → | NADH dehydrogenase subunit N |
| 2,544,924 | C→T |  |  |  |  |  |  |  |  |  |  | 100% |  | E627K (GAG→AAG) | *yphG* ← | hypothetical protein |
| 2,553,002 | IS*150* (–) +3 bp |  |  | 100% |  |  |  |  |  |  |  |  |  | coding (560‑562/714 nt) | *yfhG* ← | hypothetical protein |
| 2,600,587 | +T |  |  |  |  |  | 100% |  | 100% |  |  |  |  | intergenic (‑51/+655) | *insJ‑2* ← / ← *rluD* | IS150 hypothetical protein/23S rRNA pseudouridine synthase |
| 2,659,020 | IS*3* (–) +3 bp :: +TCA |  |  |  | 100% |  |  |  |  |  | 100% |  |  | intergenic (‑212/+21) | *csrA* ← / ← *alaS* | carbon storage regulator/alanyl‑tRNA synthetase |
| 2,694,135 | IS*150* (+) +3 bp |  |  |  |  | 100% |  |  |  |  |  |  |  | coding (135‑137/2079 nt) | *fhlA* → | DNA‑binding transcriptional activator |
| 2,694,867 | +T :: IS*150* (+) +3 bp |  |  |  |  |  |  | 100% |  |  |  |  |  | coding (867‑869/2079 nt) | *fhlA* → | DNA‑binding transcriptional activator |
| 2,812,156 | IS*150* (–) +3 bp |  |  | 100% |  |  |  |  |  |  |  |  |  | coding (1128‑1130/1194 nt) | *ygeD* ← | predicted inner membrane protein |
| 2,820,985 | IS*150* (–) +3 bp |  |  |  |  |  | 100% |  |  |  |  |  |  | coding (620‑622/1419 nt) | *araE* ← | arabinose transporter |
| position | mutation | ZDBp910\_minus\_CZB151 | ZDBp911\_minus\_CZB151 | ZDBp912\_minus\_CZB152 | ZDBp913\_minus\_CZB152 | ZDBp914\_minus\_CZB154 | ZDBp915\_minus\_CZB154 | ZDBp916\_minus\_ZDB67 | ZDBp917\_minus\_ZDB67 | ZDBp918\_minus\_ZDB68 | ZDBp919\_minus\_ZDB68 | ZDBp920\_minus\_ZDB69 | ZDBp921\_minus\_ZDB69 | annotation | gene | description |
| 2,844,380 | Δ41 bp |  |  |  |  |  |  | 100% |  |  |  |  |  | IS*150*‑mediated | *yqeA* → / ← *insK‑2* | predicted amino acid kinase/IS150 putative transposase |
| 2,896,913 | IS*150* (+) +3 bp |  |  | 100% |  |  |  |  |  |  |  |  |  | coding (112‑114/1479 nt) | *ygfH* → | propionyl‑CoA:succinate‑CoA transferase |
| 2,896,927 | IS*150* (+) +3 bp |  |  |  | 100% |  |  |  |  |  |  |  |  | coding (126‑128/1479 nt) | *ygfH* → | propionyl‑CoA:succinate‑CoA transferase |
| 2,942,822 | Δ19,201 bp |  |  |  |  |  |  |  |  |  |  | 100% |  | IS*150*‑mediated | *[ECB\_02797]*–*ECB\_02815* | **18 genes***[ECB\_02797]*, *ECB\_02798*, *flu*, *yeeR*, *ECB\_02802*, *yafZ*, *ECB\_02804*, *yeeS*, *yeeT*, *yeeU*, *yeeV*, *yeeW*, *ECB\_02810*, *ECB\_02811*, *ECB\_02812*, *ECB\_02813*, *ECB\_02814*, *ECB\_02815* *[ECB\_02797]*, *ECB\_02798*, *flu*, *yeeR*, *ECB\_02802*, *yafZ*, *ECB\_02804*, *yeeS*, *yeeT*, *yeeU*, *yeeV*, *yeeW*, *ECB\_02810*, *ECB\_02811*, *ECB\_02812*, *ECB\_02813*, *ECB\_02814*, *ECB\_02815* |
| 2,976,836 | IS*150* (–) +3 bp |  |  |  | 100% |  |  |  |  |  |  |  |  | intergenic (‑322/+31) | *yghK* ← / ← *glcB* | glycolate transporter/malate synthase |
| 2,978,663 | IS*150* (+) +3 bp |  |  | 100% |  |  |  |  |  |  |  |  |  | coding (376‑378/2172 nt) | *glcB* ← | malate synthase |
| 3,109,394 | IS*150* (–) +3 bp | 100% |  |  | 100% |  |  |  |  |  |  | 100% | 100% | coding (245‑247/663 nt) | *yqjA* → | conserved inner membrane protein |
| 3,109,394 | IS*150* (–) +4 bp |  |  |  |  |  | 100% |  |  |  |  |  |  | coding (245‑248/663 nt) | *yqjA* → | conserved inner membrane protein |
| 3,172,540 | IS*150* (–) +3 bp | 100% |  |  |  |  |  |  |  |  |  |  |  | intergenic (‑39/+68) | *nlpI* ← / ← *pnp* | hypothetical protein/polynucleotide phosphorylase/polyadenylase |
| 3,362,135 | C→T |  |  | 100% |  |  |  |  |  |  |  |  |  | C132C (TGC→TGT) | *yhfR* → | predicted DNA‑binding transcriptional regulator |
| position | mutation | ZDBp910\_minus\_CZB151 | ZDBp911\_minus\_CZB151 | ZDBp912\_minus\_CZB152 | ZDBp913\_minus\_CZB152 | ZDBp914\_minus\_CZB154 | ZDBp915\_minus\_CZB154 | ZDBp916\_minus\_ZDB67 | ZDBp917\_minus\_ZDB67 | ZDBp918\_minus\_ZDB68 | ZDBp919\_minus\_ZDB68 | ZDBp920\_minus\_ZDB69 | ZDBp921\_minus\_ZDB69 | annotation | gene | description |
| 3,391,153 | T→C |  |  |  |  | 100% |  |  |  |  |  |  |  | intergenic (‑247/‑132) | *yhgE* ← / → *pckA* | predicted inner membrane protein/phosphoenolpyruvate carboxykinase |
| 3,391,209 | A→G |  |  |  |  |  |  | 100% |  |  |  |  |  | intergenic (‑303/‑76) | *yhgE* ← / → *pckA* | predicted inner membrane protein/phosphoenolpyruvate carboxykinase |
| 3,398,568 | IS*150* (+) +3 bp |  |  |  |  |  |  |  | 100% |  |  |  |  | intergenic (+395/‑41) | *yhgF* → / → *feoA* | predicted transcriptional accessory protein/ferrous iron transport protein A |
| 3,429,514 | A→C |  |  |  |  | 100% |  |  |  |  |  |  |  | F358C (TTC→TGC) | *glgC* ← | glucose‑1‑phosphate adenylyltransferase |
| 3,459,518 | (T)5→4 |  |  |  |  |  | 100% |  |  |  |  |  |  | intergenic (‑152/‑272) | *livK* ← / → *yhhK* | leucine transporter subunit/hypothetical protein |
| 3,466,937 | IS*150* (+) +4 bp |  |  | 100% |  |  |  |  |  |  |  |  |  | coding (139‑142/270 nt) | *yhhL* → | conserved inner membrane protein |
| 3,501,576 | IS*150* (–) +3 bp |  |  | 100% |  |  |  |  |  |  |  |  |  | intergenic (‑35/‑354) | *yhiO* ← / → *uspA* | universal stress protein UspB/universal stress global response regulator |
| 3,501,576 | IS*150* (+) +3 bp | 100% |  |  |  |  |  | 100% |  |  |  | 100% |  | intergenic (‑35/‑354) | *yhiO* ← / → *uspA* | universal stress protein UspB/universal stress global response regulator |
| 3,504,875 | T→A |  |  |  |  |  | 100% |  |  |  |  |  |  | H27L (CAC→CTC) | *yhiQ* ← | predicted SAM‑dependent methyltransferase |
| 3,524,609 | G→T |  |  |  |  | 100% |  |  |  |  |  |  |  | G211G (GGC→GGA) | *yhiW* ← | DNA‑binding transcriptional activator |
| position | mutation | ZDBp910\_minus\_CZB151 | ZDBp911\_minus\_CZB151 | ZDBp912\_minus\_CZB152 | ZDBp913\_minus\_CZB152 | ZDBp914\_minus\_CZB154 | ZDBp915\_minus\_CZB154 | ZDBp916\_minus\_ZDB67 | ZDBp917\_minus\_ZDB67 | ZDBp918\_minus\_ZDB68 | ZDBp919\_minus\_ZDB68 | ZDBp920\_minus\_ZDB69 | ZDBp921\_minus\_ZDB69 | annotation | gene | description |
| 3,574,917 | Δ7,306 bp | 100% |  |  |  |  |  |  |  |  |  |  |  | IS*150*‑mediated | *[tag]*–*hokA* | *[tag]*, *yiaC*, *bisC*, *yiaD*, *tkrA*, *yiaF*, *yiaG*, *cspA*, *hokA* |
| 3,582,219 | +A | Δ |  |  |  | 100% |  |  |  |  |  |  |  | intergenic (‑29/‑51) | *hokA* ← / → *insJ‑4* | toxic polypeptide, small/IS150 hypothetical protein |
| 3,770,320 | G→C | 100% |  |  |  |  |  |  |  |  |  |  |  | intergenic (‑93/+147) | *yidB* ← / ← *gyrB* | hypothetical protein/DNA gyrase subunit B |
| 3,770,422 | C→T |  |  |  |  |  |  |  |  |  |  | 100% |  | intergenic (‑195/+45) | *yidB* ← / ← *gyrB* | hypothetical protein/DNA gyrase subunit B |
| 3,825,950 | +GG |  |  |  |  |  |  |  |  |  | 100% |  |  | intergenic (+4/‑50) | *kup* → / → *insJ‑5* | potassium transporter/IS150 hypothetical protein |
| 3,827,398 | +C |  |  |  |  |  |  |  |  |  | 100% |  |  | intergenic (+29/+983) | *insK‑5* → / ← *yieP* | IS150 putative transposase/predicted transcriptional regulator |
| 3,857,836 | IS*150* (+) +3 bp |  |  |  |  |  | 100% |  |  |  |  |  |  | intergenic (+21/‑33) | *wzzE* → / → *wecB* | Entobacterial Common Antigen (ECA) polysaccharide chain length modulation protein/UDP‑N‑acetyl glucosamine‑2‑epimerase |
| 3,993,788 | IS*150* (+) +3 bp |  |  |  |  |  | 100% |  |  |  |  |  |  | coding (151‑153/1749 nt) | *frvR* ← | predicted regulator |
| 4,022,848 | Δ7 bp |  |  | 100% |  |  |  |  |  |  |  |  |  | coding (657‑663/846 nt) | *glpF* ← | glycerol facilitator |
| 4,091,094 | A→G |  |  |  |  |  |  |  | 100% |  |  |  |  | Q952R (CAG→CGG) | *rpoB* → | DNA‑directed RNA polymerase subunit beta |
| position | mutation | ZDBp910\_minus\_CZB151 | ZDBp911\_minus\_CZB151 | ZDBp912\_minus\_CZB152 | ZDBp913\_minus\_CZB152 | ZDBp914\_minus\_CZB154 | ZDBp915\_minus\_CZB154 | ZDBp916\_minus\_ZDB67 | ZDBp917\_minus\_ZDB67 | ZDBp918\_minus\_ZDB68 | ZDBp919\_minus\_ZDB68 | ZDBp920\_minus\_ZDB69 | ZDBp921\_minus\_ZDB69 | annotation | gene | description |
| 4,091,159 | C→T |  |  |  | 100% |  |  |  |  |  |  |  |  | R974C (CGT→TGT) | *rpoB* → | DNA‑directed RNA polymerase subunit beta |
| 4,095,055 | C→T |  |  | 100% |  |  |  |  |  |  |  |  |  | A904V (GCG→GTG) | *rpoC* → | DNA‑directed RNA polymerase subunit beta' |
| 4,101,561 | IS*150* (–) +3 bp |  |  |  |  |  |  |  |  |  |  |  | 100% | coding (1532‑1534/1896 nt) | *thiC* ← | thiamine biosynthesis protein ThiC |
| 4,122,222 | IS*1* (+) +9 bp |  |  |  |  |  |  |  |  | 100% |  |  |  | intergenic (+184/‑77) | *metA* → / → *aceB* | homoserine O‑succinyltransferase/malate synthase |
| 4,122,388 | Δ4 bp |  |  |  |  | 100% |  |  |  |  |  |  |  | coding (82‑85/1602 nt) | *aceB* → | malate synthase |
| 4,122,539 | G→A |  |  |  |  |  |  |  |  |  | 100% |  |  | W78\* (TGG→TAG) | *aceB* → | malate synthase |
| 4,122,799 | Δ1 bp |  |  |  |  |  |  |  |  |  |  | 100% |  | coding (493/1602 nt) | *aceB* → | malate synthase |
| 4,122,888 | IS*150* (–) +3 bp |  |  |  | 100% |  |  |  |  |  |  |  |  | coding (582‑584/1602 nt) | *aceB* → | malate synthase |
| 4,122,957 | IS*150* (–) +4 bp |  |  |  |  |  |  |  | 100% |  |  |  |  | coding (651‑654/1602 nt) | *aceB* → | malate synthase |
| 4,123,868 | IS*150* (–) +3 bp | 100% | 100% |  |  |  |  |  |  |  |  |  |  | coding (1562‑1564/1602 nt) | *aceB* → | malate synthase |
| position | mutation | ZDBp910\_minus\_CZB151 | ZDBp911\_minus\_CZB151 | ZDBp912\_minus\_CZB152 | ZDBp913\_minus\_CZB152 | ZDBp914\_minus\_CZB154 | ZDBp915\_minus\_CZB154 | ZDBp916\_minus\_ZDB67 | ZDBp917\_minus\_ZDB67 | ZDBp918\_minus\_ZDB68 | ZDBp919\_minus\_ZDB68 | ZDBp920\_minus\_ZDB69 | ZDBp921\_minus\_ZDB69 | annotation | gene | description |
| 4,123,908 | IS*1* (+) +9 bp |  |  |  |  |  |  | 100% |  |  |  |  |  |  | *[aceB]* | *[aceB]* |
| 4,124,014 | Δ2 bp |  |  |  |  |  |  |  |  |  |  |  | 100% | coding (77‑78/1305 nt) | *aceA* → | isocitrate lyase |
| 4,134,124 | C→T |  |  |  |  |  |  |  | 100% |  |  |  |  | R27C (CGC→TGC) | *yjbB* → | predicted transporter |
| 4,191,234 | IS*150* (+) +3 bp |  |  |  |  |  |  | 100% |  |  |  |  |  | coding (295‑297/315 nt) | *yjcH* ← | conserved inner membrane protein involved in acetate transport |
| 4,201,689 | IS*150* (–) +3 bp |  |  |  |  |  |  |  |  | 100% |  |  |  | coding (892‑894/1314 nt) | *gltP* → | glutamate/aspartate:proton symporter |
| 4,256,901 | (CGCGG)3→2 |  |  |  |  | 100% |  |  |  |  |  | 100% | 100% | intergenic (‑768/‑1042) | *dcuR* ← / → *yjdI* | DNA‑binding response regulator in two‑component regulatory system with DcuS/hypothetical protein |
| 4,342,160 | +T |  |  | 100% |  |  |  |  |  |  |  |  |  | intergenic (+222/+963) | *fklB* → / ← *insK‑2* | FKBP‑type peptidyl‑prolyl cis‑trans isomerase (rotamase)/IS150 putative transposase |
| 4,343,098 | Δ1,446 bp |  |  |  |  |  | ? |  |  |  | 100% |  |  | IS*150*‑mediated | *insK‑2*–*insJ‑2* | *insK‑2*, *insJ‑2* |
| 4,403,966 | IS*150* (+) +3 bp |  |  |  |  |  |  |  |  |  |  |  | 100% | coding (613‑615/1320 nt) | *idnT* ← | L‑idonate and D‑gluconate transporter |
| 4,456,970 | IS*150* (–) +3 bp |  | 100% |  |  |  |  |  |  |  |  |  |  | intergenic (‑32/+16) | *yjiX* ← / ← *yjiY* | hypothetical protein/predicted inner membrane protein |
| position | mutation | ZDBp910\_minus\_CZB151 | ZDBp911\_minus\_CZB151 | ZDBp912\_minus\_CZB152 | ZDBp913\_minus\_CZB152 | ZDBp914\_minus\_CZB154 | ZDBp915\_minus\_CZB154 | ZDBp916\_minus\_ZDB67 | ZDBp917\_minus\_ZDB67 | ZDBp918\_minus\_ZDB68 | ZDBp919\_minus\_ZDB68 | ZDBp920\_minus\_ZDB69 | ZDBp921\_minus\_ZDB69 | annotation | gene | description |
| 4,456,970 | +AAG :: IS*150* (+) +2 bp |  |  |  |  |  |  |  |  |  | 100% |  |  | intergenic (‑32/+17) | *yjiX* ← / ← *yjiY* | hypothetical protein/predicted inner membrane protein |
| 4,478,024 | IS*150* (–) +3 bp |  |  |  |  |  | 100% |  |  |  |  |  |  | coding (534‑536/2292 nt) | *mdoB* ← | phosphoglycerol transferase I |
| 4,502,903 | +A |  |  |  |  |  |  |  | 100% |  |  |  |  | intergenic (‑16/‑50) | *smp* ← / → *insJ‑2* | hypothetical protein/IS150 hypothetical protein |
