# Supplementary material for: Genomic and phenotypic evolution of Escherichia coli in a novel citrate-only resource environment
Source: eLife. 2020 May 29;9:e55414. doi: 10.7554/eLife.55414 (PMC7299349; doi:10.7554/eLife.55414)
Supplement: Supplementary file 5. [file elife-55414-supp5.zip › S4File_genomes-by-environment/DM0-html/ZDBp895_minus_ZDB68.hgml]

Mutation Comparison


| Predicted mutations | | | | |
| --- | --- | --- | --- | --- |
| position | mutation | annotation | gene | description |
| 248,532 | IS*150* (–) +4 bp | coding (786‑789/2445 nt) | *fadE* ← | acyl‑CoA dehydrogenase |
| 735,797 | C→T | A162T (GCG→ACG) | *gltA* ← | citrate synthase |
| 891,825 | +TGA :: IS*3* (+) +3 bp | coding (913‑915/924 nt) | *ECB\_00830* → | hypothetical protein |
| 1,004,905 | C→T | R64H (CGT→CAT) | *ompF* ← | outer membrane porin 1a (Ia;b;F) |
| 1,181,558 | C→T | intergenic (+283/‑125) | *ycfP* → / → *ndh* | hypothetical protein/respiratory NADH dehydrogenase 2/cupric reductase |
| 1,457,389 | Δ11,725 bp | between IS*150* | *hrpA*–*insJ‑2* | *hrpA*, *ydcF*, *aldA*, *gapC*, *insA‑12*, *insB‑12*, *cybB*, *ydcA*, *hokB*, *mokB*, *insK‑2*, *insJ‑2* |
| 1,528,089 | IS*150* (+) +4 bp | intergenic (‑33/‑253) | *yddG* ← / → *fdnG* | predicted methyl viologen efflux pump/formate dehydrogenase‑N, alpha subunit, nitrate‑inducible |
| 1,536,758 | +TGA :: IS*3* (+) +3 bp | intergenic (‑63/+37) | *rpsV* ← / ← *bdm* | 30S ribosomal subunit protein S22/biofilm‑dependent modulation protein |
| 1,595,625 | IS*150* (+) +3 bp | coding (71‑73/900 nt) | *eamA* ← | cysteine and O‑acetyl‑L‑serine efflux system |
| 1,651,206 | IS*150* (+) +3 bp | coding (860‑862/1035 nt) | *ydgG* → | predicted inner membrane protein |
| position | mutation | annotation | gene | description |
| 1,802,927 | IS*1* (+) +9 bp | coding (941‑949/969 nt) | *astE* ← | succinylglutamate desuccinylase |
| 1,867,822 | IS*150* (–) +3 bp | coding (188‑190/1686 nt) | *fadD* ← | acyl‑CoA synthase |
| 2,133,554 | IS*150* (+) +3 bp | coding (39‑41/999 nt) | *mglB* ← | methyl‑galactoside transporter subunit |
| 2,549,863 | C→T | intergenic (‑299/‑29) | *glyA* ← / → *hmpA* | serine hydroxymethyltransferase/fused nitric oxide dioxygenase/dihydropteridine reductase 2 |
| 3,110,349 | IS*3* (–) +3 bp :: +TCA | coding (4‑6/369 nt) | *yqjC* → | hypothetical protein |
| 3,501,576 | IS*150* (+) +3 bp | intergenic (‑35/‑354) | *yhiO* ← / → *uspA* | universal stress protein UspB/universal stress global response regulator |
| 3,536,960 | IS*150* (–) +3 bp | coding (2028‑2030/2076 nt) | *yhjG* ← | predicted outer membrane biogenesis protein |
| 3,888,290 | IS*150* (–) +3 bp | coding (586‑588/903 nt) | *ECB\_03690* ← | conserved hypothetical protein |
| 4,364,778 | IS*150* (+) +4 bp | coding (127‑130/1023 nt) | *ytfT* → | predicted sugar transporter subunit: membrane component of ABC superfamily |
| 4,446,620 | IS*150* (–) +3 bp | intergenic (‑60/+3) | *yjhS* ← / ← *yjhT* | hypothetical protein/hypothetical protein |
