# Supplementary material for: Genomic and phenotypic evolution of Escherichia coli in a novel citrate-only resource environment
Source: eLife. 2020 May 29;9:e55414. doi: 10.7554/eLife.55414 (PMC7299349; doi:10.7554/eLife.55414)
Supplement: Supplementary file 5. [file elife-55414-supp5.zip › S4File_genomes-by-environment/DM0-html/ZDBp898_minus_ZDB68.html]

Mutation Comparison


| Predicted mutations | | | | |
| --- | --- | --- | --- | --- |
| position | mutation | annotation | gene | description |
| 140,966 | IS*150* (–) +4 bp | coding (198‑201/348 nt) | *yacC* ← | hypothetical protein |
| 468,912 | IS*150* (+) +3 bp | coding (279‑281/1875 nt) | *htpG* → | heat shock protein 90 |
| 642,809 | Δ131 bp | IS*150*‑mediated | *lipA* ← / → *insJ‑2* | lipoyl synthase/IS150 hypothetical protein |
| 735,797 | C→T | A162T (GCG→ACG) | *gltA* ← | citrate synthase |
| 1,097,283 | IS*150* (–) +3 bp | coding (674‑676/1509 nt) | *putP* → | proline:sodium symporter |
| 1,181,558 | C→T | intergenic (+283/‑125) | *ycfP* → / → *ndh* | hypothetical protein/respiratory NADH dehydrogenase 2/cupric reductase |
| 1,257,394 | A→G | intergenic (‑271/+498) | *ycgV* ← / ← *ychF* | predicted adhesin/translation‑associated GTPase |
| 1,306,894 | IS*150* (–) +3 bp | coding (1277‑1279/1461 nt) | *cls* ← | cardiolipin synthetase |
| 1,457,389 | Δ13,493 bp | IS*150*‑mediated | *hrpA*–*[trg]* | *hrpA*, *ydcF*, *aldA*, *gapC*, *insA‑12*, *insB‑12*, *cybB*, *ydcA*, *hokB*, *mokB*, *insK‑2*, *insJ‑2*, *[trg]* |
| 1,729,737 | Δ2,150 bp | IS*150*‑mediated | *ydhZ*–*[pykF]* | *ydhZ*, *[pykF]* |
| position | mutation | annotation | gene | description |
| 1,789,698 | (T)6→5 | coding (399/795 nt) | *ydjO* ← | hypothetical protein |
| 1,867,855 | +CTT | coding (157/1686 nt) | *fadD* ← | acyl‑CoA synthase |
| 1,887,084 | IS*1* (+) +9 bp | intergenic (‑53/‑98) | *yobG* ← / → *ECB\_01797* | hypothetical protein/hypothetical protein |
| 1,913,929 | IS*150* (–) +3 bp | coding (647‑649/1476 nt) | *zwf* ← | glucose‑6‑phosphate 1‑dehydrogenase |
| 2,468,533 | A→C | intergenic (+511/‑549) | *hyfB* → / → *hyfD* | NADH dehydrogenase subunit N/hydrogenase 4 membrane subunit |
| 2,769,779 | G→A | R415\* (CGA→TGA) | *ECB\_02649* ← | protein similar to L‑ribulokinase AraB |
| 2,845,864 | Δ2,335 bp | IS*150*‑mediated | *yqeC*–*[ygfK]* | *yqeC*, *ygfJ*, *[ygfK]* |
| 3,393,071 | IS*150* (–) +3 bp | coding (1263‑1265/1353 nt) | *envZ* ← | osmolarity sensor protein |
| 3,429,542 | C→A | G349C (GGT→TGT) | *glgC* ← | glucose‑1‑phosphate adenylyltransferase |
| 3,583,667 | Δ1 bp | intergenic (+28/+251) | *insK‑4* → / ← *glyS* | IS150 putative transposase/glycyl‑tRNA synthetase subunit beta |
| position | mutation | annotation | gene | description |
| 4,191,720 | IS*150* (+) +4 bp | intergenic (‑190/+7) | *yjcH* ← / ← *acs* | conserved inner membrane protein involved in acetate transport/acetyl‑coenzyme A synthetase |
| 4,203,100 | IS*150* (–) +3 bp | coding (230‑232/690 nt) | *yjcO* ← | hypothetical protein |
| 4,307,583 | Δ61 bp | coding (7‑67/1260 nt) | *hflK* → | modulator for HflB protease specific for phage lambda cII repressor |
| 4,478,040 | IS*150* (+) +3 bp | coding (518‑520/2292 nt) | *mdoB* ← | phosphoglycerol transferase I |
| 4,502,903 | +A | intergenic (‑16/‑50) | *smp* ← / → *insJ‑2* | hypothetical protein/IS150 hypothetical protein |
