# Supplementary material for: Genomic and phenotypic evolution of Escherichia coli in a novel citrate-only resource environment
Source: eLife. 2020 May 29;9:e55414. doi: 10.7554/eLife.55414 (PMC7299349; doi:10.7554/eLife.55414)
Supplement: Supplementary file 5. [file elife-55414-supp5.zip › S4File_genomes-by-environment/DM0-html/ZDBp880_minus_CZB152.html]

Mutation Comparison


| Predicted mutations | | | | |
| --- | --- | --- | --- | --- |
| position | mutation | annotation | gene | description |
| 549,926 | Δ39,972 bp | between IS*1* | *ECB\_00510*–*insA‑7* | **35 genes***ECB\_00510*, *nohB*, *ECB\_00512*, *ECB\_00513*, *ECB\_00514*, *ECB\_00515*, *ECB\_00516*, *ECB\_00517*, *appY*, *ompT*, *envY*, *ybcH*, *nfrA*, *ECB\_00524*, *yhhI*, *ECB\_00526*, *ECB\_00527*, *ECB\_00528*, *ECB\_00529*, *ECB\_00530*, *cusS*, *cusR*, *cusC*, *ylcC*, *cusB*, *cusA*, *pheP*, *ybdG*, *nfnB*, *ybdF*, *ybdJ*, *ybdK*, *insJ‑1*, *insB‑7*, *insA‑7* *ECB\_00510*, *nohB*, *ECB\_00512*, *ECB\_00513*, *ECB\_00514*, *ECB\_00515*, *ECB\_00516*, *ECB\_00517*, *appY*, *ompT*, *envY*, *ybcH*, *nfrA*, *ECB\_00524*, *yhhI*, *ECB\_00526*, *ECB\_00527*, *ECB\_00528*, *ECB\_00529*, *ECB\_00530*, *cusS*, *cusR*, *cusC*, *ylcC*, *cusB*, *cusA*, *pheP*, *ybdG*, *nfnB*, *ybdF*, *ybdJ*, *ybdK*, *insJ‑1*, *insB‑7*, *insA‑7* |
| 642,935 | Δ1 bp | intergenic (‑561/‑52) | *lipA* ← / → *insJ‑2* | lipoyl synthase/IS150 hypothetical protein |
| 735,765 | C→A | M172I (ATG→ATT) | *gltA* ← | citrate synthase |
| 1,236,016 | IS*150* (+) +3 bp | intergenic (‑128/‑91) | *nhaB* ← / → *fadR* | sodium/proton antiporter/fatty acid metabolism regulator |
| 1,536,594 | +GA :: IS*3* (+) +4 bp | coding (99‑102/138 nt) | *rpsV* ← | 30S ribosomal subunit protein S22 |
| 2,133,567 | IS*150* (+) +4 bp | coding (25‑28/999 nt) | *mglB* ← | methyl‑galactoside transporter subunit |
| 2,325,035 | IS*150* (+) +3 bp | coding (685‑687/1179 nt) | *yfcJ* ← | predicted transporter |
| 3,196,333 | Δ59 bp | IS*150*‑mediated | *yhbE* ← / → *insJ‑2* | conserved inner membrane protein/IS150 hypothetical protein |
| 3,501,576 | IS*150* (+) +3 bp | intergenic (‑35/‑354) | *yhiO* ← / → *uspA* | universal stress protein UspB/universal stress global response regulator |
| 3,975,518 | IS*150* (–) +3 bp | coding (322‑324/1242 nt) | *yihS* ← | predicted glucosamine isomerase |
