# Supplementary material for: Genomic and phenotypic evolution of Escherichia coli in a novel citrate-only resource environment
Source: eLife. 2020 May 29;9:e55414. doi: 10.7554/eLife.55414 (PMC7299349; doi:10.7554/eLife.55414)
Supplement: Supplementary file 5. [file elife-55414-supp5.zip › S4File_genomes-by-environment/DM0-html/ZDBp871_minus_CZB151.html]

Mutation Comparison


| Predicted mutations | | | | |
| --- | --- | --- | --- | --- |
| position | mutation | annotation | gene | description |
| 199,024 | IS*150* (+) +3 bp | intergenic (+19/‑165) | *ispC* → / → *uppS* | 1‑deoxy‑D‑xylulose 5‑phosphate reductoisomerase/undecaprenyl pyrophosphate synthase |
| 547,546 | Δ103 bp | IS*1*‑mediated | *ylcG* → / → *insA‑10* | predicted protein/IS1 protein InsA |
| 549,926 | Δ39,972 bp | between IS*1* | *ECB\_00510*–*insA‑7* | **35 genes***ECB\_00510*, *nohB*, *ECB\_00512*, *ECB\_00513*, *ECB\_00514*, *ECB\_00515*, *ECB\_00516*, *ECB\_00517*, *appY*, *ompT*, *envY*, *ybcH*, *nfrA*, *ECB\_00524*, *yhhI*, *ECB\_00526*, *ECB\_00527*, *ECB\_00528*, *ECB\_00529*, *ECB\_00530*, *cusS*, *cusR*, *cusC*, *ylcC*, *cusB*, *cusA*, *pheP*, *ybdG*, *nfnB*, *ybdF*, *ybdJ*, *ybdK*, *insJ‑1*, *insB‑7*, *insA‑7* *ECB\_00510*, *nohB*, *ECB\_00512*, *ECB\_00513*, *ECB\_00514*, *ECB\_00515*, *ECB\_00516*, *ECB\_00517*, *appY*, *ompT*, *envY*, *ybcH*, *nfrA*, *ECB\_00524*, *yhhI*, *ECB\_00526*, *ECB\_00527*, *ECB\_00528*, *ECB\_00529*, *ECB\_00530*, *cusS*, *cusR*, *cusC*, *ylcC*, *cusB*, *cusA*, *pheP*, *ybdG*, *nfnB*, *ybdF*, *ybdJ*, *ybdK*, *insJ‑1*, *insB‑7*, *insA‑7* |
| 599,136 | IS*RSO11* (+) +3 bp | coding (2697‑2699/3882 nt) | *entF* → | enterobactin synthase multienzyme complex component, ATP‑dependent |
| 736,323 | T→G | intergenic (‑43/‑666) | *gltA* ← / → *sdhC* | citrate synthase/succinate dehydrogenase cytochrome b556 large membrane subunit |
| 1,134,456 | IS*150* (–) +3 bp | coding (179‑181/567 nt) | *yceJ* ← | predicted cytochrome b561 |
| 1,154,915 | IS*150* (+) +3 bp | coding (948‑950/1644 nt) | *flgK* → | flagellar hook‑associated protein K |
| 1,362,312 | Δ1 bp | coding (101/558 nt) | *ycjC* → | DNA‑binding transcriptional repressor |
| 1,457,389 | Δ11,725 bp | between IS*150* | *hrpA*–*insJ‑2* | *hrpA*, *ydcF*, *aldA*, *gapC*, *insA‑12*, *insB‑12*, *cybB*, *ydcA*, *hokB*, *mokB*, *insK‑2*, *insJ‑2* |
| 1,651,190 | IS*150* (–) +3 bp | coding (844‑846/1035 nt) | *ydgG* → | predicted inner membrane protein |
| position | mutation | annotation | gene | description |
| 1,776,123 | +T | intergenic (‑50/+57) | *insJ‑2* ← / ← *pheM* | IS150 hypothetical protein/phenylalanyl‑tRNA synthetase operon leader peptide |
| 1,897,614 | IS*150* (–) +3 bp | coding (1841‑1843/2634 nt) | *yebT* → | hypothetical protein |
| 2,209,801 | A→T | S351C (AGT→TGT) | *atoS* → | sensory histidine kinase in two‑component regulatory system with AtoC |
| 2,348,062 | IS*3* (–) +3 bp :: +TCA | coding (1090‑1092/1347 nt) | *fadL* → | long‑chain fatty acid outer membrane transporter |
| 2,535,008 | IS*150* (+) +1 bp :: +AA | coding (44/321 nt) | *hcaC* → | 3‑phenylpropionate dioxygenase, predicted ferredoxin subunit |
| 2,600,587 | +T | intergenic (‑51/+655) | *insJ‑2* ← / ← *rluD* | IS150 hypothetical protein/23S rRNA pseudouridine synthase |
| 2,650,572 | G→T | M47I (ATG→ATT) | *emrR* → | DNA‑binding transcriptional repressor of microcin B17 synthesis and multidrug efflux |
| 2,897,655 | IS*1* (+) +8 bp | coding (854‑861/1479 nt) | *ygfH* → | propionyl‑CoA:succinate‑CoA transferase |
| 3,242,824 | A→C | I148L (ATT→CTT) | *argR* → | arginine repressor |
| 3,402,005 | IS*150* (–) +1 bp :: +ATC | coding (391/879 nt) | *yhgA* → | predicted transposase |
| position | mutation | annotation | gene | description |
| 3,501,576 | IS*150* (–) +3 bp | intergenic (‑35/‑354) | *yhiO* ← / → *uspA* | universal stress protein UspB/universal stress global response regulator |
| 3,561,766 | IS*150* (–) +3 bp | intergenic (‑89/‑385) | *ldrD* ← / → *yhjV* | toxic polypeptide, small/predicted transporter |
| 3,756,769 | IS*1* (–) +8 bp | intergenic (‑325/+598) | *glvBC* ← / ← *yidE* | arbutin specific enzyme IIBC component of PTS/hypothetical protein |
| 3,932,217 | IS*150* (+) +3 bp | coding (865‑867/1164 nt) | *fadA* ← | acetyl‑CoA acetyltransferase |
| 3,975,518 | IS*150* (+) +3 bp | coding (322‑324/1242 nt) | *yihS* ← | predicted glucosamine isomerase |
| 4,035,958 | Δ220 bp | IS*150*‑mediated | *metB* → / ← *insK‑2* | cystathionine gamma‑synthase/IS150 putative transposase |
| 4,091,274 | A→C | E1012A (GAA→GCA) | *rpoB* → | DNA‑directed RNA polymerase subunit beta |
| 4,260,611 | IS*150* (–) +4 bp | coding (208‑211/1518 nt) | *lysU* ← | lysine tRNA synthetase, inducible |
| 4,343,098 | Δ1,446 bp | IS*150*‑mediated | *insK‑2*–*insJ‑2* | *insK‑2*, *insJ‑2* |
| 4,456,970 | IS*150* (–) +3 bp | intergenic (‑32/+16) | *yjiX* ← / ← *yjiY* | hypothetical protein/predicted inner membrane protein |
