# Supplementary material for: Genomic and phenotypic evolution of Escherichia coli in a novel citrate-only resource environment
Source: eLife. 2020 May 29;9:e55414. doi: 10.7554/eLife.55414 (PMC7299349; doi:10.7554/eLife.55414)
Supplement: Supplementary file 5. [file elife-55414-supp5.zip › S4File_genomes-by-environment/DM0-html/ZDBp883_minus_CZB154.html]

Mutation Comparison


| Predicted mutations | | | | |
| --- | --- | --- | --- | --- |
| position | mutation | annotation | gene | description |
| 247,779 | IS*150* (–) +3 bp | coding (1540‑1542/2445 nt) | *fadE* ← | acyl‑CoA dehydrogenase |
| 579,159 | IS*150* (–) +3 bp | coding (1203‑1205/1224 nt) | *cusB* → | copper/silver efflux system, membrane fusion protein |
| 590,047 | Δ8,054 bp | IS*150*‑mediated | *hokE*–*[entF]* | *hokE*, *insL‑3*, *entD*, *fepA*, *fes*, *ybdZ*, *[entF]* |
| 719,392 | 2 bp→CA | intergenic (+986/‑613) | *ECB\_00664* → / → *ybfD* | hypothetical protein/hypothetical protein |
| 719,413 | G→T | intergenic (+1007/‑593) | *ECB\_00664* → / → *ybfD* | hypothetical protein/hypothetical protein |
| 735,104 | T→G | I393L (ATC→CTC) | *gltA* ← | citrate synthase |
| 1,457,389 | Δ11,725 bp | between IS*150* | *hrpA*–*insJ‑2* | *hrpA*, *ydcF*, *aldA*, *gapC*, *insA‑12*, *insB‑12*, *cybB*, *ydcA*, *hokB*, *mokB*, *insK‑2*, *insJ‑2* |
| 1,567,292 | IS*1* (+) +9 bp | intergenic (‑61/+265) | *ydeP* ← / ← *ydeQ* | predicted oxidoreductase/predicted fimbrial‑like adhesin protein |
| 2,099,889 | IS*150* (–) +3 bp | coding (991‑993/2280 nt) | *yehM* → | hypothetical protein |
| 2,209,853 | C→A | S368Y (TCT→TAT) | *atoS* → | sensory histidine kinase in two‑component regulatory system with AtoC |
| position | mutation | annotation | gene | description |
| 2,348,064 | IS*150* (+) +3 bp | coding (1092‑1094/1347 nt) | *fadL* → | long‑chain fatty acid outer membrane transporter |
| 2,526,007 | G→A | P61L (CCA→CTA) | *iscR* ← | DNA‑binding transcriptional repressor |
| 2,720,682 | Δ1 bp | intergenic (‑49/+200) | *insJ‑3* ← / ← *cysH* | IS150 hypothetical protein/phosphoadenosine phosphosulfate reductase |
| 3,109,394 | IS*150* (–) +4 bp | coding (245‑248/663 nt) | *yqjA* → | conserved inner membrane protein |
| 3,172,495 | Δ1 bp | coding (7/885 nt) | *nlpI* ← | hypothetical protein |
| 3,290,790 | Δ1 bp | coding (459/1125 nt) | *smf* ← | hypothetical protein |
| 3,501,576 | IS*150* (+) +3 bp :: +C | intergenic (‑35/‑354) | *yhiO* ← / → *uspA* | universal stress protein UspB/universal stress global response regulator |
| 3,687,738 | Δ1 bp :: IS*186* (+) +9 bp | coding (306‑314/864 nt) | *yicC* → | hypothetical protein |
| 3,825,949 | Δ1 bp | intergenic (+3/‑51) | *kup* → / → *insJ‑5* | potassium transporter/IS150 hypothetical protein |
| 4,356,267 | C→T | N433N (AAC→AAT) | *ytfM* → | predicted outer membrane protein and surface antigen |
| position | mutation | annotation | gene | description |
| 4,456,967 | IS*150* (–) +3 bp | intergenic (‑29/+19) | *yjiX* ← / ← *yjiY* | hypothetical protein/predicted inner membrane protein |
