# Supplementary material for: Genomic and phenotypic evolution of Escherichia coli in a novel citrate-only resource environment
Source: eLife. 2020 May 29;9:e55414. doi: 10.7554/eLife.55414 (PMC7299349; doi:10.7554/eLife.55414)
Supplement: Supplementary file 5. [file elife-55414-supp5.zip › S4File_genomes-by-environment/DM0-html/ZDBp875_minus_CZB151.html]

Mutation Comparison


| Predicted mutations | | | | |
| --- | --- | --- | --- | --- |
| position | mutation | annotation | gene | description |
| 574,068 | IS*150* (–) +3 bp | coding (1179‑1181/1449 nt) | *cusS* ← | sensory histidine kinase in two‑component regulatory system with CusR, senses copper ions |
| 590,047 | Δ2,297 bp | IS*150*‑mediated | *hokE*–*[entD]* | *hokE*, *insL‑3*, *[entD]* |
| 735,580 | G→T | A234D (GCC→GAC) | *gltA* ← | citrate synthase |
| 736,619 | T→C | intergenic (‑339/‑370) | *gltA* ← / → *sdhC* | citrate synthase/succinate dehydrogenase cytochrome b556 large membrane subunit |
| 923,149 | IS*150* (–) +3 bp | coding (509‑511/831 nt) | *ybjR* → | predicted amidase and lipoprotein |
| 1,004,636 | G→A | R154C (CGT→TGT) | *ompF* ← | outer membrane porin 1a (Ia;b;F) |
| 1,236,018 | IS*150* (–) +2 bp :: +TC | intergenic (‑130/‑90) | *nhaB* ← / → *fadR* | sodium/proton antiporter/fatty acid metabolism regulator |
| 1,294,964 | IS*3* (–) +4 bp :: +TC | intergenic (‑315/‑286) | *hns* ← / → *tdk* | global DNA‑binding transcriptional dual regulator H‑NS/thymidine kinase |
| 1,334,535 | A→C | M126L (ATG→CTG) | *cysB* → | DNA‑binding transcriptional dual regulator, O‑acetyl‑L‑serine‑binding |
| 1,457,389 | Δ14,145 bp | IS*150*‑mediated | *hrpA*–*[ydcI]* | *hrpA*, *ydcF*, *aldA*, *gapC*, *insA‑12*, *insB‑12*, *cybB*, *ydcA*, *hokB*, *mokB*, *insK‑2*, *insJ‑2*, *trg*, *[ydcI]* |
| position | mutation | annotation | gene | description |
| 1,640,803 | IS*150* (–) +3 bp | coding (761‑763/855 nt) | *ynfH* → | oxidoreductase, membrane subunit |
| 1,887,034 | IS*150* (+) +3 bp | intergenic (‑3/‑154) | *yobG* ← / → *ECB\_01797* | hypothetical protein/hypothetical protein |
| 1,902,189 | IS*150* (–) +3 bp | coding (664‑666/873 nt) | *yebZ* ← | predicted inner membrane protein |
| 2,302,013 | G→A | A556T (GCG→ACG) | *pta* → | phosphate acetyltransferase |
| 2,347,259 | IS*150* (–) +3 bp | coding (287‑289/1347 nt) | *fadL* → | long‑chain fatty acid outer membrane transporter |
| 2,963,466 | Δ13,009 bp | IS*150*‑mediated | *ECB\_02837*–*[yghK]* | *ECB\_02837*, *ECB\_02838*, *yghF*, *yghG*, *pppA*, *yghJ*, *[yghK]* |
| 3,501,576 | IS*150* (+) +3 bp | intergenic (‑35/‑354) | *yhiO* ← / → *uspA* | universal stress protein UspB/universal stress global response regulator |
| 3,975,518 | IS*150* (–) +3 bp | coding (322‑324/1242 nt) | *yihS* ← | predicted glucosamine isomerase |
| 4,188,718 | C→T | M225I (ATG→ATA) | *yjcF* ← | hypothetical protein |
| 4,260,612 | IS*150* (–) +3 bp | coding (208‑210/1518 nt) | *lysU* ← | lysine tRNA synthetase, inducible |
| position | mutation | annotation | gene | description |
| 4,343,098 | Δ1,446 bp | IS*150*‑mediated | *insK‑2*–*insJ‑2* | *insK‑2*, *insJ‑2* |
