# Supplementary material for: Genomic and phenotypic evolution of Escherichia coli in a novel citrate-only resource environment
Source: eLife. 2020 May 29;9:e55414. doi: 10.7554/eLife.55414 (PMC7299349; doi:10.7554/eLife.55414)
Supplement: Supplementary file 5. [file elife-55414-supp5.zip › S4File_genomes-by-environment/DM0-html/ZDBp886_minus_CZB154.html]

Mutation Comparison


| Predicted mutations | | | | |
| --- | --- | --- | --- | --- |
| position | mutation | annotation | gene | description |
| 243,649 | (G)8→7 | coding (193/372 nt) | *ECB\_00212* → | hypothetical protein |
| 572,890 | Δ16,240 bp | IS*1*‑mediated | *[ECB\_00530]*–*insJ‑1* | *[ECB\_00530]*, *cusS*, *cusR*, *cusC*, *ylcC*, *cusB*, *cusA*, *pheP*, *ybdG*, *nfnB*, *ybdF*, *ybdJ*, *ybdK*, *insJ‑1* |
| 736,126 | G→T | S52Y (TCC→TAC) | *gltA* ← | citrate synthase |
| 1,205,342 | T→G | T276P (ACC→CCC) | *phoQ* ← | sensory histidine kinase in two‑compoent regulatory system with PhoP |
| 1,619,073 | IS*150* (–) +3 bp | intergenic (‑71/+139) | *hokD* ← / ← *ECB\_01533* | small toxic polypeptide/conserved hypothetical protein |
| 1,651,192 | IS*150* (–) +3 bp :: +TACA | coding (846‑848/1035 nt) | *ydgG* → | predicted inner membrane protein |
| 2,079,174 | IS*150* (+) +3 bp | coding (613‑615/819 nt) | *yegX* ← | predicted hydrolase |
| 2,346,173 | G→A | intergenic (‑31/+150) | *yfcY* ← / ← *yfcZ* | acetyl‑CoA acetyltransferase/hypothetical protein |
| 2,348,272 | IS*150* (–) +3 bp | coding (1300‑1302/1347 nt) | *fadL* → | long‑chain fatty acid outer membrane transporter |
| 2,455,036 | A→G | intergenic (+142/‑397) | *acrD* → / → *yffB* | aminoglycoside/multidrug efflux system/hypothetical protein |
| position | mutation | annotation | gene | description |
| 2,957,887 | IS*150* (+) +3 bp | intergenic (‑78/‑494) | *ECB\_02812* ← / → *ECB\_02813* | conserved hypothetical protein/hypothetical protein |
| 3,109,394 | IS*150* (–) +3 bp | coding (245‑247/663 nt) | *yqjA* → | conserved inner membrane protein |
| 3,501,576 | IS*150* (+) +3 bp | intergenic (‑35/‑354) | *yhiO* ← / → *uspA* | universal stress protein UspB/universal stress global response regulator |
| 3,764,589 | IS*150* (+) +3 bp | intergenic (‑97/+21) | *dgoT* ← / ← *dgoD* | D‑galactonate transporter/galactonate dehydratase |
| 4,135,292 | A→C | D416A (GAT→GCT) | *yjbB* → | predicted transporter |
| 4,234,238 | IS*150* (+) +3 bp | coding (1006‑1008/2229 nt) | *yjdA* → | conserved protein with nucleoside triphosphate hydrolase domain |
