# Supplementary material for: Genomic and phenotypic evolution of Escherichia coli in a novel citrate-only resource environment
Source: eLife. 2020 May 29;9:e55414. doi: 10.7554/eLife.55414 (PMC7299349; doi:10.7554/eLife.55414)
Supplement: Supplementary file 5. [file elife-55414-supp5.zip › S4File_genomes-by-environment/DM0-html/ZDBp904_minus_ZDB69.html]

Mutation Comparison


| Predicted mutations | | | | |
| --- | --- | --- | --- | --- |
| position | mutation | annotation | gene | description |
| 237,985 | IS*150* (+) +3 bp | coding (339‑341/1359 nt) | *mltD* ← | predicted membrane‑bound lytic murein transglycosylase D |
| 734,968 | IS*1* (–) +9 bp | intergenic (‑361/+21) | *ybgD* ← / ← *gltA* | predicted fimbrial‑like adhesin protein/citrate synthase |
| 831,886 | C→T | A73T (GCT→ACT) | *ybiI* ← | hypothetical protein |
| 969,347 | IS*150* (–) +3 bp | intergenic (‑153/+37) | *pflA* ← / ← *pflB* | pyruvate formate lyase activating enzyme 1/pyruvate formate lyase I |
| 1,423,903 | Δ23,962 bp | between IS*3* | *ECB\_01341*–*[ydbC]* | **23 genes***ECB\_01341*, *ECB\_01342*, *ECB\_01343*, *ECB\_01344*, *pinR*, *ynaE*, *ynaF*, *ompN*, *insB‑10*, *insA‑10*, *ydbK*, *ydbJ*, *hslJ*, *ldhA*, *ydbH*, *ynbE*, *ydbL*, *feaR*, *feaB*, *tynA*, *insE‑2*, *insF‑2*, *[ydbC]* *ECB\_01341*, *ECB\_01342*, *ECB\_01343*, *ECB\_01344*, *pinR*, *ynaE*, *ynaF*, *ompN*, *insB‑10*, *insA‑10*, *ydbK*, *ydbJ*, *hslJ*, *ldhA*, *ydbH*, *ynbE*, *ydbL*, *feaR*, *feaB*, *tynA*, *insE‑2*, *insF‑2*, *[ydbC]* |
| 1,457,389 | Δ11,725 bp | between IS*150* | *hrpA*–*insJ‑2* | *hrpA*, *ydcF*, *aldA*, *gapC*, *insA‑12*, *insB‑12*, *cybB*, *ydcA*, *hokB*, *mokB*, *insK‑2*, *insJ‑2* |
| 1,821,169 | IS*150* (+) +3 bp | coding (616‑618/1041 nt) | *ynjI* ← | predicted inner membrane protein |
| 1,896,278 | IS*150* (–) +4 bp | coding (505‑508/2634 nt) | *yebT* → | hypothetical protein |
| 2,133,582 | IS*150* (+) +3 bp | coding (11‑13/999 nt) | *mglB* ← | methyl‑galactoside transporter subunit |
| 2,230,905 | IS*150* (+) +3 bp | coding (1971‑1973/3753 nt) | *yfaL* ← | adhesin |
| position | mutation | annotation | gene | description |
| 2,346,183 | G→A | intergenic (‑41/+140) | *yfcY* ← / ← *yfcZ* | acetyl‑CoA acetyltransferase/hypothetical protein |
| 2,525,967 | (CTAATTACTTCGCCAACGGCG)1→2 | coding (222/489 nt) | *iscR* ← | DNA‑binding transcriptional repressor |
| 3,501,576 | IS*150* (+) +3 bp | intergenic (‑35/‑354) | *yhiO* ← / → *uspA* | universal stress protein UspB/universal stress global response regulator |
| 4,343,095 | +T | intergenic (+1157/+28) | *fklB* → / ← *insK‑2* | FKBP‑type peptidyl‑prolyl cis‑trans isomerase (rotamase)/IS150 putative transposase |
