# Supplementary material for: Genomic and phenotypic evolution of Escherichia coli in a novel citrate-only resource environment
Source: eLife. 2020 May 29;9:e55414. doi: 10.7554/eLife.55414 (PMC7299349; doi:10.7554/eLife.55414)
Supplement: Supplementary file 5. [file elife-55414-supp5.zip › S4File_genomes-by-environment/DM0-html/ZDBp877_minus_CZB152.html]

Mutation Comparison


| Predicted mutations | | | | |
| --- | --- | --- | --- | --- |
| position | mutation | annotation | gene | description |
| 323,378 | G→A | D201N (GAT→AAT) | *prpB* → | 2‑methylisocitrate lyase |
| 557,465 | +TGA :: IS*3* (+) +3 bp | coding (542‑544/705 nt) | *ECB\_00515* → | conserved hypothetical protein |
| 735,765 | C→A | M172I (ATG→ATT) | *gltA* ← | citrate synthase |
| 940,328 | C→T | intergenic (‑11/‑312) | *cspD* ← / → *clpS* | cold shock protein homolog/ATP‑dependent Clp protease adaptor protein ClpS |
| 1,004,674 | C→A | G141V (GGC→GTC) | *ompF* ← | outer membrane porin 1a (Ia;b;F) |
| 1,137,052 | IS*150* (+) +3 bp | coding (27‑29/246 nt) | *dinI* ← | DNA damage‑inducible protein I |
| 1,181,558 | C→T | intergenic (+283/‑125) | *ycfP* → / → *ndh* | hypothetical protein/respiratory NADH dehydrogenase 2/cupric reductase |
| 1,307,420 | IS*150* (–) +3 bp | coding (751‑753/1461 nt) | *cls* ← | cardiolipin synthetase |
| 1,457,389 | Δ11,725 bp | between IS*150* | *hrpA*–*insJ‑2* | *hrpA*, *ydcF*, *aldA*, *gapC*, *insA‑12*, *insB‑12*, *cybB*, *ydcA*, *hokB*, *mokB*, *insK‑2*, *insJ‑2* |
| 1,887,041 | IS*1* (+) +9 bp | intergenic (‑10/‑141) | *yobG* ← / → *ECB\_01797* | hypothetical protein/hypothetical protein |
| position | mutation | annotation | gene | description |
| 1,988,179 | IS*150* (+) +3 bp | coding (5631‑5633/7152 nt) | *yeeJ* → | adhesin |
| 2,348,048 | IS*150* (–) +3 bp | coding (1076‑1078/1347 nt) | *fadL* → | long‑chain fatty acid outer membrane transporter |
| 2,465,970 | IS*186* (–) +8 bp | coding (584‑591/618 nt) | *hyfA* → | hydrogenase 4, 4Fe‑4S subunit |
| 2,527,313 | C→A | R23L (CGT→CTT) | *yfhQ* ← | predicted methyltransferase |
| 2,529,490 | +ACA | intergenic (+189/‑2) | *yfhR* → / → *csiE* | predicted peptidase/stationary phase inducible protein |
| 2,914,014 | IS*150* (+) +3 bp | coding (102‑104/759 nt) | *yggG* → | predicted peptidase |
| 3,172,540 | IS*150* (–) +3 bp | intergenic (‑39/+68) | *nlpI* ← / ← *pnp* | hypothetical protein/polynucleotide phosphorylase/polyadenylase |
| 3,501,576 | IS*150* (+) +3 bp | intergenic (‑35/‑354) | *yhiO* ← / → *uspA* | universal stress protein UspB/universal stress global response regulator |
| 3,544,110 | A→G | intergenic (‑39/+144) | *dctA* ← / ← *yhjK* | C4‑dicarboxylate transport protein/predicted diguanylate cyclase |
| 3,700,202 | IS*1* (–) +8 bp | intergenic (‑94/‑179) | *gltS* ← / → *yicE* | glutamate transporter/predicted transporter |
| position | mutation | annotation | gene | description |
| 3,748,889 | IS*150* (–) +3 bp | coding (142‑144/450 nt) | *yidI* → | predicted inner membrane protein |
