# Supplementary material for: Genomic and phenotypic evolution of Escherichia coli in a novel citrate-only resource environment
Source: eLife. 2020 May 29;9:e55414. doi: 10.7554/eLife.55414 (PMC7299349; doi:10.7554/eLife.55414)
Supplement: Supplementary file 5. [file elife-55414-supp5.zip › S4File_genomes-by-environment/DM0-html/ZDBp889_minus_ZDB67.html]

Mutation Comparison


| Predicted mutations | | | | |
| --- | --- | --- | --- | --- |
| position | mutation | annotation | gene | description |
| 435,255 | IS*150* (+) +3 bp | intergenic (+30/‑160) | *hupB* → / → *ppiD* | HU, DNA‑binding transcriptional regulator, beta subunit/peptidyl‑prolyl cis‑trans isomerase (rotamase D) |
| 590,047 | Δ2,134 bp | IS*150*‑mediated | *hokE*–*[entD]* | *hokE*, *insL‑3*, *[entD]* |
| 735,941 | T→A | I114F (ATC→TTC) | *gltA* ← | citrate synthase |
| 939,393 | Δ3,661 bp |  | *[macB]*–*[clpA]* | *[macB]*, *cspD*, *clpS*, *[clpA]* |
| 1,039,280 | IS*150* (–) +3 bp | coding (31‑33/630 nt) | *yccR* → | hypothetical protein |
| 1,064,767 | IS*150* (–) +3 bp | coding (1289‑1291/2097 nt) | *ymcA* ← | hypothetical protein |
| 1,154,915 | IS*150* (+) +3 bp | coding (948‑950/1644 nt) | *flgK* → | flagellar hook‑associated protein K |
| 1,236,016 | IS*150* (–) +3 bp | intergenic (‑128/‑91) | *nhaB* ← / → *fadR* | sodium/proton antiporter/fatty acid metabolism regulator |
| 1,306,724 | Δ1 bp | coding (1449/1461 nt) | *cls* ← | cardiolipin synthetase |
| 1,466,345 | Δ7,319 bp | IS*1*‑mediated | *cybB*–*ydcJ* | *cybB*, *ydcA*, *hokB*, *mokB*, *insK‑2*, *insJ‑2*, *trg*, *ydcI*, *ydcJ* |
| position | mutation | annotation | gene | description |
| 1,619,073 | IS*150* (–) +3 bp | intergenic (‑71/+139) | *hokD* ← / ← *ECB\_01533* | small toxic polypeptide/conserved hypothetical protein |
| 1,729,741 | Δ1 bp | intergenic (‑52/+698) | *insJ‑2* ← / ← *ydhZ* | IS150 hypothetical protein/hypothetical protein |
| 1,887,041 | IS*1* (–) +9 bp | intergenic (‑10/‑141) | *yobG* ← / → *ECB\_01797* | hypothetical protein/hypothetical protein |
| 2,348,272 | IS*150* (–) +3 bp | coding (1300‑1302/1347 nt) | *fadL* → | long‑chain fatty acid outer membrane transporter |
| 2,434,162 | G→T | S121R (AGC→AGA) | *eutJ* ← | predicted chaperonin, ethanolamine utilization protein |
| 2,623,300 | IS*3* (–) +3 bp :: +TCA | coding (1081‑1083/1230 nt) | *ECB\_02509* → | Fels‑2 prophage protein |
| 2,630,053 | A→C | I197L (ATT→CTT) | *ygaF* → | predicted enzyme |
| 2,938,642 | IS*150* (–) +3 bp | coding (1019‑1021/1257 nt) | *nupG* → | nucleoside transporter |
| 3,501,577 | IS*150* (–) +2 bp | intergenic (‑36/‑354) | *yhiO* ← / → *uspA* | universal stress protein UspB/universal stress global response regulator |
| 3,538,891 | IS*150* (+) +3 bp | coding (97‑99/2076 nt) | *yhjG* ← | predicted outer membrane biogenesis protein |
| position | mutation | annotation | gene | description |
| 3,583,666 | Δ100 bp | IS*150*‑mediated | *insK‑4* → / ← *glyS* | IS150 putative transposase/glycyl‑tRNA synthetase subunit beta |
| 4,343,098 | Δ1,446 bp | IS*150*‑mediated | *insK‑2*–*insJ‑2* | *insK‑2*, *insJ‑2* |
| 4,502,903 | +A | intergenic (‑16/‑50) | *smp* ← / → *insJ‑2* | hypothetical protein/IS150 hypothetical protein |
