# Supplementary material for: Genomic and phenotypic evolution of Escherichia coli in a novel citrate-only resource environment
Source: eLife. 2020 May 29;9:e55414. doi: 10.7554/eLife.55414 (PMC7299349; doi:10.7554/eLife.55414)
Supplement: Supplementary file 5. [file elife-55414-supp5.zip › S4File_genomes-by-environment/DM0-html/ZDBp892_minus_ZDB67.html]

Mutation Comparison


| Predicted mutations | | | | |
| --- | --- | --- | --- | --- |
| position | mutation | annotation | gene | description |
| 464,051 | IS*150* (+) +3 bp | coding (274‑276/528 nt) | *priC* ← | primosomal replication protein N'' |
| 574,068 | IS*150* (–) +3 bp | coding (1179‑1181/1449 nt) | *cusS* ← | sensory histidine kinase in two‑component regulatory system with CusR, senses copper ions |
| 660,274 | Δ5,435 bp | IS*150*‑mediated | *ybeR*–*rihA* | *ybeR*, *ybeV*, *hscC*, *rihA* |
| 735,941 | T→A | I114F (ATC→TTC) | *gltA* ← | citrate synthase |
| 1,205,348 | C→A | D274Y (GAC→TAC) | *phoQ* ← | sensory histidine kinase in two‑compoent regulatory system with PhoP |
| 1,271,135 | Δ15 bp | IS*150*‑mediated | *ldrB* ← / ← *insK‑2* | toxic polypeptide, small/IS150 putative transposase |
| 1,457,389 | Δ11,725 bp | between IS*150* | *hrpA*–*insJ‑2* | *hrpA*, *ydcF*, *aldA*, *gapC*, *insA‑12*, *insB‑12*, *cybB*, *ydcA*, *hokB*, *mokB*, *insK‑2*, *insJ‑2* |
| 2,331,208 | IS*RSO11* (+) +4 bp | coding (775‑778/825 nt) | *mepA* ← | penicillin‑insensitive murein endopeptidase |
| 2,630,053 | A→C | I197L (ATT→CTT) | *ygaF* → | predicted enzyme |
| 2,663,500 | C→T | intergenic (‑69/+11) | *recA* ← / ← *ygaD* | recombinase A/competence damage‑inducible protein A |
| position | mutation | annotation | gene | description |
| 2,720,682 | Δ1 bp | intergenic (‑49/+200) | *insJ‑3* ← / ← *cysH* | IS150 hypothetical protein/phosphoadenosine phosphosulfate reductase |
| 3,109,394 | IS*150* (–) +3 bp | coding (245‑247/663 nt) | *yqjA* → | conserved inner membrane protein |
| 3,172,540 | IS*150* (–) +3 bp | intergenic (‑39/+68) | *nlpI* ← / ← *pnp* | hypothetical protein/polynucleotide phosphorylase/polyadenylase |
| 3,427,815 | T→C | N2D (AAT→GAT) | *glgP* ← | glycogen phosphorylase |
| 3,501,576 | IS*150* (+) +3 bp | intergenic (‑35/‑354) | *yhiO* ← / → *uspA* | universal stress protein UspB/universal stress global response regulator |
| 3,577,388 | Δ4,835 bp | IS*150*‑mediated | *[bisC]*–*hokA* | *[bisC]*, *yiaD*, *tkrA*, *yiaF*, *yiaG*, *cspA*, *hokA* |
| 4,478,040 | IS*150* (+) +3 bp | coding (518‑520/2292 nt) | *mdoB* ← | phosphoglycerol transferase I |
| 4,501,612 | C→T | E202K (GAG→AAG) | *lplA* ← | lipoate‑protein ligase A |
