# Supplementary material for: Genomic and phenotypic evolution of Escherichia coli in a novel citrate-only resource environment
Source: eLife. 2020 May 29;9:e55414. doi: 10.7554/eLife.55414 (PMC7299349; doi:10.7554/eLife.55414)
Supplement: Supplementary file 5. [file elife-55414-supp5.zip › S4File_genomes-by-environment/DM0-html/ZDBp901_minus_ZDB69.html]

Mutation Comparison


| Predicted mutations | | | | |
| --- | --- | --- | --- | --- |
| position | mutation | annotation | gene | description |
| 249,356 | C→A | intergenic (‑36/‑204) | *fadE* ← / → *lpcA* | acyl‑CoA dehydrogenase/phosphoheptose isomerase |
| 464,051 | IS*150* (+) +3 bp | coding (274‑276/528 nt) | *priC* ← | primosomal replication protein N'' |
| 681,779 | IS*150* (+) +3 bp | coding (513‑515/1665 nt) | *asnB* ← | asparagine synthetase B |
| 735,572 | A→C | S237A (TCC→GCC) | *gltA* ← | citrate synthase |
| 736,121 | C→T | E54K (GAA→AAA) | *gltA* ← | citrate synthase |
| 923,167 | IS*150* (+) +3 bp | coding (527‑529/831 nt) | *ybjR* → | predicted amidase and lipoprotein |
| 1,216,074 | (T)7→8 | coding (584/1212 nt) | *ycgF* ← | predicted FAD‑binding phosphodiesterase |
| 1,457,389 | Δ11,725 bp | between IS*150* | *hrpA*–*insJ‑2* | *hrpA*, *ydcF*, *aldA*, *gapC*, *insA‑12*, *insB‑12*, *cybB*, *ydcA*, *hokB*, *mokB*, *insK‑2*, *insJ‑2* |
| 3,109,394 | IS*150* (–) +4 bp | coding (245‑248/663 nt) | *yqjA* → | conserved inner membrane protein |
| 3,501,352 | +A :: IS*150* (+) +3 bp | coding (188‑190/336 nt) | *yhiO* ← | universal stress protein UspB |
| position | mutation | annotation | gene | description |
| 4,090,512 | G→A | R758H (CGT→CAT) | *rpoB* → | DNA‑directed RNA polymerase subunit beta |
| 4,122,754 | IS*150* (–) +3 bp | coding (448‑450/1602 nt) | *aceB* → | malate synthase |
| 4,134,638 | T→C | L198P (CTG→CCG) | *yjbB* → | predicted transporter |
| 4,256,901 | (CGCGG)3→2 | intergenic (‑768/‑1042) | *dcuR* ← / → *yjdI* | DNA‑binding response regulator in two‑component regulatory system with DcuS/hypothetical protein |
| 4,478,024 | IS*150* (–) +3 bp | coding (534‑536/2292 nt) | *mdoB* ← | phosphoglycerol transferase I |
