# Supplementary material for: Genomic and phenotypic evolution of Escherichia coli in a novel citrate-only resource environment
Source: eLife. 2020 May 29;9:e55414. doi: 10.7554/eLife.55414 (PMC7299349; doi:10.7554/eLife.55414)
Supplement: Transparent reporting form [file elife-55414-transrepform.docx]

***eLife’s* transparent reporting form**

We encourage authors to provide detailed information *within their submission* to facilitate the interpretation and replication of experiments. Authors can upload supporting documentation to indicate the use of appropriate reporting guidelines for health-related research (see [EQUATOR Network](http://www.equator-network.org/%20)), life science research (see the [BioSharing Information Resource](https://biosharing.org/)), or the [ARRIVE guidelines](http://www.plosbiology.org/article/info:doi/10.1371/journal.pbio.1000412) for reporting work involving animal research. Where applicable, authors should refer to any relevant reporting standards documents in this form.

If you have any questions, please consult our Journal Policies and/or contact us: [editorial@elifesciences.org](mailto:editorial@elifesciences.org).

**Sample-size estimation**

- You should state whether an appropriate sample size was computed when the study was being designed
- You should state the statistical method of sample size computation and any required assumptions
- If no explicit power analysis was used, you should describe how you decided what sample (replicate) size (number) to use

Please outline where this information can be found within the submission (e.g., sections or figure legends), or explain why this information doesn’t apply to your submission:

The number of populations evolved was chosen based on experimental microbial evolution convention and feasibility (incubator capacity). Most analysis were performed on all populations or randomly chosen clones therefrom. When only a subset of clones was used in analysis, the reasons for those selected are explained in the text.

**Replicates**

- You should report how often each experiment was performed
- You should include a definition of biological versus technical replication
- The data obtained should be provided and sufficient information should be provided to indicate the number of independent biological and/or technical replicates
- If you encountered any outliers, you should describe how these were handled
- Criteria for exclusion/inclusion of data should be clearly stated
- High-throughput sequence data should be uploaded before submission, with a private link for reviewers provided (these are available from both GEO and ArrayExpress)

Please outline where this information can be found within the submission (e.g., sections or figure legends), or explain why this information doesn’t apply to your submission:

All information on replication is stated in the text or figure legends as appropriate. All analytical details are provided in the text or figure legends, or supplements as appropriate. All analysis and statistical scripts have been deposited at www.datadryad.org (<https://doi.org/10.5061/dryad.7wm37pvpp>). RNA-Seq data have been deposited in the NCBI SRA under accession PRJNA553503. Genome sequencing data have been deposited in the NCBI SRA under accession PRJNA595472. Analysis code is also available at: <https://github.com/rohanmaddamsetti/DM0-evolution>.

**Statistical reporting**

- Statistical analysis methods should be described and justified
- Raw data should be presented in figures whenever informative to do so (typically when N per group is less than 10)
- For each experiment, you should identify the statistical tests used, exact values of N, definitions of center, methods of multiple test correction, and dispersion and precision measures (e.g., mean, median, SD, SEM, confidence intervals; and, for the major substantive results, a measure of effect size (e.g., Pearson's r, Cohen's d)
- Report exact p-values wherever possible alongside the summary statistics and 95% confidence intervals. These should be reported for all key questions and not only when the p-value is less than 0.05.

Please outline where this information can be found within the submission (e.g., sections or figure legends), or explain why this information doesn’t apply to your submission:

All statistical tests, analyses, and relevant summary statistics are described in the body of the text or in figure legends as appropriate.

(For large datasets, or papers with a very large number of statistical tests, you may upload a single table file with tests, Ns, etc., with reference to sections in the manuscript.)

**Group allocation**

- Indicate how samples were allocated into experimental groups (in the case of clinical studies, please specify allocation to treatment method); if randomization was used, please also state if restricted randomization was applied
- Indicate if masking was used during group allocation, data collection and/or data analysis

Please outline where this information can be found within the submission (e.g., sections or figure legends), or explain why this information doesn’t apply to your submission:

We allowed two initially identical sets of 12 populations each to evolved in either a novel, citrate-only resource environment or an ancestral, glucose and citrate environment. Samples were not allocated from a general population into experimental versus control groups, and this information therefore does not apply to our submission.

**Additional data files (“source data”)**

- We encourage you to upload relevant additional data files, such as numerical data that are represented as a graph in a figure, or as a summary table
- Where provided, these should be in the most useful format, and they can be uploaded as “Source data” files linked to a main figure or table
- Include model definition files including the full list of parameters used
- Include code used for data analysis (e.g., R, MatLab)
- Avoid stating that data files are “available upon request”

Please indicate the figures or tables for which source data files have been provided:

We have uploaded source data files with our submission as described below. These data and more are also reported in our Dryad submission. Please see <https://doi.org/10.5061/dryad.7wm37pvpp> for details. All analysis code may be found at: <https://github.com/rohanmaddamsetti/DM0-evolution>

Source data have been uploaded for Tables 1 and 2, Figures 2-14 and their supplements.

Within the Dryad submission, the relevant data files are as follows:

**Tables 1, 2— source data**: Copy number of amplifications affecting *citT, dctA, and maeA* in the ancestral and evolved clones: results/copy_number_table.csv

**Figure 2—source data 1**: All evolved mutations found in the DM0-treatment and DM25-treatment clones: results/genome-analysis/evolved_mutations.csv

**Figure 2—source data 2**: Classification and counts of mutations in the 264 LTEE genomes, published as Supplementary Table 4 of Tenaillon et al. (2016). data/rohan-formatted/nature18959-s4.csv

**Figure 3—source data 1**: Colony counts for fitness competitions of evolved populations in DM0 growth medium. EvolvedPopFitness-DM0-competitions.csv

**Figure 3—source data 2**: Colony counts for fitness competitions of evolved populations in DM25 growth medium. EvolvedPopFitness-DM25-competitions.csv

**Figure 4—source data 1**: Colony counts for fitness competitions of evolved clones in DM0 growth medium. EvolvedCloneFitness-DM0-competitions.csv

**Figure 4—source data 2**: Colony counts for fitness competitions of evolved clones in DM25 growth medium. EvolvedCloneFitness-DM25-competitions.csv

**Figure 5, supplement—source data**: Optical density (420nm) timeseries for REL606 growth in DM25 medium over more than 24 hours: data/rohan-formatted/REL606-DM25-48-hours.csv

**Figure 6 and Figure 6 supplements 1 and 2—source data**: Optical density (420nm) timeseries for DM0-evolved populations and their ancestors in DM0 and DM25 growth media. results/DM0-evolved-pop-growth.csv

**Figure 7 and Figure 7 supplements 1 and 2—source data**: Optical density (420nm) timeseries for DM0-evolved clones and their ancestors in DM0 and DM25 growth media. results/DM0-evolved-clone-growth.csv

**Figure 8 and Figure 8 supplements 1 and 2—source data**: Optical density (420nm) timeseries for DM25-evolved clones and their ancestors in DM25 growth medium. results/DM25-evolved-clone-growth.csv

We have uploaded source data files with our submission as described below. These data and more are also reported in our Dryad submission. Please see <https://doi.org/10.5061/dryad.7wm37pvpp> for details.

Source data have been uploaded for Tables 1 and 2, Figures 2-13 and their supplements, as well as for Supplementary File 2. Data for Figure 14 are provided in the Dryad submission.

Within the Dryad submission, the relevant data files are as follows:

**Figure 9—source data 1**: Optical density (420nm) timeseries for DM0-evolved populations and their ancestors in DM0 and DM25 growth media. results/DM0-evolved-pop-growth.csv

**Figure 9—source data 2**: Optical density (420nm) timeseries for DM0-evolved clones and their ancestors in DM0 and DM25 growth media. results/DM0-evolved-clone-growth.csv

**Figure 10—source data**. Micrograph image segmentation and classification by SuperSegger software. Nkrumah_MicroscopyData_DM0_project (all files ending with *.superSegger.txt")

**Figure 11—source data 1**: Counts of qualifying mutations in evolved clones. results/DM0-DM25-comparison-mut-matrix.csv

**Figure 11—source data 2**: Presence/absence of *dctA* and *maeA* amplifications in evolved clones. results/amp_matrix.csv

**Figure 11—source data 3**: Counts of qualifying mutations in non-mutator LTEE 50,000 generation clones. results/LTEE-mut_matrix.csv.

**Figure 11, supplement—source data**: Annotation of evolved GltA residues on PDB structure 1NXG. results/gltA-mutation-mapping/1nxg-mapping.pse

**Figure 12—source data 1**: Table of IS-element insertions in evolved genomes. results/genome-analysis/IS_insertions.csv

**Figure 12—source data 2**: Table of IS-element insertions in LTEE and Mutation Accumulation Experiment (MAE) genomes, originally published in Tenaillon et al. (2016). results/genome-analysis/LTEE_MAE_IS150_insertions.csv.

**Figure 13—source data 1**: Table of amplifications discovered by examining sequencing coverage in evolved genomes. results/amplifications.csv.

**Figure 14—source data**: RNA transcript quantification using *kallisto* software. results/RNAseq-analysis/XXXX/abundance.tsv, where XXXX is the name of the sample, as in 'ZDBp889_4'.
